# An Overview of Coumarin as a Versatile and Readily Accessible Scaffold with Broad-Ranging Biological Activities

**DOI:** 10.3390/ijms21134618

**Published:** 2020-06-29

**Authors:** Francesca Annunziata, Cecilia Pinna, Sabrina Dallavalle, Lucia Tamborini, Andrea Pinto

**Affiliations:** 1 Department of Pharmaceutical Science, University of Milan, via Mangiagalli 25, 20133 Milan, Italy; francesca.annunziata@unimi.it (F.A.); cecilia.pinna@unimi.it (C.P.); 2 Department of Food, Environmental and Nutritional Sciences, University of Milan, via Celoria 2, 20133 Milan, Italy; sabrina.dallavalle@unimi.it (S.D.); andrea.pinto@unimi.it (A.P.)

**Keywords:** coumarins, metabolites, biological activity, synthesis, natural sources

## Abstract

Privileged structures have been widely used as an effective template for the research and discovery of high value chemicals. Coumarin is a simple scaffold widespread in Nature and it can be found in a considerable number of plants as well as in some fungi and bacteria. In the last years, these natural compounds have been gaining an increasing attention from the scientific community for their wide range of biological activities, mainly due to their ability to interact with diverse enzymes and receptors in living organisms. In addition, coumarin nucleus has proved to be easily synthetized and decorated, giving the possibility of designing new coumarin-based compounds and investigating their potential in the treatment of various diseases. The versatility of coumarin scaffold finds applications not only in medicinal chemistry but also in the agrochemical field as well as in the cosmetic and fragrances industry. This review is intended to be a critical overview on coumarins, comprehensive of natural sources, metabolites, biological evaluations and synthetic approaches.

## 1. Introduction

**Figure 1:**
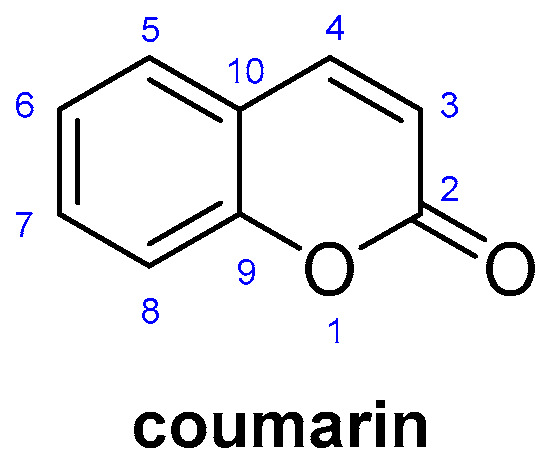
Structure of coumarin nucleus.

Coumarins are a wide family of secondary metabolites found in various species of plants (more than 1300 coumarins have been identified from natural sources, especially green plants) but also fungi and microorganisms [1,2]. The main pathway of coumarin biosynthesis occurs by shikimic acid route, via cinnamic acid, through phenylalanine metabolism [3]. The history of these natural products began 200 years ago—the name of the class derived from the plant *Coumarouna odorata* (*Dipteryx odorata*) from which the simplest member of this family, coumarin itself (Figure 1), was isolated by Vogel in 1820 [3,4]. Chemically speaking, coumarins are organic heterocycles and their nucleus is represented by benzo-α-pyrone (2*H*-1-benzopiran-2-one), whose systematic nomenclature was established by International Union of Pure and Applied Chemistry (IUPAC) [5].

Natural coumarins are subdivided in different classes based on their chemical diversity and complexity—simple coumarins, isocoumarins, furanocoumarins and pyranocoumarins (both angular and linear), biscoumarins and other coumarins such as phenylcoumarins (Table 1) [6].

Coumarins have several attractive features, such as low molecular weight, simple structure, high bioavailability, high solubility in most of the organic solvents and low toxicity, which, together with their multifaceted biological activities, ensure them a prominent role as lead compounds in drug research and development [2,6,7]. Coumarins exhibit several pharmacological effects including anticoagulant, antimicrobial, anti-inflammatory, neuroprotective, antidiabetic, anticonvulsant and antiproliferative [8]. Their importance is also clear in the food industry where their fungicide and antioxidant activities are investigated and exploited [9,10]. Moreover, some natural benzocoumarins show anti-algal activity [11]. Despite being recognized for their biological activities, coumarins present another important characteristic widely explored by the industry: their luminescent properties. The luminescence of some derivatives results from the intrinsic charge transfer properties of electron-rich conjugated π-π systems [3]. These compounds present a broad variety of applications, for example as photocleavable protecting groups or fluorescent probes [12]. 

This privileged scaffold serves as an important platform for the design of compound libraries in the search for new drug candidates. Here, we critically review the actual state of the art on natural and synthetic coumarins, focusing on the biological activity, structure–activity relationships (SARs), pharmacokinetics (PKs) and their potential applications in the pharma and agri-food sectors. Furthermore, an overall overview of the most common synthetic routes applied to obtain simple coumarins are provided, together with a further discussion on alternative, innovative and green synthetic methodologies.

### Metabolism

**Figure 2:**
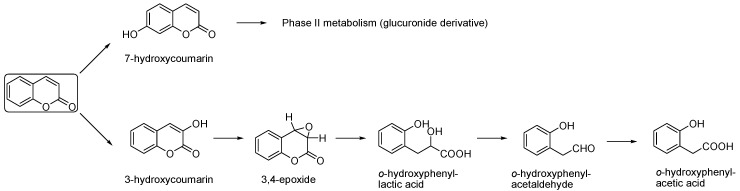
Coumarin metabolism.

Coumarin is metabolized by cytochrome P450-linked mono-oxygenase enzyme (CYP2A6) system in liver microsomes, which leads to hydroxylation; subsequently, the hydroxylated metabolite follows phase II conjugation reactions. Although coumarin could potentially be hydroxylated at all six possible positions (i.e., carbon atoms 3, 4, 5, 6, 7 and 8) (Figure 1), 7-hydroxycoumarin and 3-hydroxycoumarin are the main metabolites. The former one faces phase II conjugation reaction resulting in the glucuronide derivative, whereas 3-hydroxycoumarin can be further metabolized by ring splitting to form two products, *o*-hydroxyphenyllactic acid and *o*-hydroxyphenylacetic acid (Figure 2) [4,21]. Since the expression of CYP2A6 varies between individuals, due to genetic and environmental factors, an inter-individual variation in the metabolism of coumarin drugs is possible [4].

In a recent work, eight coumarin metabolites, which had not been identified previously, were detected by means of UPLC/Quadrupole-TOF tandem mass spectrometry in human urine [22]. Among them, positional isomers of 7-hydroxycoumarin glucuronide and 7-hydroxycoumarin sulphate were found. It was proposed that such isomers should bear the substituent in position 5, 6 or 8. Metabolites coming from a double hydroxylation and subsequent conjugation with glucuronic acid or sulphate group (and their isomers) were detected as well. Another metabolite was the one obtained by a double hydroxylation of the coumarin ring, followed by methylation and glucuronidation at the two newly generated hydroxyl groups. Finally, the *N*-acetylcysteine conjugated metabolite was identified. In this work, *o*-hydroxyphenylacetic acid was also detected in the samples, whereas free coumarin and *o*-coumaric acid were not found, indicating that coumarin was completely metabolized before excretion, after oral administration. This meant that *o*-coumaric acid found in human plasma [23] underwent a biotransformation process before being eliminated, probably leading to *o*-hydroxyphenylacetic acid.

## 2. Biological Activities

### 2.1. Antioxidant Activity

In a healthy human body, normal metabolic processes produce free radicals and other highly reactive species such as ions, molecules with unpaired electrons, Reactive Oxygen, Carbon, Nitrogen or Sulfur Species (ROS, RCS, RNS or RSS). When these species are overproduced, oxidative processes might cause cellular damage, affecting cellular components and causing ionic imbalance or mitochondrial disfunction [24]. The role of oxidative stress in different pathologies is well know: inflammation, cardiovascular diseases, cancer, diabetes and even neurodegenerative disorders often count oxidative damage among their pathological features [25,26,27]. Therefore, exogenous antioxidants might be useful in order to maintain the right concentration of radicals, reducing the amounts of free radicals and avoiding oxidative stress [28]. 

The antioxidant potential of natural and synthetic coumarins has been deeply investigated in the last years and it became clear that poly-hydroxy or phenolic coumarins are efficient antioxidants in biologicals systems [29]. Here below the most recent updates in this field are reported. 

**Figure 3:**
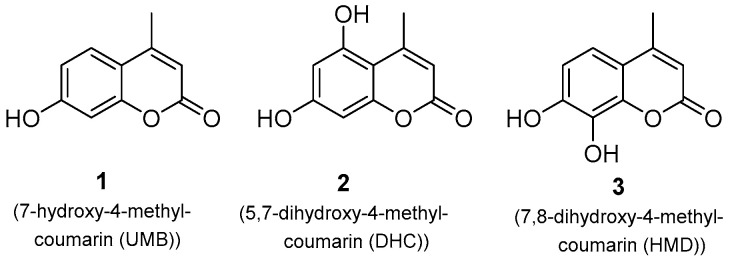
Coumarin structures reported by Couttolenc et al. [30].

In 2019, Couttolenc and collaborators studied the radical scavenging activity of three hydroxy-4-methylcoumarins (**1**–**3**, Figure 3) by means of experimental and theoretical methods [30]. 

**Figure 4:**
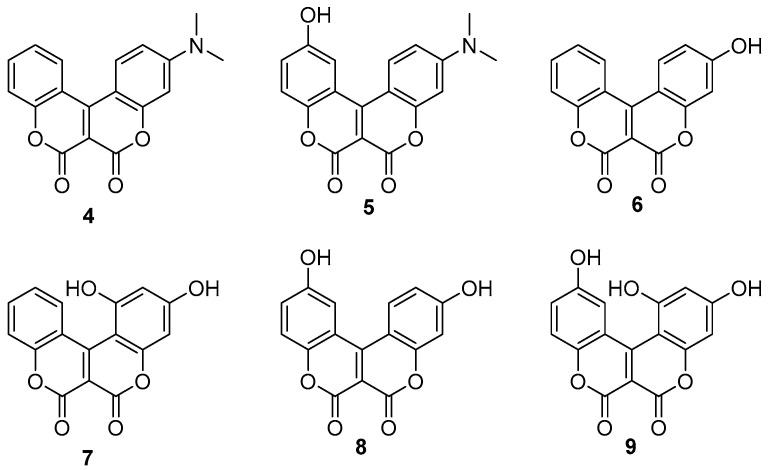
Chemical structure of coumarin-fused coumarins **4**–**9**.

Firstly, the scavenging activity of the compounds was evaluated on ABTS (2,2′-azino-bis (3-ethylbenzothiazoline-6-sulfonic acid) diammonium salt), DPPH (2,2-diphenyl-1-picrylhydrazyl) and galvinoxyl radicals as Trolox (a Vitamin E analogue) equivalent antioxidant capability (TEAC). The results showed that, whereas **1** did not exhibit radical scavenging activity, **2** resulted more active than Trolox against the ABTS•+ radical (EC_50_ 30.83 μM) and **3** displayed better antioxidant activity than Trolox against ABTS•+, DPPH and galvinoxyl radicals (EC_50_ values of 39.98, 150.99 and 13.19 μM, respectively). It is likely that such differences in antioxidant activity may rely on the differences in the relative positions of hydroxy groups [31]. Then, compound **3**, which showed the best scavenging activity, was evaluated for its primary antioxidant capacity. In this step, three reaction mechanisms were considered: single electron transfer (SET), hydrogen transfer (HT) and radical adduct formation (RAF), involving •OOH, •OOCH_3_ and •CH(OH)CH_3_ as radicals. These experiments were carried out in lipidic and aqueous media, in order to mimic membrane and intra-cellular environment [32], [33]. The results indicated that different mechanisms are involved depending by the medium and that positions 4, 7 and 8 of compound **3** are probably involved in HT mechanism, whereas positions 3, 4, 5 and 7 could be involved in RAF mechanism. Finally, the secondary antioxidant activity of compound **3** in aqueous media at physiological pH was evaluated. In the Haber-Weiss reaction (i.e., the reduction of copper in aqueous media and subsequent copper-catalyzed hydroxy radical formation), **3** was able to inhibit copper (II) reduction avoiding oxidative stress. It was found that hydroxy groups in position 7 and 8 are fundamental for the primary and secondary antioxidant activity in both lipid and aqueous media. Recently, Wang and co-workers investigated the antioxidant activity and the mechanism of the antiradical action of six coumarin-fused coumarins (**4**–**9**, Figure 4) previously synthesized by Xi and Liu [34,35]. 

Density functional theory (DFT) calculations were performed, followed by the examination of the primary mechanisms, including HT, electron transfer-proton transfer (SET-PT) and sequential proton loss transfer (SPLET). The most stable conformation of all the compounds was a non-planar structure, due to the steric repulsion of the groups in positions 5 and 5′. Such conformation was retained by the correspondent anions and cation radicals (ArO^−^, ArOH^+•^). HT process resulted the most significant in gas or non-polar phase, where compound **9** showed the highest activity. The HT path was possible for compounds **7**, **8** and **9**, having two or three OH groups, whereas **4** resulted inactive due to the absence of OH groups; compound **5**, with only the 6-OH group, was less active than other compounds and **6** could merely trap DPPH radical with a small rate constant. A second HAT process was possible only for compound **9** and this finding could explain the higher activity of this molecule. In polar media, SPLET mechanism was favored—in this case the studied compounds were more efficient than Trolox in the deprotonation step and, among them, compound **7** resulted to be the most promising, being more prone than the other compounds to deprotonate in a polar environment.

**Figure 5:**
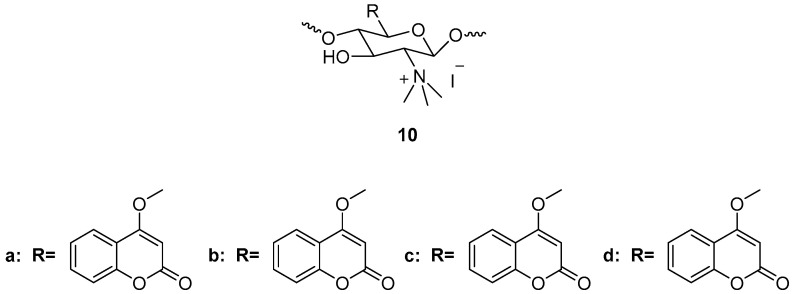
Chitosan derivatives **10a**–**d**.

The antioxidant potential of coumarin nucleus can be exploited in the production of new hybrid compound with enhanced antioxidant activity. A recent example of this strategy is the synthesis of a new series of chitosan derivatives (**10a**–**d**, Figure 5) containing the coumarin nucleus, achieved by Li and collaborators [36]. 

The antioxidant potential of compounds **10a**–**d** was investigated by evaluating the inhibition of lipid peroxidation, metal ion chelation and free-radical scavenging activity. Since both chitosan and coumarins have antioxidant properties themselves, the synthesized compounds were expected to show a stronger activity. Lipid peroxidation inhibition was determined by quantifying thiobarbituric acid-reactive substance (TBARS), using linoleic acid as a reference compound [37]. The results displayed the ability of the synthesized molecules to inhibit TABRS in a concentration-dependent manner; compound **10d** emerged as the most active (IC_50_ = 0.11 mg/mL), showing a more efficient scavenging activity than chitosan alone (IC_50_ = 0.38 mg/mL). Then, the radical-scavenging activity was evaluated. For this purpose, free radicals •OH, DPPH and O_2_•^−^ were used. Compounds **10a**–**d** showed a stronger •OH scavenging activity (IC_50_ = 0.09–0.12 mg/mL) compared to that of chitosan. These results suggested that the coumarin moiety strongly enhances chitosan antioxidant properties. Since the chelating ability of antioxidants prevents oxidative stress by avoiding •OH production, iron-chelating properties of the new compounds were also evaluated through measurements of inhibition of ferrozine-Fe^2+^ complex formation. Again, the obtained results (IC_50_ = 0.02–0.04 mg/mL) were better than those of chitosan alone. Finally, cytotoxicity of the synthesized compounds was tested: no toxic effects were detected on 3T3-L1 and HHL-5 cells. Interestingly, 3T3-L1 cells showed an increase in their viability, probably because of the antioxidant activity of the tested compounds. 

**Figure 6:**
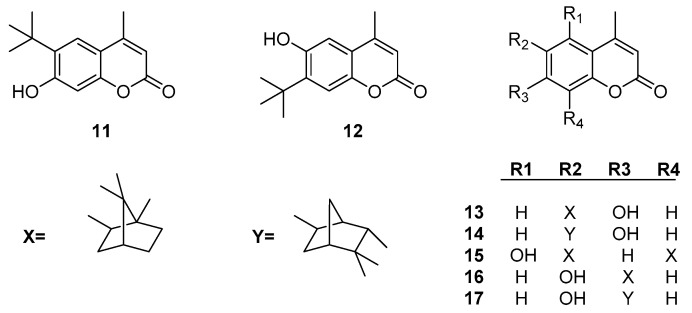
Chemical structures of *tert*-butylcoumarins **11** and **12** and terpenylcoumarins **13**–**17**.

A similar approach was followed by Popova and co-workers, who designed and synthesized a series of 4-methylcoumarins with *tert*-butyl, isobornyl and isocamphyl substituents (**11**, **12**, **13–17**, Figure 6) [38].

The synthesized compounds were evaluated in terms of antioxidant, membrane-protective (MPA) and radical-scavenging (RSA) activities in vitro. All the tested compounds, at a concentration of 100 μM, exhibited good inhibitory activity against lipid peroxidation products (LPO) formation (IC_50_ = 3.33–7.12 nM), whereas 7-hydroxy-4-methylcoumarin, used as reference compound, showed no activity. The scavenging activity, evaluated using DPPH, strictly depended on the structure: only isobornyl derivatives showed moderate activity in the DPPH assay (compound **15** showed RSA% = 57.48 ± 0.60 at 100 μM; RSA% = 87.95 ± 0.22 at 500 μM). Moreover, the protective activity towards cell membrane was assessed, measuring the inhibitory activity against H_2_O_2_-induced hemolysis of red blood cells (RBCs). In all the experiments, the most promising compound was **15**, having two isobornyl moieties.

From these studies, it appears clear that coumarin derivatives show a great potential as antioxidant, membrane-protective and radical-scavenging compounds and that their activity depends mainly on the number and the position of the hydroxy groups.

### 2.2. Anticancer Activity

The term “cancer” defines a wide range of diseases caused by the accumulation of mutations and characterized by a multi-step process, involving many different factors which may not directly cause cancer themselves but can increase the chances of genetic mutations [39,40]. 

**Figure 7:**
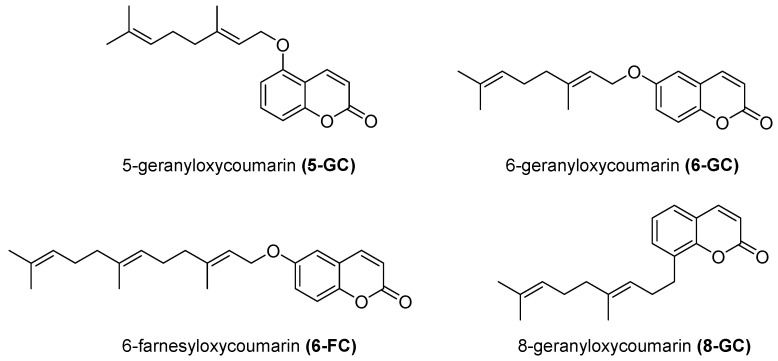
Structure of the most active *O*-prenylated coumarins.

Recently, Maleki et al. have synthesized eighteen *O*-prenylated coumarin derivatives and tested them on HeLa cervical cancer and HDF normal cells by MTT assay [41]. The most promising compounds are reported in Figure 7.

The results represent a good starting point for the design of novel derivatives, because most of the examined compounds exhibited selective toxicity on HeLa cells (IC_50_ values between 136.4 ± 1.90 μM and 172.2 ± 1.80 μM after 24 h), whereas no negative effects on HDF normal cell’s growth was detected. SAR studies proved that the substitution on C6 position of coumarin nucleus provided the best anticancer activity, followed by substitution on C8. In addition, it was found that the cytotoxic properties of *O*-prenylated coumarins depends on the length of the prenyl chain, which increases the lipophilicity of the molecule, thus facilitating its penetration into the cells. 

**Figure 8:**
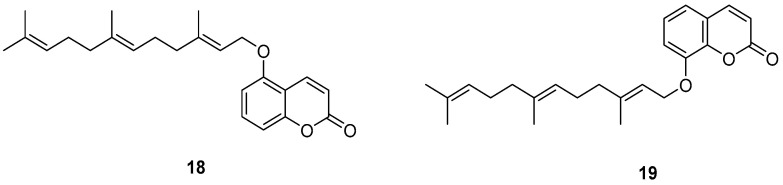
Chemical structure of 5-farnesyloxycoumarin (**18**) and 8-farnesyloxycoumarin (**19**).

The cytotoxic activity of prenylated-coumarin derivatives had been evaluated also by other groups. Recent studies have shown a functional role of lipoxygenases (LOXs) in carcinogenesis, precisely in prostatic cancer and the capability of 5- farnesyloxycoumarin (**18**, Figure 8) to inhibit 15-LOX-1 [42,43,44].

Starting from these observations, Orafaie and collaborators investigated the inhibitory activity of compound **18** on 15-LOX-1 [45]. The cytotoxic effects of **18** were evaluated by means of MTT assay on two carcinoma cell lines (PC-3 and DU145) and a normal cell line (HFF3), using 4-MMPB (4-methyl-2-(4-methylpiperazinyl) pyrimido [4,5-b] benzothiazine) as reference compound. When PC3 and DU145 human PCa cells were treated with different concentrations of both 4-MMPB and **18** for 24, 48 and 72 h, a dose-dependent and time-dependent decrease in the survival of the cells was exhibited. PC3 cells resulted to be more sensitive than DU145 cells to both inhibitors (IC_50_ in μg/mL for compound **18** on PC3 cells: 24 h, 40.1 ± 7.9, 48 h, 27.05 ± 3.9, 72 h, 26.43 ± 2.1; DU145 cells: 24 h, 98.14 ± 48.3, 48 h, 62.5 ± 17.3, 72 h, 41.85 ± 7.8; IC_50_ in μg/mL for 4-MMPB: PC3 cells: 24 h, 32.01 ± 3.2, 48 h, 25.47 ± 1.9, 72 h, 18.97 ± 2.8; DU145 cells: 24 h, 35.22 ± 1.9, 48 h, 27.84 ± 2.22, 72 h, 19.52 ± 4.92). Moreover, compound **18** had no significant anti-proliferative activity on normal cells. Concerning the mechanism of action, it was found that 5- farnesyloxycoumarin acts as a cytotoxic agent causing chromatin condensation and DNA damage and induces the arrest of the cell cycle in G_0_/G_1_ phase. A similar study was carried out on 8-farnesyloxycoumarin (**19**, Figure 8) by Hosseinymehr and collaborators [46]. Again, the coumarin derivative showed inhibitory activity on 15-LOX-1 in PC3 and DU145 cell lines, thus inducing apoptosis of the cancer cell, with the same mechanism of compound **18**. PC3 cells resulted to be more sensitive to **19** inhibition than DU145 cells; IC_50_ values of **19** on PC3 cells were similar to those of cisplatinum (PC3 IC_50_ in μg/mL 24 h, 34.98 ± 95.57, 48 h, 14.92 ± 40.76, 72 h, 14.12 ± 38.58). 

**Figure 9:**
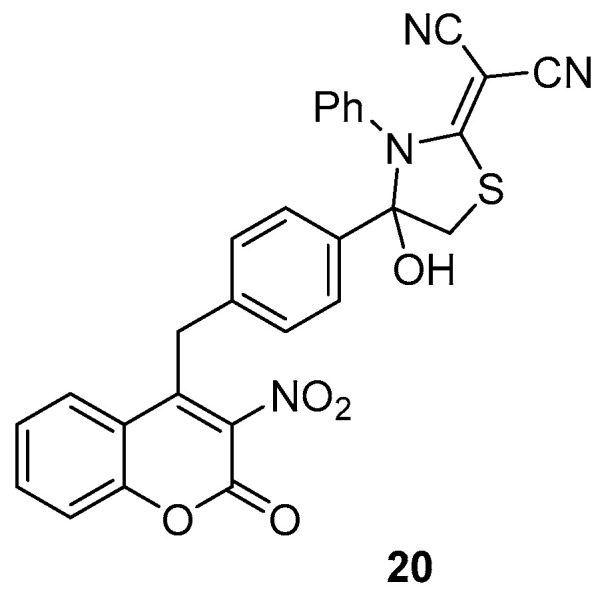
4-arylamino-3-nitrocoumarin derivative **20**.

Halawa et al. synthesized and characterized a new series of 4-arylamino-3-nitrocoumarin analogues from 4-hydroxycoumarin and tested them on the human cervix carcinoma cell line KB-3-1 [47]. These compounds were found to target the DNA-Topo I (human Topoisomerase I) complex, thus blocking cell replication and leading to cell death. Among this series, thiazolidinylidene derivative **20** (Figure 9) containing a malononitrile fragment exhibited the best cytotoxic activity with an IC_50_ value of 21 μM. The cytotoxicity of **20** was explained also by docking studies which highlighted that it forms important H-bonds with Arg364, Asp533, Gln633 and 5′-thio-2′ deoxyguanosine phosphonic acid of the DNA backbone.

**Figure 10:**
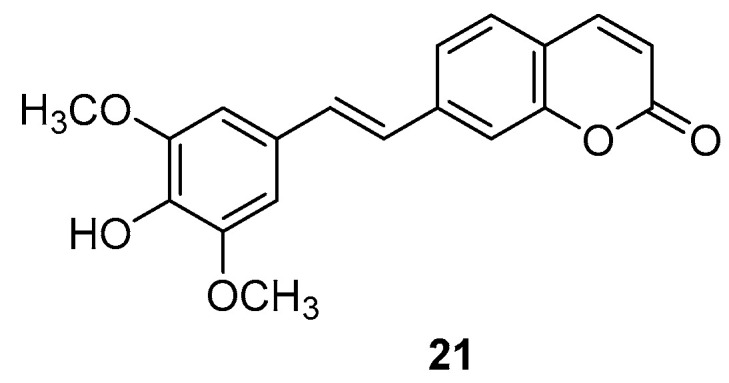
Chemical structure of styrylcoumarin **21**.

Herrera et al. synthesized a series of 3- and 7-styrylcoumarins, some of which showed anti-proliferative activity on SW480 human colon adenocarcinoma cells [48]. Among them, 7-(4-hydroxy-3,5-dimethoxystyryl)-2*H*-chromen-2-one (**21**, Figure 10) showed the highest activity (IC_50_ = 1.01 μM) as it was capable of inducing apoptosis in SW480 cells, probably by modulating the tumor-suppressor protein p53. The new compound was also tested in vivo, demonstrating to be able to inhibit the early progression of colon adenocarcinoma [49]. 

**Figure 11:**
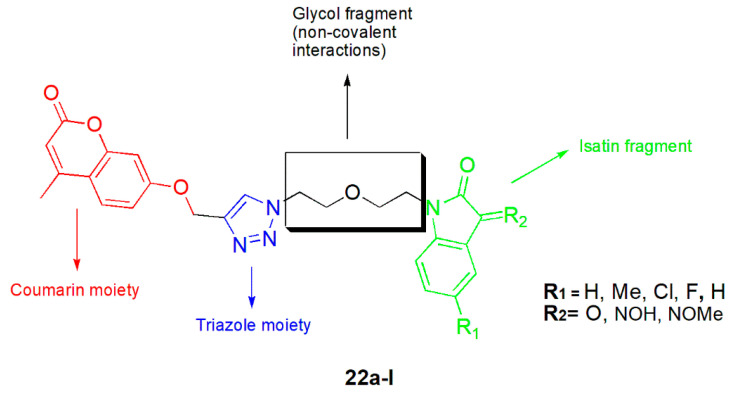
General structure of diethylene glycol tethered isatin-1,2,3-triazole-coumarin derivatives **22a-l**.

As coumarin nucleus can be widely decorated, it can be used in the synthesis of hybrid compounds, targeting different proteins involved not only in tumor growth but also in metastatic and angiogenetic processes. In this context, Diao and collaborators synthesized a series of diethylene glycol tethered isatin-1,2,3-triazole-coumarin derivatives (**22a-l**, Figure 11) [50]. Several isatin-based or 1,2,3- triazole or coumarin-based compounds (semaxanib, carboxyamidotriazole and STX64) are involved in clinical trials or have already been used the treatment of various cancers (as colon-rectal, prostatic, endometrial and breast cancer) [51,52], whereas isatin-1,2,3-triazole-coumarin derivatives showed activity against different cancer types. In addition, SAR studies demonstrated that the linker between isatin and 1,2,3-triazole influences the activity [52,53] and that hydrogen bonds are fundamental for the biological activity [54]. These evidences guided Diao and collaborators in the choice of diethylene glycol as a linker [47]. Ten compounds were tested on HepG2 (liver carcinoma), HeLa (cervical cancer), A549 (lung adenocarcinoma), DU145 (prostatic cancer), SKOV3 (ovarian carcinoma), MCF-7 (breast cancer) and drug-resistant MCF-7/DOX (doxorubicin-resistant MCF-7) human cancer cell lines. All the synthesized compounds showed weak to moderate in vitro activity against all the cell lines, therefore further SAR studies are necessary for the obtainment of new, more active compounds. 

**Figure 12:**
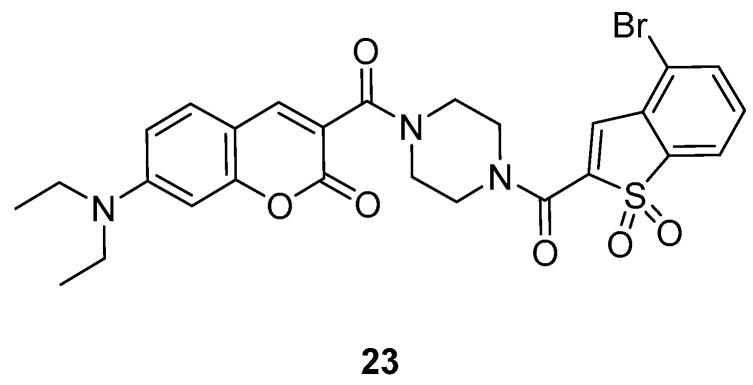
Chemical structure of compound **23**.

Cai et al. developed a series of fluorescent coumarin-benzo[*b*]thiophene 1, 1-dioxide conjugates [55]. These compounds act on STAT3, which is involved in the regulation of mitochondrial apoptotic pathway [56]. In particular, they hypothesized that the inhibition of phosphorylation of Tyr705 and Ser727 might prevent STAT3 activation. Four STAT3 over-activated human cancer cell lines (i.e., human breast carcinoma MDA-MB-231 and MCF-7 cells, human colonic carcinoma HCT-116 cells and human hepatocellular carcinoma HepG2 cells) were selected to assess the biological activity of the newly-synthesized compounds. Compound **23** (Figure 12) showed high potency in inducing cancer cell apoptosis and ROS generation, inhibiting STAT3 phosphorylation on Tyr705, affecting mitochondrial membrane potential and preventing STAT3 DNA-binding activity. In addition, **23** inhibited implanted 4T1 breast cancer growth in vivo. The antiproliferative effects of compound **23** on normal cells were investigated by MTT assays comparing it to the STAT3 inhibitor Stattic, which showed growth inhibition activity in both tumor and normal cells, whereas compound **23** exhibited selective inhibitory activity against cancer cells (IC_50_ 14.62 μM for MCF-10A cells and IC_50_ 35.60 μM for L02 cells), indicating a higher selectivity than Stattic. 

### 2.3. Carbonic Anhydrase Inhibition

**Figure 13:**
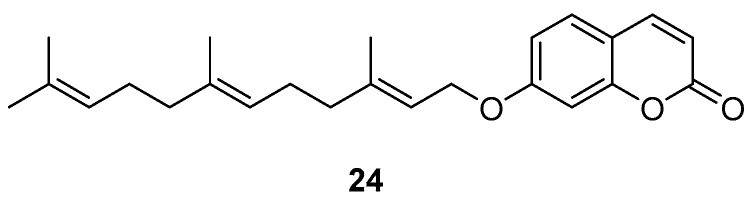
Chemical structure of umbelliprenin (**24**).

Carbonic anhydrases (CAs) are ubiquitous metalloenzymes that catalyze the reversible hydration of carbon dioxide to bicarbonate and protons, which is an essential physiological reaction [57]. Thus, this enzyme is involved in a wide range of physiological and pathological processes (pH regulation, CO_2_ homeostasis, respiration, bone resorption and tumorigenesis) [58] and its deregulation by means of carbonic anhydrases inhibitors (CAIs) may be useful in the treatment of many disorders [59,60,61]. The ideal CA inhibitor would selectively act against those isoforms (*h*CA IX, XII, for instance) related to a certain disease [62,63]. In this context, coumarins emerged as potential atypical *h*CA ligands that, unlike classical *h*CAIs, do not need to chelate the prosthetic zinc ion but, after binding the catalytic site, are hydrolyzed to the corresponding 2-hydroxy cinnamic acid derivatives, the actual inhibitors [64,65]. Some studies highlighted that coumarins are capable of binding at the entrance of *h*CA catalytic site, blocking the enzyme activity. Furthermore, 7-hydroxycoumarins derivatives showed good selectivity toward IX and XII isoforms over *h*CA I and II and, in some cases, they exhibited cytotoxic activity on cancer cells [66,67,68,69]. Many efforts have been made in this direction and the most recent results are here reported. Fois and collaborators have recently investigated the selective inhibitory activity towards CA IX and CA XII of extracts of *Magydaris pastiacea* seeds, from which they isolated ten linear furocoumarins, four simple coumarins and a new angular dihydrofurocoumarin [70] All the mentioned compounds were tested against four isoforms of *h*CA: both extracts showed selective activity towards CA IX and XII whereas no effect was seen on isoforms I and II at a concentration of 100 ng/mL. The most promising compound was umbelliprenin (**24**, Figure 13), highly active (K_i_ = 5.8 nM) against CA XII and highly selective over isoforms I and II. 

Umbelliprenin (**24**) was then tested on HeLa cancer cells in order to evaluate its cytotoxic activity and resulted to possess a moderate cytotoxicity (IC_50_ = 75 μM) proving to be able to inhibit tumor growth, angiogenesis and metastasis formation in mice (after i.p. administration). It is noteworthy that CA IX and XII are overexpressed in tumor cells under hypoxic conditions, whereas the mentioned tests were carried out under normoxic conditions, which could explain the moderate cytotoxic activity of the isolated compound. 

**Figure 14:**
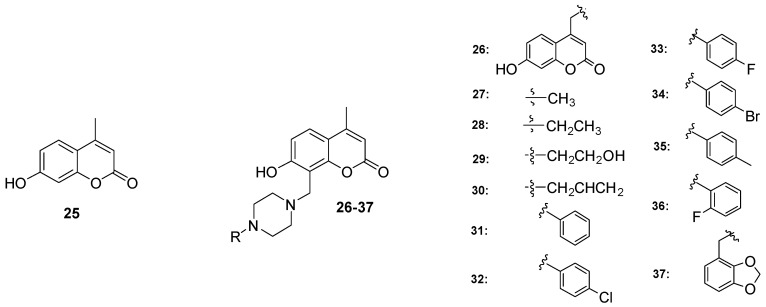
Chemical structure of compound **25** and piperazine derivatives **26**–**37**.

In 2019, starting from 4-methylumbelliferone (**25**, Figure 14), Buran and co-workers synthesized a series of 8-substituted coumarin-based compounds bearing alkylpiperazine and arylpiperazine chains (**26**–**37**, Figure 14), and evaluated their inhibitory activity against *h*CA I, II, IX and XII [71]. All the tested compounds were able to inhibit *h*CA isoforms IX and XII, without showing any inhibitory activity towards the cytosolic isoforms I and II up to a 10 μM concentration. The test pointed out that these compounds had higher affinity for *h*CA XII over IX and, except for compound **36** (K_i_ (*h*CA XII) = 26.4 nM), they all had K_i_ values comparable to those of the reference drug acetazolamide (K_i_ (*h*CA XII) = 5.7 nM).

It is remarkable that the substitution of C8 position of 4-methylumbelliferone (**25**) did not have any influence on inhibition of *h*CA XII, suggesting that no significant interaction may be achieved between the side chains of compounds **26**–**37** and the catalytic site of isoform XII. On the other hand, alkylpiperazine derivatives showed better activity on *h*CA IX when compared with the other synthesized compounds, being compound **30** the one with the highest activity among them (IC_50_ = 27.1 nM). Similar results were obtained by many other groups that have recently synthesized coumarin-based compounds and evaluated them as *h*CA IX and XII inhibitors. Sulphocumarins, bis-coumarins and coumarins 1,3,4-oxadiazole derivatives are some examples [72,73,74]. 

### 2.4. Antibacterial Activity

The battle against infections and multidrug resistant bacteria is almost certainly one of the most challenging issue that the scientific community and the whole mankind will face in the near future. Multidrug resistant (MDR) bacteria are defined as non-susceptible strains to one or more antimicrobials on three or more antimicrobial classes, whereas strains that are non-susceptible to all antimicrobials are classified as extremely drug-resistant strains [75,76]. The plant kingdom provides a virtually endless source of novel chemicals and scaffolds, such as polyphenols and coumarins [77]. In 2005, the antibacterial potency of nearly 50 coumarin derivatives, natural and synthetic, was evaluated and then correlated by a SAR study. Bacterial susceptibility to coumarins was evaluated by determining the minimal inhibitory concentration (MIC) and minimum bactericidal concentration (MBC), considering active compounds exhibiting MIC values ranging from 62.5 to 2000 μg/mL. Among the active compounds, osthenole (a natural coumarin having also the anti-inflammatory activity) showed the most potent activity with a MIC of 62.5 μg/mL against *S. aureus* and *B. cereus* [78]. 

**Figure 15:**
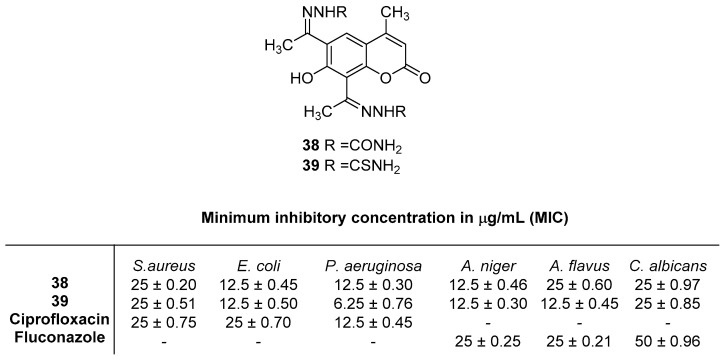
Chemical structure and minimal inhibitory concentration (MIC) values of antibacterial coumarins proposed by Nagamallu and colleagues [79].

In 2015, Nagamallu and co-workers exploited the Vilsmeier-Haack reaction to obtain a series of novel pyrazole-containing coumarins and then evaluated their antioxidant and antibacterial activities [79]. Among the series, two compounds (**38** and **39**, Figure 15) showed a good antibacterial and antifungal activity, with MIC values comparable with the ones of ciprofloxacin (positive control against bacteria species) and fluconazole (positive control against fungi strains).

Following the idea that the introduction of an additional coumarin nucleus on a parent coumarin molecule could improve the pharmacological activity (i.e., dicumarol as anticoagulant), in 2017, Chougala and colleagues designed and synthesized a series of bis-coumarin derivatives [80]. 

**Figure 16:**
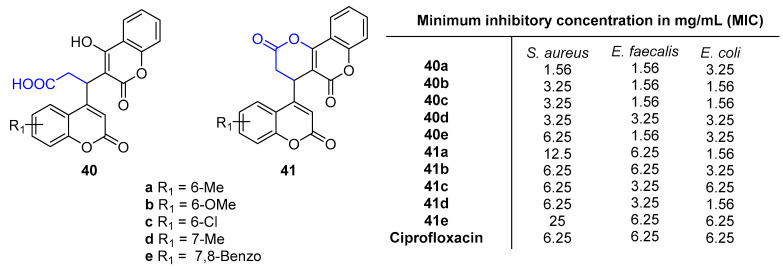
Coumarin dimer scaffolds proposed by Chougala and colleagues and the corresponding MIC values [80].

Figure 16 shows the common scaffolds of the bis-coumarins synthesized using l-proline as catalyst in a multi-component reaction approach. 

The antibacterial potency of the compounds was evaluated against Gram-positive and Gram-negative strains, comparing the MIC with ciprofloxacin and most of the newly synthesized compounds showed modest to good inhibiting activity against the tested microorganisms. Compounds **40a**–**e** (Figure 16) were highly active and more potent than ciprofloxacin against *S. aureus* and *E. faecalis*, whereas **41c** and **41d** were active only against Gram-positive *E. faecalis*. Actually, compounds **41a**–**e** were the most promising against *E. coli*.

Once established the broad spectrum of action of the coumarin nucleus, various researchers focused their attention on the activity against multidrug resistant strains, in particular on the ESKAPE pathogens, the coterie that escape the lethal action of antibiotics: *Enterococcus faecium*, *Staphylococcus aureus*, *Klebsiella pneumoniae*, *Acinetobacter baumanii*, *Pseudomonas aeruginosa* and *Enterobacter species* [81]. In 2017, Naik et al. synthesized and evaluated against *S. aureus* and other bacterial strains a series of 3,4-dihydropyrimidinone-coumarin analogues, measuring the MIC values and comparing them to ciprofloxacin [82]. 

**Figure 17:**
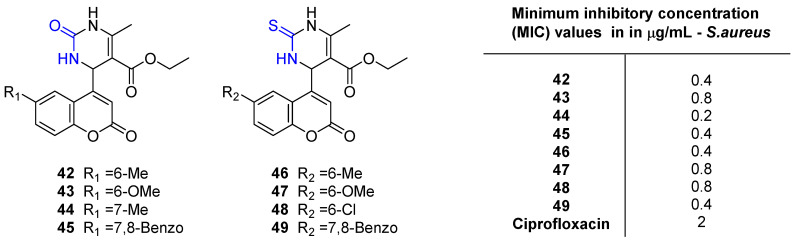
3,4-dihydropyrimidinone-coumarin analogues and their MIC values against *S. aureus*.

The structures and the MIC values for compounds **42**–**49**, the most promising derivatives, are reported in Figure 17: the substitution at C6 position seemed to be excellent for the activity and able to modulate the potency, decreasing the efficiency in the order of -CH_3_ > 7,8-Benzo > -Cl > -OCH_3_. Furthermore, it was revealed from docking studies that that one hydrogen atom and two oxygen atoms of 3,4-dihydropyrimidinone substituted coumarins form interactions with the active site residues of *S. aureus* gyrase, indicating that the presence of hydrogen bond acceptor and donor groups may enhance antimicrobial activity.

**Figure 18:**
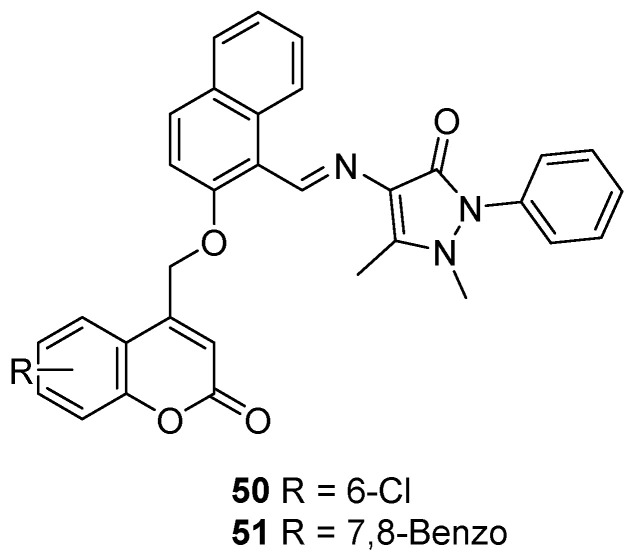
Coumarin-pyrazole hybrids and their MIC values against *S. aureus*.

In 2018, Chavan and Hosamani proposed a facile method for the microwave assisted synthesis of a series of pyrazole-containing coumarins and tested their antibacterial and anti-inflammatory activities [83]. The researchers evaluated the in vitro antibacterial activity of the newly synthesized compounds through agar-well diffusion method against two Gram-positive (*Bacillus subtilis* (ATCC no. 23857) and *Staphylococcus aureus* (ATCC-29213)) and two Gram-negative (*Escherichia coli* (ATCC-25922) and *Pseudomonas aeruginosa* (ATCC No.25619)) bacterial strains [84]. The MIC values were compared to those of ciprofloxacin and all the compounds showed significant antibacterial activity. In particular, compounds **50** and **51** (Figure 18), showed an excellent activity against *S. aureus* (MIC 0.78 μg/mL and MIC 1.562 μg/mL, respectively). Docking studies were in good agreement with the biological results. 

**Figure 19:**
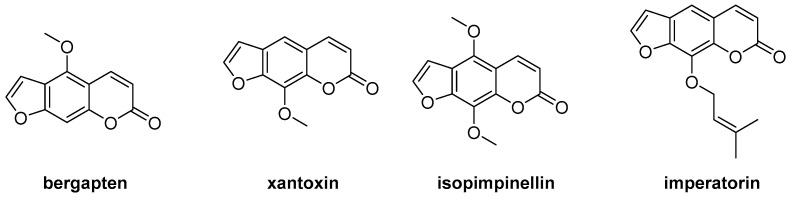
Coumarins isolated from *Rutacea* species.

In 2017, Madeiro and co-workers focalized their interest towards the antibiotic activity of coumarins isolated from *Rutacea* species (Figure 19) [85]. Bergapten, xantoxin, isopimpinellin and imperatorin did not exhibit any antibacterial activity, even at the highest concentration, against *S. aureus* strains resistant to tetracycline, erythromycin and norfloxacin. However, their role as modulator of other antibiotics seemed quite promising, because isopimpinellin and imperatorin reduced the MIC of erythromycin, tetracycline and norfloxacin. Nevertheless, more detailed research is necessary in order to enable the use of these natural products as adjuvants to antimicrobial agents.

**Figure 20:**
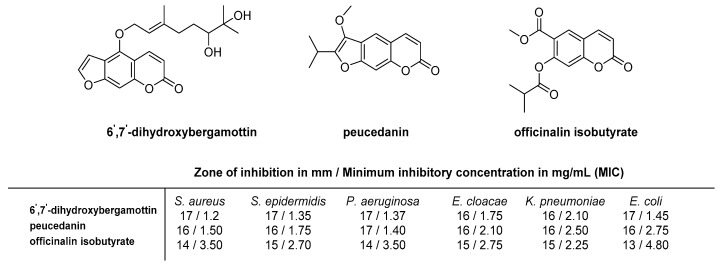
Linear coumarins isolated from the fruits of *Peucedanum luxurians* Tamamsch, zone of inhibition (mm) and minimum inhibitory concentration (MIC) values.

In 2018, Widelsky and co-workers isolated some similar linear furanocoumarins from the fruits of *Peucedanum luxurians* Tamamsch, with more encouraging results. Plants of the *Peucedanum* genus have been used for centuries as antibacterial agents and, for some of them, the activity was confirmed by biological and pharmacological studies on plant extracts and on a few isolated compounds [86]. All the six isolated compounds showed a broad growth-inhibitor activity against several bacteria strains but three of them (Figure 20) resulted particularly interesting. 6′,7′-Dihydroxybergamottin was the most active against all the bacteria strains tested, probably because of the aliphatic chain in C5 position; similar activity was noticed for peucedanin and officinalin isobutyrate. 

Liu and colleagues made a huge effort to synthesize and identify coumarin-pyrazole carboxamide derivatives as potential topoisomerase-II inhibitors: 70 novel compounds were obtained and evaluated [87]. 

**Figure 21:**
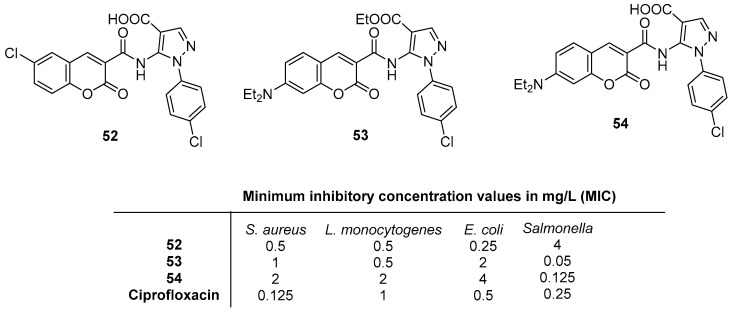
Selected coumarin-pyrazole carboxamide derivatives developed by Liu et al. and their MIC values [87].

Three of them (**52**–**54**, Figure 21) were endowed with promising antimicrobial activities. In particular, compound **52** showed a considerable inhibitory activity compared with ciprofloxacin against *Escherichia coli* and compound **53** exhibited excellent antibacterial activity against *Salmonella typhi*. The selected compounds exhibited also potent inhibition against Topo II and Topo IV with IC_50_ values in the range 9.4–25 mg/L. 

### 2.5. Antifungal Activity

Fungal diseases are a well-known plague for animal and plant worlds but a more hidden menace for human health. More than 90% of all reported fungal-related deaths results from species that belong to one of four genera: *Cryptococcus*, *Candida*, *Aspergillus* and *Pneumocystis* [88,89,90]. Coumarin derivatives are endowed with antifungal activity, potentially useful in both pharma and agri-food sectors. In this paragraph, we will focus on the recent progresses in the development of novel antifungal drugs for human use whereas agri-food applications can be found in Section 3.2, *Food systems*. 

**Figure 22:**
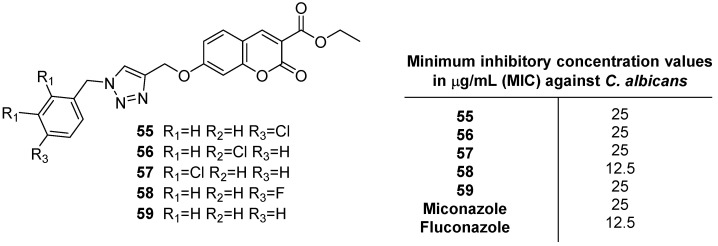
Antifungal coumarins.

Diseases by *Candida* species, a family of fungi that normally live on the host epithelial species but can initiate a fatal infection in particular cases like immunodeficiency, are the fourth most common cause of nosocomial blood-stream infections. Despite several species of *Candida* can cause disease, *Candida albicans* prevails in term of incidence [88,91,92]. Therefore, in 2016, Shaik and colleagues designed a novel series of coumarin derivatives conjugated with 1,2,3-triazole moieties, on the basis of a previous work by Shi and Zhou and of the common use of azoles as antifungal drugs [93,94]. The antifungal potency of the novel compounds was tested against *Candida albicans* and other four fungal pathogens (i.e., *Fusarium oxysporum, Aspergillus flavus, Aspergillus niger* and *Cryptococcus neoformans*); MIC values were evaluated and compared to MIC of the reference compounds miconazole and fluconazole. Compounds **55**–**57** and **59** (Figure 22) were found to be equipotent to miconazole against *C. albicans* and compound **58** was found to be two-fold more active compared with miconazole and equipotent to fluconazole.

Furthermore, molecular docking studies showed that these compounds have a high affinity toward the active site of enzyme P450 cytochrome lanosterol 14α-demethylase and analysis of ADME parameters confirmed their drug-like properties [94]. 

**Figure 23:**
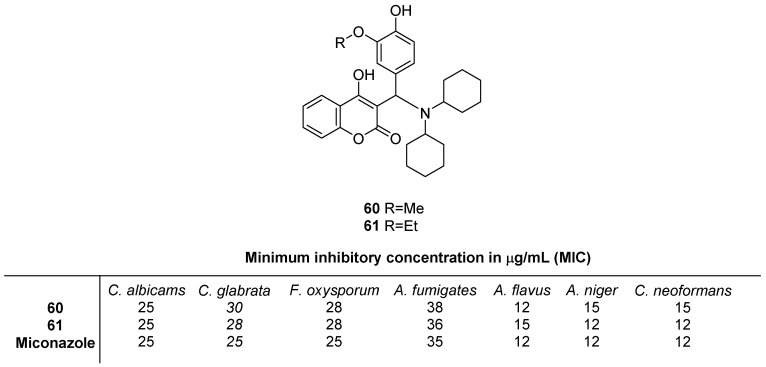
Antifungal coumarins synthesized under solvent-free conditions and exploiting ionic liquid as catalyst.

In 2017, fifteen novel coumarin derivatives were synthesized by Tiwari et al., under solvent free conditions and exploiting the ionic liquid [Et_3_NH][HSO_4_] as a catalyst [95]. The compounds were tested both for their antifungal and antibacterial activities and, among the series, compounds **60** and **61** resulted to be the most potent as fungicides (Figure 23). The MIC values observed for compound **60** and **61** were comparable to the standard drug miconazole. 

Further studies demonstrated that compound **60** acts by inhibiting ergosterol biosynthesis in *C. albicans*. Molecular docking studies revealed, as for previous study on compounds **55**–**59**, a highly spontaneous binding ability of the tested compounds to the active site of lanosterol 14α-demethylase, which suggests that the tested compounds inhibit the synthesis of this enzyme. Moreover, in silico ADMET properties were evaluated and demonstrated that all the compounds exhibited a good percent absorption (% ABS) ranging from 84.9% to 100%. Moreover, these compounds had proven to be safe after in vitro toxicity, in vivo acute oral toxicity and behavioral studies. 

**Figure 24:**
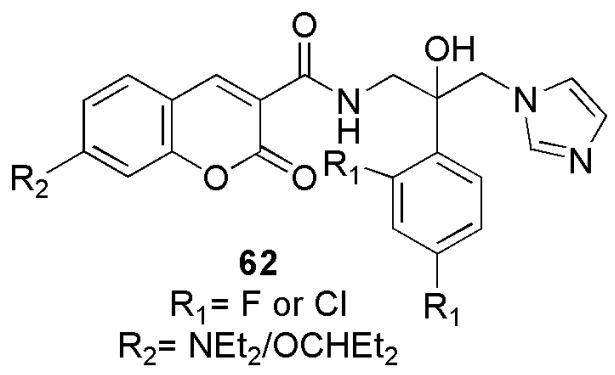
Coumarin derivatives conjugated to an imidazole-containing moiety.

Coumarin-based antifungal azoles had been further investigated by Elias and co-workers, who, in 2019, developed a series of 11 coumarins conjugated with 1,2,4-triazole and imidazole motifs [96]. The analysis of the biological effects of these novel chemical entities highlighted that imidazole-based derivatives (Figure 24) were more efficient against several *Candida* strains compared to 1,2,4-triazole derivatives. Moreover, the mode of action of the two classes of compounds were different. Whereas the antifungal activity of the triazole-based azoles was dependent on expression of CYP51, the target of the azole antifungals, imidazole-based compounds displayed antifungal activity against a mutant lacking CYP51, indicating that imidazole-based azole antifungals have additional modes of action. This peculiarity could be favorable in the struggle against drugs-resistant fungal strains.

**Figure 25:**
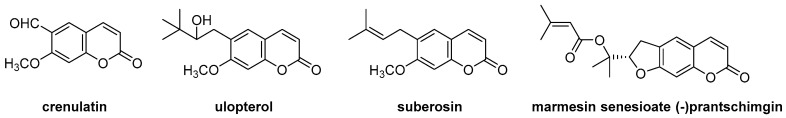
Coumarins from *Ferulago trachycarpa*.

Furthermore, it should not be forgotten the contribution of natural coumarins to the fight against *Candida* infections. *Ferulago trachycarpa* Boiss. is one of the species of *Ferulago* W. Koch., common in Anatolian region, exploited in traditional medicine but also in culinary field. Previous studies showed that coumarins are the main compounds found in *Ferulago* but only in 2018, Dikpinar and colleagues conducted the first antimicrobial activity guided isolation of the molecular constituent of this particular species of *Ferulago* [97]. Four coumarin compounds (Figure 25) were isolated and purified and then three of them were tested against bacterial and fungal strains; in particular, marmesin senesioate, suberosin and crenulatin showed antifungal activity with 625 mg/L MIC against *Candida albicans*. 

The antifungal potency of coumarin itself against *Candida albicans* had been previously evaluated, in particular its antibiofilm activity [98,99]. Recently, Xu and co-workers focused their attention on the possible way to prevent the adhesion and formation of biofilm by *Candida albicans* on implanted medical devices. The research group observed that coumarin not only suppresses biofilm formation but also affects the structure of the mature biofilm; moreover the adhesion, the cell surface hydrophobicity (CSH) and the filamentous growth of *C. albicans* significantly decreased after coumarin treatment, indicating that coumarin inhibits biofilm formation by suppressing adhesion [100]. 

**Figure 26:**
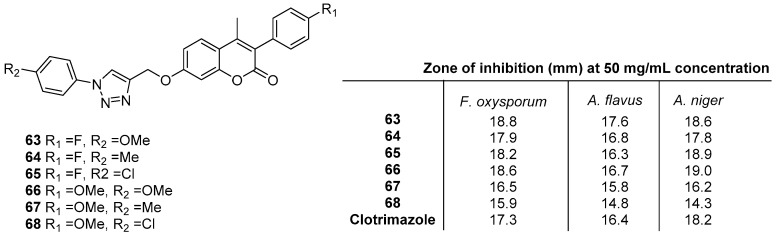
Coumarin-based 1,4-disubstituted 1,2,3-triazole derivatives.

The antifungal potency of coumarin-based triazoles against other fungal strains in addition to *Candida* species had been evaluated by Dharavath and colleagues in 2020. Coumarin-based 1,4-disubstituted 1,2,3-triazole derivatives were synthesized using a highly efficient, eco-friendly protocol via a copper(I)-catalyzed click reaction between various substituted arylazides and terminal alkynes. The in vitro antifungal activity was tested against *Aspergillus niger*, *Aspergillus flavus* and *Fusarium oxysporum* by using the disc diffusion method and the results were compared with those of clotrimazole, the reference drug. Compounds **63**–**68** (Figure 26), characterized by the presence of a *para*-substituted phenyl moiety, directly linked to the triazole ring, showed comparable activity in respect to the reference compound clotrimazol [101,102]. 

### 2.6. Antiviral Activity

2020 has been a crucial year for the timeless war between human and viruses: World Health Organization (WHO) declared the outbreak of Sars-COVID-19 a Public Health Emergency of International Concern on 30 January 2020 and on 11 March WHO characterized COVID-19 as a pandemic [103]. Whole developed countries have been quarantined and generations that never faced medical crisis are now struggling with the consequences of the viral diffusion. Human history is afflicted by the cyclic succession of pandemic events and the research of new antivirals is still ongoing, due to the ability of viruses to mutate and the continuous appearance of new viruses on the medical scenario [104].

The natural world is a priceless source of valuable medical compounds and also in the fight against viral diseases there are several natural molecules which exhibit antiviral activity [105,106,107,108,109]. Coumarins, likewise other polyphenolic compounds, exert a remarkable antiviral activity [110,111]. The antiviral activity of coumarins explicates through different mechanisms which affect the life cycle of viruses and their biological activities could be changed depending upon the combination of various substituents and conjugates [104,112]. Coumarins appears to be active against several viruses, like HIV, influenza, hepatitis, Dengue and Chikunguya [104].

Liu and co-workers after a phytochemical study on the stem of *Clausena lenis* isolated three new and nine known prenylated coumarins [113]. All the prenylated coumarins were evaluated both for their anti-inflammatory and anti-HIV reverse transcriptase (RT) activities. In this last case, the inhibition assay for the cytopathic activities of HIV-1 (EC_50_) as well as cytotoxic activity assay against C8166 cell line (CC_50_) according to MTT methods were applied [114,115].

**Figure 27:**
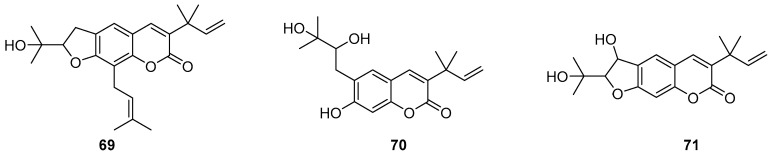
New prenylated coumarins from *Clausena lenis*.

The three new isolated compounds (**69**–**71**, Figure 27) showed the best inhibitory activity with an EC_50_ of 0.29, 0.68 and 0.17 μM, respectively. Furthermore, no cytotoxicity was observed against C8166 cell line (CC_50_ > 200.00 μM).

**Figure 28:**
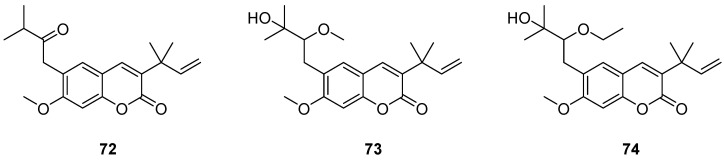
Prenylated coumarins from *Manilkara zapota*.

Prenylated coumarins came to the fore in 2019 thanks to a work by Liu et al., who revealed for the first time the presence of these type of derivatives in the fruits of *Manilkara zapota*, an ever-green tropical tree. Also in this case, three new derivatives were identified (**72**–**74**, Figure 28) together with seven known compounds and the team evaluated their anti-inflammatory and anti-HIV activities, exploiting the methods described above [116]. 

The new prenylated coumarins showed the highest anti-HIV RT effect among the prenylated coumarins derived from the fruit of *Manilkara zapota*; in particular, compound **72** displayed the most powerful effect with an EC_50_ value of 0.12 µM. Comparative studies between compounds **72**–**74** and the other coumarin derivatives isolated from the fruits highlighted the importance of the isopentenyl group as a substituent at C6 and 2-methylbut-3-en-2-yl group as a substituent at C3 for the anti-HIV RT effect. 

In the same year, Jesumoroti and co-workers approached the problem from another point of view, both regarding the target and the origin of the coumarin derivatives [117]. First of all, a different viral target was chosen, HIV-1 IN, which is essential for a stable infection. Moreover, there are no homologous enzymes in the host cell [118]. Secondarily, the coumarin derivatives were obtained decorating the coumarin nucleus with an hydrazide group, in order to achieve a synergistic effect against-HIV-1-IN and reducing the toxicity correlated to the salicylhydrazide, which was however essential for the activity [119,120,121]. The synthesized compounds were evaluated for their in vitro HIV-1 IN inhibitory activity using chicoric acid (CA) as a reference compound, according to the method described by McColl et al. [122]. 

**Figure 29:**
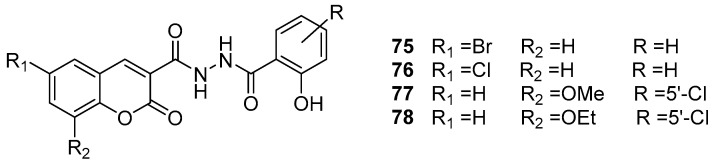
Coumarin derivatives synthesized by Jesumoroti and co-workers [117].

Compounds **75**–**78** (Figure 29) appeared to be the most active among the whole series showing an inhibition of extracellular IN (evaluated in vitro according to the method described by McColl et al. [122]) in a range from 95% to 86 % and IC_50_ between 13 nM (compound **76**) and 31 nM (CA IC_50_ = 10 nM). Furthermore, the cytotoxicity of all the obtained compounds was tested and derivatives **75**–**78** showed low or negligible cytotoxicity.

**Figure 30:**
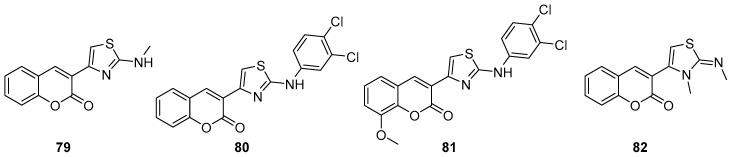
Hybrid pharmacophores proposed by Osman and co-workers [125].

Seasonal influenza claims around 0.29–0.65 million victims annually, even if several drugs are commercially available [123]. The flu is a contagious respiratory disease caused by influenza viruses, manifesting major epidemics with no predictable periodicity or pattern and all different from one to another [124]. For these reasons, a constant effort in the development of new drugs for the treatment of this disease is of greatest importance. In 2019 Osman and co-workers combined two bioactive moieties into a single molecule in order to obtain new bioactive compounds with antibacterial and antiviral effects [125]. In particular, based on a previous study of the same research team, in which coumarin scaffolds and thiazole moiety were combined leading to compounds endowed with both antibacterial and antiviral activities, the potentiality of this combination was further explored [126], [127]. Four new molecules of the series showed a remarkable antiviral activity against H1N1 virus. Compounds **79**–**82** (Figure 30) seemed to be promising agents, having IC_50_ values of 4.84, 19.72, 6.12 and 9.13 μg/mL, respectively, against the H1N1 virus.

**Figure 31:**
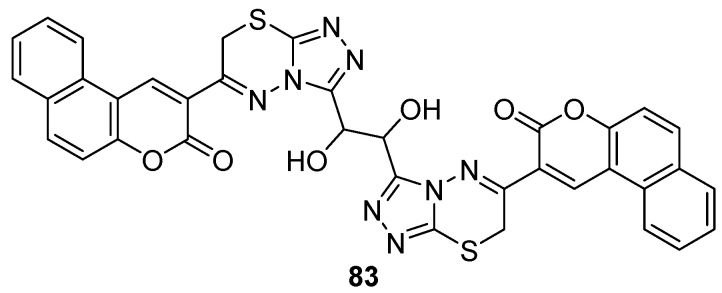
Bis coumarinyl bis triazolothiadiazinyl ethane derivative.

A similar approach was followed by Pavurala et al., in 2019, who exploited the versatility of the coumarin scaffold by combining it with another bioactive moiety, in this case a triazolothiadiazine [128]. The result was the synthesis of a series of bis-coumarinyl-bis-triazolothiadiazinyl ethane derivatives that were evaluated for their antiviral activities against different DNA and RNA viruses. Among the tested compound **83** (Figure 31) exhibited a good antiviral activity against influenza A virus. More specifically, in cytopathic effect (CPE) reduction assay against influenza virus infected Madin–Darby canine kidney (MDCK) cells, it displayed antiviral EC_50_ values of 20–72 μM. 

**Figure 32:**
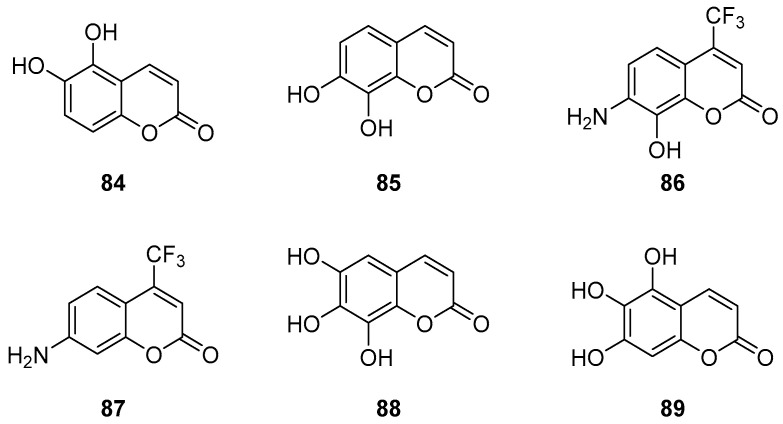
Antiviral coumarins from Bizzarri et al. [129].

Bizzarri et al. exploited the regioselective oxidation of coumarin derivatives with 2-iodoxybenzoic acid (IBX) in order to obtain catechol and pyrogallol moieties [129]. After cytotoxicity studies that confirmed the safety of the series of derivatives, the antiviral activity against influenza viruses A/PR8/H1N1 was evaluated and compounds **84**–**89** (Figure 32) resulted able to inhibit the viral replication. Interestingly, pyrogallol derivatives **88** (IC_50_ = 69.9 μg/mL) and **89** (IC_50_ = 47.9 μg/mL) turned out to be more active than catechol derivatives **84** (IC_50_ = 106.5 μg/mL) and **85** (IC_50_ = 91.5 μg/mL). Moreover, pyrogallol and catechol derivatives were more active than the monohydroxycoumarins 6-hydroxycoumarin and 7-hydroxycoumarin (IC_50_ 110 μg/mL for both the parent compounds). 

One of the possible advantages of oxidized coumarins could be their mode of action against viruses. Indeed, due to their antioxidant activity, coumarins derivatives could affect intracellular redox-sensitive pathways useful for viral replication, independently from the variability of the strains [129].

**Figure 33:**
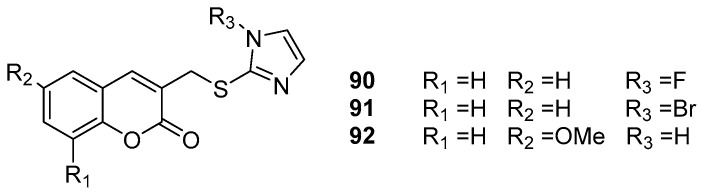
Anti-hepatitis coumarins.

As already mentioned, coumarins have been studied also for their potential application as anti-hepatitis agents. Tsay and co-workers studied the activity against hepatitis C virus (HCV) of some unnatural imidazole-coumarin conjugates [130]. Above all, three compounds (**90**–**92**, Figure 33) showed a noteworthy antiviral activity against HCV with EC_50_ values ranging from 5.1 to 8.4 μM and selective indices (SI = CC_50_/EC_50_, which is a measure for the therapeutic window of the compound in an assay system) higher than 20. 

Huang and co-workers focused their attention on the investigation of the potentiality expressed by esculetin (or 6,7-dihydroxycoumarin) against hepatitis B virus (HBV) [131]. The results suggested that esculetin efficiently inhibits HBV replication both in vitro and in vivo, which provides an opportunity for further development of the compound as antiviral agent.

### 2.7. Anti-Inflammatory Activity

Inflammation is a general physiological response that aims, firstly, to the circumscription of the harmful factors, which could be both endogenous (e.g., cancer, ischemia, autoimmune diseases) and exogenous (e.g., viral or bacterial infections, trauma), secondarily, to the removal of the causes of the damage and finally to the reparation of the tissues and restoration of the functions. Nevertheless, the inflammation and the consequent restorative process could become harmful for the organism itself when there is a persistent stimulation and the phases of inflammation and reconstruction are contemporary activated, causing tissue injuries and fibrosis [132]. During an inflammatory process, many inflammatory effectors and mediators are produced and involved, often with a common effect on vascular system and on the recruitment of leukocytes [133]. Frequently, inflammatory mediators are the target of anti-inflammatory drugs [134,135,136,137]. 

**Figure 34:**
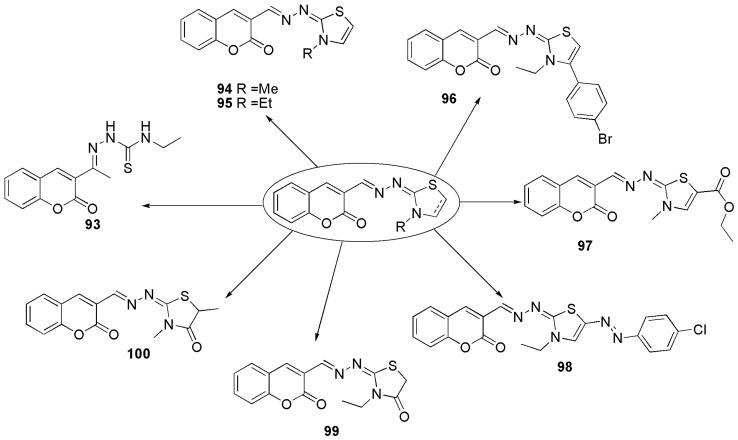
Anti-inflammatory coumarin derivatives.

Among the numerous biological activities shown by coumarin derivatives, the anti-inflammatory effect could not certainly miss. Dawood et al., in 2015, developed a new series of coumarin derivatives hybridizing two pharmacophoric moieties—the coumarin scaffold itself and thiazoline or thiazolidinone groups, both showing cyclooxygenase 2 (COX-2) inhibitor effect [127], [138,139,140,141,142,143]. The compounds were evaluated in vivo for their systemic effect, in vitro for their ability to inhibit human COX-1 and COX-2 and also to evaluate the ulcerogenic potential compared to the reference drug, indomethacin, always following standard methods reported in literature. Most of the new compounds showed significant in vivo anti-inflammatory activity with a superior gastro-intestinal safety profiles (0–7% ulceration) as compared to indomethacin. The IC_50_ values of all the bioactive compounds ranged between 0.31 and 0.78 mM, showing an in vitro high affinity and selectivity toward the COX-2 isoenzyme, compared to the reference celecoxib. Ethyl thiosemicarbazone **93**, thiazoline derivatives **94**–**98** and the thiazolidinone compounds **99**–**100** exhibited the highest in vivo and in vitro anti-inflammatory activities with good COX-2 selectivity (Figure 34) [143]. 

Nevertheless, cyclooxygenases are not the only enzymes involved in the inflammatory process. Actually, 5-lipoxygenase (5-LOX) catalyzes two different steps in the arachidonic acid metabolism that brings to the production of leukotriene A_4_, which is successively metabolized into leukotriene B_4_ [144]. Molecular inhibitors of leukotrienes competitively bind the active site of 5-LOX and are divided in three category: redox-active compounds (i.e., coumarins), iron-ligand inhibitors with weak redox-active properties and non-redox-type inhibitors [145]. 

**Figure 35:**
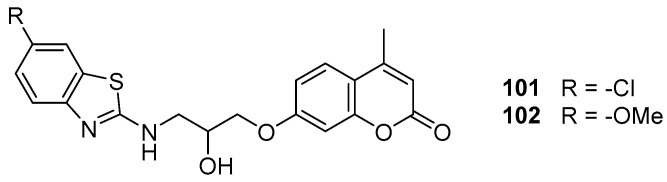
Anti-inflammatory 7-substituted coumarins proposed by Srivastava [146].

In 2016, Srivastava and colleagues evaluated the anti-inflammatory and analgesic effect of a series of synthesized 7-substituted coumarins and, consequently, the most active compounds were assessed in vitro for 5-LOX inhibition [146]. Compounds **101** and **102** (Figure 35) resulted the most promising derivatives, also in the ulcerogenic risk evaluation when compared to acetylsalicylic acid. In vitro kinetic study of compound **102** (IC_50_ = 2.09 nM) showed mixed or non-competitive type of inhibition of 5-LOX. The presence of a substituent on C6 position of benzothiazole ring was found very important for increasing the activity. 

**Figure 36:**
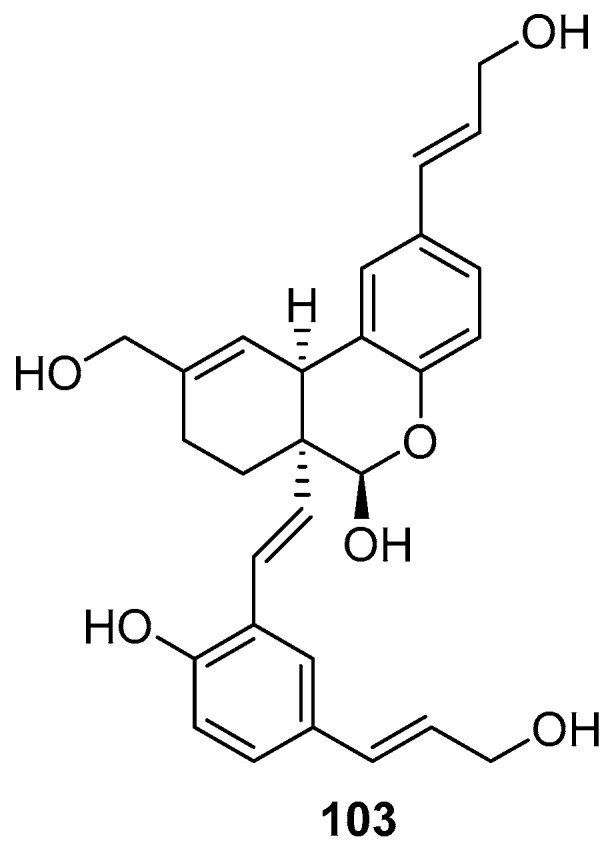
One of the coumarin derivatives isolated from *M. exotica.*

In 2018, Liu et al. identified ten new coumarin derivatives (3 monomers and 7 dimers) isolated in a phytochemical study on *Murraya exotica*, a dwarf tree that is native of the tropical and subtropical areas of Asia and traditionally used in the treatment of inflammatory-related diseases [147]. Previous studies had shown that the main active components in *M. exotica* were coumarins; also bis-coumarins were isolated from the plant. A further investigation on the 95% aqueous EtOH extract of the roots allowed the obtainment of new coumarins, together with other bioactive molecules. The compounds were tested for their inhibitory effect on NO production and compound **103** (Figure 36) stood out among the coumarins in the inhibition against LPS-induced NO production in BV-2 microglial cells with IC_50_ value of 8.6 μM.

**Figure 37:**
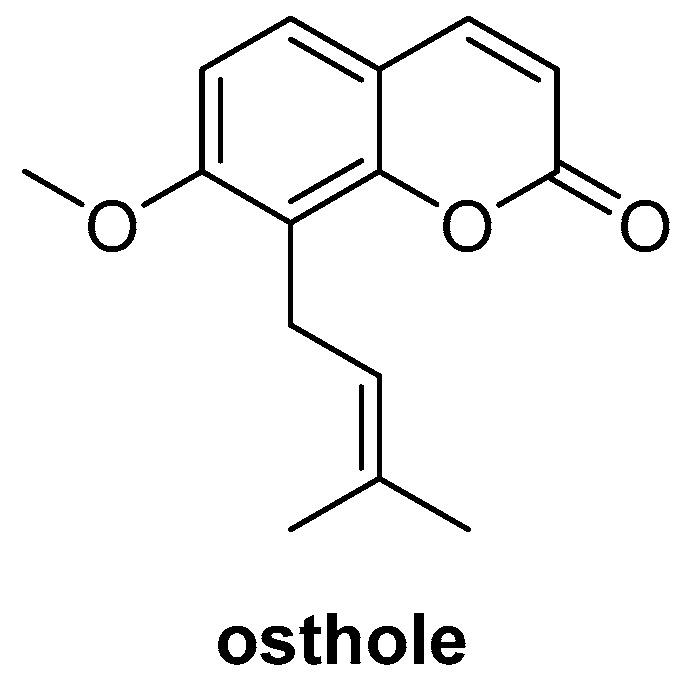
Structure of the natural coumarin osthole.

The nuclear factor-kB (NF-kB) family of transcription factors is composed by a set of related, evolutionarily conserved DNA-binding proteins. In response to multiple stimuli such as inflammatory cytokines, bacterial lipopolysaccharide (LPS), viral infection or stress, a series of cascade reactions bring towards the entry on NF-kB into the nucleus and to the activation of several genes participating in immune and inflammatory responses, cell adhesion, growth control and regulation of apoptosis [148,149]. In 2019, Fan and co-workers evaluated in vivo and in vitro the anti-inflammatory activity of osthole (Figure 37) [150], a natural prenylated coumarin from *Cnidium Monnieri* (L.) Cuss. but also found in other medicinal plants, which has already shown different biological and pharmacological properties such as neuroprotective, osteogenic, immunomodulatory, anticancer, hepatoprotective, cardiovascular protective and antimicrobial activities [13]. The team established that osthole inhibited the production of NO, PGE2, TNF-a and IL-6 in LPS-induced macrophages and suppressed the activity of iNOS and COX-2, probably by blocking the activation of NF-kB and p38/MAPK signaling pathways. 

**Figure 38:**
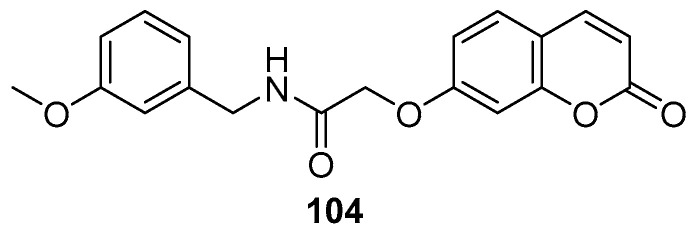
7-substituted coumarin as blockers of NF-κB signaling pathway.

In the same year, Mu and colleagues synthesized a series of eleven 7-substituted coumarins and evaluated their anti-inflammatory activities and their ability to exploit the NF-kB pathway [151]. All the screened compounds showed a relevant anti-inflammatory activity. In the series, compound **104** (Figure 38) was identified as a hit and further studies of molecular docking were conducted confirming that **104** could bind to the active site (NLS Polypeptide) of NF-κB p65. Its binding affinity was further confirmed by surface plasmon resonance (SPR) analysis. Moreover, Western blot assay showed that **104** remarkably blocked the NF-κB signaling pathway in vitro. 

### 2.8. Neuroprotection: Effect on Alzheimer’s Disease

Alzheimer’s disease (AD) is the most common form of dementia (60–70% of cases of dementia are cause by AD) and consists in a neurodegenerative disorder characterized by a slow, progressive and irreversible loss of cognitive function and memory [152,153,154,155,156,157]. The current therapeutic approach, mainly based on the use of acetylcholinesterase (AChE) inhibitors (rivastigmine, donepezil, galantamine) or *N*-methy-D-aspartic acid (NMDA) receptor inhibitors (memantine), is merely symptomatic and does not counteract degeneration progression. New innovative approaches, such as multi-targeted strategies, are urgently required in order to cure cognition and motor dysfunctions, neurodegeneration and depression. Among their biological activities, coumarins showed the ability to inhibit some biological targets involved in AD. With this in mind, some recent works aimed at investigating coumarins potential in the treatment of AD are discussed below. 

**Figure 39:**
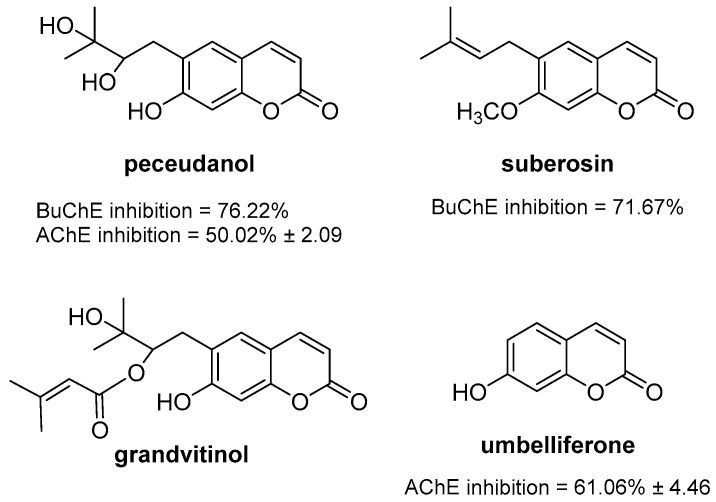
Coumarins extracted from *Ferulago cassia*.

In 2019, Karakaya and collaborators investigated the antioxidant and AChE/BuChE inhibitory activity of aerial parts, fruits, flowers and root extracts from *Ferulago cassia* Boiss [158]. Root’s and fruit’s dichloromethane extracts showed the highest antioxidant potential in TBA assay. The evaluation of anticholinesterase activity was carried out by means of the Ellman’s method [159]: dichloromethane extracts showed significant inhibition against BuChE (96.56% ± 2.98 and 82.33% ± 2.69, respectively) at 20 μg/mL and appreciable inhibition against AChE (53.24 ± 1.22 and 31.38 ± 5.41%, respectively) at 20 μg/mL. Four coumarins were isolated from *Ferulago cassia*—peucedanol, suberosin, grandivitinol and umbelliferone (Figure 39). Therefore, *F. cassia* can be considered a starting point for the development of new compounds with antioxidant and anticholinesterase activity. 

**Figure 40:**
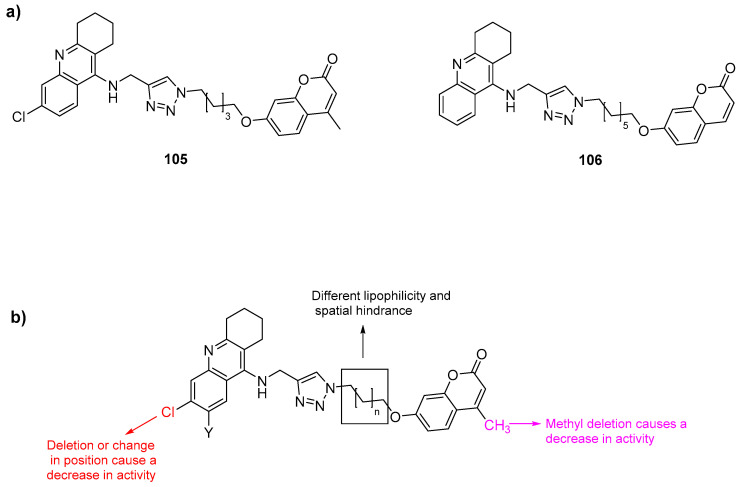
(**a**) Chemical structure of compounds **105** and **106**; panel (**b**) SAR studies.

Thanks to their simple structural architecture and chemical stability, coumarins can be easily synthesized and modified in order to produce more active and selective compounds. Najafi and co-workers synthesized a series of tacrine-coumarin derivatives linked to a 1,2,3-triazole moiety and evaluated their activity in terms of AChE and butyrylcholinesterase (BuChE) inhibition, keeping donepezil and tacrine as reference drugs [160]. In addition, their beta-secretase 1 (BACE1) inhibitory activity and neuroprotective potential were evaluated. Since tacrine is a well-known inhibitor of the catalytic site of AChE, whereas coumarins showed affinity for the peripheral anionic site (PAS) [161], this new compounds may be potential dual—and therefore more powerful - inhibitors of ChEs. The in vitro AChE and BuChE inhibitory activity was evaluated using the Ellman’s method [159]; among all the tested molecules, compound **105** resulted the best in AChE inhibition (IC_50_ = 0.027 ± 0.009 μM; tacrine IC_50_ = 0.048 ± 0.011 μM, donepezil IC_50_ = 0.039 ± 0.097 μM) and compound **106** was the most promising BuChE inhibitor (IC_50_ = 0.006 ± 0.002 μM; tacrine IC_50_ = 0.010 ± 0.004 μM, donepezil IC_50_ = 8.416 ± 0.628 μM) (Figure 40).

**Figure 41:**
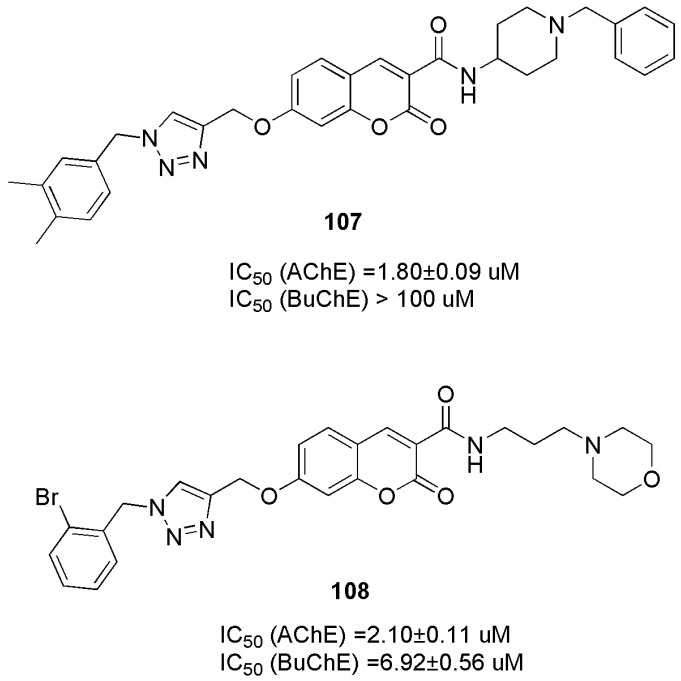
Chemical structure and IC_50_ values of compounds **107** and **108**.

Concerning the anti-BuChE activity, structure-activity relationship studies showed that Cl and Me substituents, as well as the methylene linker, play a complex and not completely understood role in the enzyme inhibition. From the evaluation of inhibitory activity of the synthesized compounds on BACE1, a moderate inhibitory activity of compound **105** was observed (inhibition of 28.69% and 13.97% at 50 and 10 μM, respectively). Nevertheless, compound **105** did not show neuroprotective action on PC12 cells exposed to Aβ_25-35_. Then, in vivo evaluation of compound **105** using the Morrison Water Maze method [162] was made and valuable results based on memory improvement in scopolamine-induced impairment were observed. Similarly, Rastegari and collaborators synthesized a series of 1,2,3-triazole-chromenone carboxamide derivatives and investigated their potential as anti-Alzheimer’s agents, in terms of inhibitory activity against AChE, BuChE and BACE1, besides their neuroprotective and metal-chelating properties [163]. The anti-AChE activity of coumarin-3-carboxamide was already known [164], as well as the ability of the 1,2,3-triazole moiety to enhance AChEI activity, if linked with tacrine or phenanthridinium derivatives and its BACE1 inhibitory potential [165]. In vitro AChEI and BuChEI activities of the new compounds were evaluated, keeping donepezil as reference. Higher activities were shown by compound **107**, bearing 3,4-dimethylbenzyl connected to 1,2,3-triazole moiety and by compound **108**, having 3-morpholinopropyl and 2-bromobenzyl moieties (Figure 41), even if they are much less active than donepezil (IC_50_ = 0.027 μM). Anti-BChE activity was also modest and resulted to be affected by the type of amine connected to the amide moiety, that is, morpholine or piperidine and by the position and electronic properties of substituents on the benzyl group connected to 1,2,3-triazole ring. 

Compound **107** was chosen for BACE1 inhibitory activity evaluation, showing only modest results (IC50 = 21.13 μM compared to the reference OM99-2 having IC50 = 0.014 μM). Also, neuroprotective potential of **107** was investigated by comparing **107**-treated cells with intact (no intervention), quercetin+H_2_O_2_-treated (positive control) and H_2_O_2_-treated (negative control) cells. The mentioned compound showed moderate to good neuroprotective activity, not stronger than quercetin. Finally, since ROS, which are potentially involved in the neurodegenerative process of AD, may be generated from unregulated reaction of molecular oxygen with the redox active metals such as Fe, Cu and Zn, the metal-chelating properties of **107** toward Cu^2+,^ Fe^2+^ and Zn^2+^ were tested in methanol by means of UV-vis spectrophotometry. Interaction between **107** and Cu^2+^ and Zn^2+^ was detected, whereas no interaction was observed between **107** and Fe^2+^.

**Figure 42:**
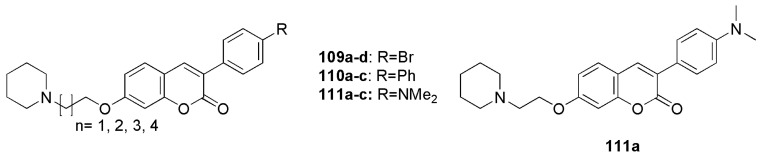
Chemical structures of alkylamino coumarins **109a**–**d**, **110**a–**c** and **111a**–**c**.

De Souza and co-workers designed and synthesized a series of 3-substituted-7-aminoalcoxy-coumarin derivatives (**109a**–**d**, **110a**–**c**, **111a**–**c**, Figure 42) whose AChEI/BuChEI activities and antioxidant properties were evaluated [166]. All the compounds showed good inhibitory activity on AChE, with potencies in the nanomolar range, whereas their activity against BuChE was lower (IC_50_ between 0.90 to 15.85 μM); 3-(4-(dimethylamino)phenyl)-7-aminoethoxycoumarin (**111a**) was considered a hit, showing an excellent inhibitory activity (IC_50_ = 20 nM) and selectivity towards AChE (IC_50_ BuChE/AChE = 354) compared to the reference drug donepezil (IC_50_ = 6 nM, IC_50_ BuChE/AChE = 365). Investigation of antioxidant properties showed that only compounds **111a**–**c** presented activity in Ferric Ion Reduction Method (FRAP), whereas series **109a**–**d** and **110a**–**c** did not show significant results, suggesting that the dimethylaminophenyl moiety may be responsible for the antioxidant activity. 

Docking simulations showed that all the compounds were able to bind simultaneously the PAS and cationic active site (CAS) of the electric eel AChE (*ee*AChE): the interaction with the CAS took place by means of cation-π interaction between piperidinyl group and Trp86. Compounds **111a** and **111c** showed the ability to give a π-stacking interaction in the PAS, with Trp286. The smaller spacer of **111a** allowed the coumarin moiety to be located properly in the gorge, achieving H-bonds with Tyr337 and Phe295. This is probably the reason for the most efficient binding mode (and consequently the best activity) to *ee*AChE of compounds with a short spacer. 

**Figure 43:**
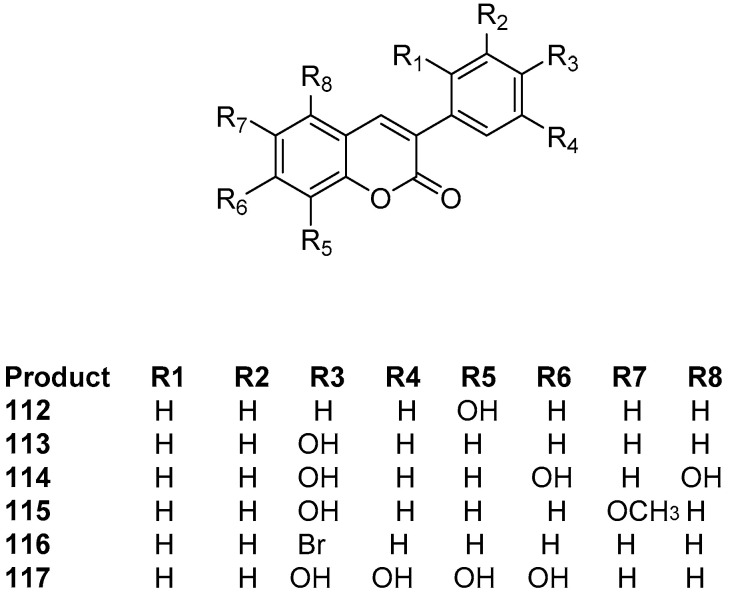
General structure of compounds **112**–**117**.

Yang and his group synthesized a series of twenty-four 3-aryl coumarin derivatives and tested their cholinesterase inhibitory activity, monoamine oxidase (MAO) inhibitory activity and antioxidant activity in vitro [167]. As far as cholinesterase inhibition is concerned, most of the mentioned compounds showed moderate activity. Compound **117** (Figure 43) exhibited a strong activity (IC_50_ = 3.04 ± 0.32 μM), whereas compound **116** showed selectivity towards AChE. Compound **114** was more active against BuChE (IC_50_ = 2.76 ± 0.57 μM) than donepezil, whereas compounds **112**, **113** and **115** showed selectivity towards BuChE. 3-Arylcoumarins bearing hydroxy group both at R_5_ and R_6_ positions displayed higher activity respect to the mono-substituted counterparts, especially towards AChE, whereas no significant impact on BuChE inhibition was observed. 

As mentioned, MAO-inhibitory activity was evaluated as well. In fact, MAO is one of the enzymes responsible for oxidative stress, which is another factor involved in the neurodegenerative process characterizing AD and different studies identified various ChE/MAO inhibitors as tools for AD treatment, showing positive results in clinical trials [168]. In Yang’s work, 3-arylcoumarins anti-MAO activity was compared to that of rasagiline (a selective MAO-B inhibitor): almost half of the compounds showed relevant activity [167]. Among them, compound **117** was the most promising, with an IC_50_ = 27.03 ± 0.50 μM, though weaker than rasagiline (IC_50_ = 0.125 ± 0.005 μM). Again, the presence of R_5_, R_6_ hydroxy groups provided better MAO inhibitory activity. Finally, the antioxidant power of 3-arylcoumarins was studied by means of Ferric Ion Reduction Method (FRAP), using vitamin C as a reference. Compound **117** resulted again the most active (FRAP value = 41.42 ± 0.35), also showing a certain activity in vivo, when tested on zebrafish, leading to an increase of total distance of zebrafish movement proportional to the concentration of compound used.

**Figure 44:**
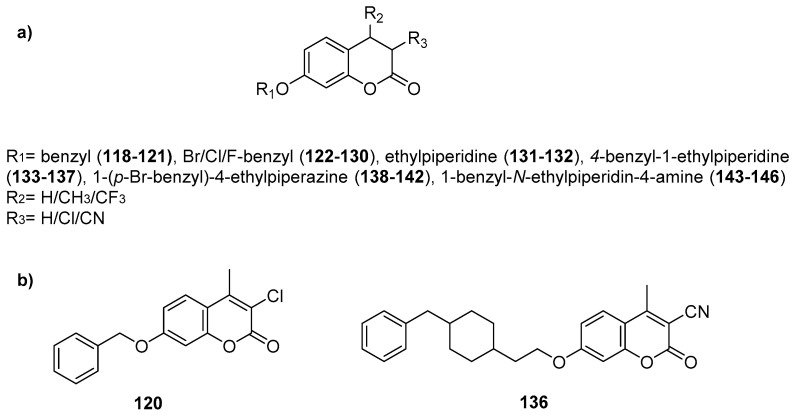
(**a**) General structure of compounds **118**–**146**; (**b**) chemical structure of compounds **120** and **136**.

In 2017, Joubert and collaborators designed and synthesized a series of 7-substituted coumarins (**118**–**146**, Figure 44), consisting in a coumarin motif (MAO inhibitor) condensed with a benzyl-, piperidinyl-, *N*-benzylpiperidinyl- or *p*-bromo-*N*-benzylpiperizinyl moiety, which resemble the *N*-benzylpiperidine function present in donepezil (AChE inhibitor), connected via an alkyl ether linkage at position 7 [169]. Their biological activities were evaluated in terms of MAO and ChE inhibitory potential. Inhibition of *h*MAO was achieved from all the designed compounds, which also showed selectivity towards MAO-B. The benzyloxy series showed higher activity, in some cases even better than the reference selegiline (IC_50_ = 0.008 μM), with IC_50_ values in the nanomolar range (0.5–73 nM).

**Figure 45:**
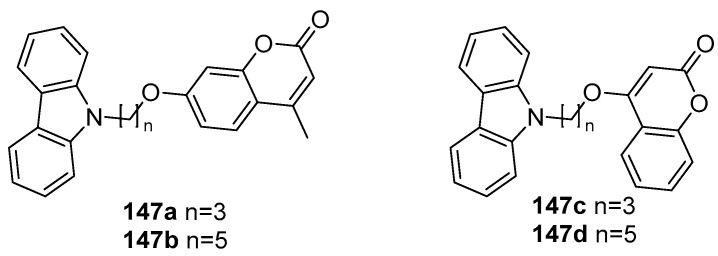
Chemical structure of compounds **147a**–**d**.

The presence of piperidine or piperazine ring caused a decrease in MAO inhibitory activity, perhaps indicating that larger substitutions at position 7 are not well tolerated. Again, no activity against MAO-A was shown, whereas IC_50_ values between 0.29 and 5.64 μM were detected on MAO-B. Compounds that lacked the *N*-benzylpiperidine, responsible for anti-cholinesterase activity in donepezil, showed little or no activity against *ee*AChE and equine BuChE (*eq*BuChE). Compounds bearing the *N* -benzylpiperidin moiety showed moderate inhibitory activity against both ChEs (IC_50_ values between 1.42 and 10.22 μM) with some slight selectivity towards BuChE. Compounds **120** and **136** (Figure 44b) resulted the most promising. In fact, molecular modelling revealed that compound **120** can realize favorable interactions with the active sites of both BuChE and MAO-B. This double activity may be advantageous in AD treatment, because both enzymes are becoming relevant biological targets for the treatment of this dementia. Also compound **136** resulted to be a potential MTDL because it showed inhibitory activity against *h*MAO-B (IC_50_ = 0.30 μM), *ee*AChE (IC_50_ = 9.10 μM) and *eq*BuChE (IC_50_ = 5.90 μM). Docking studies showed that **136** binds the substrate cavity through the coumarin ring, whereas the benzyl moiety occupies the entrance cavity. In addition, **136** was able to bind both the CAS and PAS of cholinesterase, which suggested that it could interfere with PAS-induced β-amyloid aggregation. A different approach was followed by Shi and collaborators, who designed four derivatives obtained by coupling two pharmacophores (carbazole and coumarin) to obtain potential multitarget agents for the treatment of AD [170]. The aim was to exploit the biological activities of both the mentioned moieties: on one hand coumarins are known to have inhibitory activity on AChE, BuChE and MAO, besides antioxidant properties; on the other hand, carbazole exhibits antioxidant activity [171] and the ability to inhibit Aβ aggregation [172]. In addition, carbazole derivatives are reported to have inhibitory effect on ChEs, thus providing neuroprotection from β-amyloid toxicity [173]. Thus, compounds **147a–d** (Figure 45) were synthesized, characterized and their biological activities were evaluated: anticholinesterase activity was tested on *ee*AChE and on *h*AChE, using Ellman’s method [159], keeping tacrine as reference drug. Whereas all derivatives showed dose-dependent inhibitory activity on *ee*AChE (compound **147d** resulted the best with an IC_50_ of 3.75 μM), on *h*AChE they exhibited weak effects (**147b** IC_50_ = 76.63 μM, **147d** IC_50_ = 70.51 μM). None of the tested compounds inhibited *eq*BuChE or *h*BuChE more than 30%, which means that compounds **147b** and **147d** showed selectivity for AChE over BuChE. 

As the alkyl linker length increased, the anti-cholinesterase activity improved, probably because a 5-methylene chain allowed the carbazole and coumarin moieties to bind both the CAS and the PAS, respectively. Antioxidant properties were evaluated through the ORAC-FL method (oxygen radical absorbance capacity by fluorescein) [174], using Trolox (vitamin E analogue) as a standard; none of the tested compounds showed significant activity. Among the tested derivatives, compound **147d** resulted a promising lead candidate for the treatment of AD. 

### 2.9. Anticonvulsant Activity

**Figure 46:**
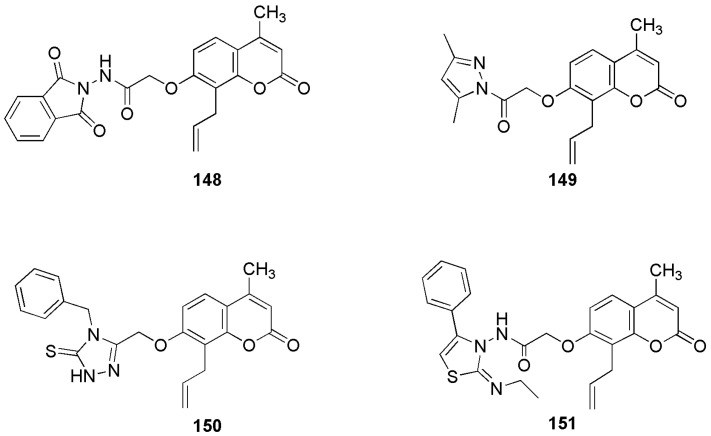
Chemical structure of compounds **148**–**151**.

Epilepsy is a widespread neurological disorder, characterized by periodic and unpredictable attacks, involving seizures and/or transient behavioral changes. Its pathogenesis has not been completely clarified yet; it is known, however, that an impairment between excitatory and inhibitory neurotransmission is involved [175,176,177,178,179] Here, we report some recent advances in the use of coumarins as anticonvulsant compounds. Abd-Allah and collaborators recently studied the anticonvulsant activity of a series of coumarin derivatives, achieved by merging two or more pharmacophoric scaffolds in order to create new chemical entities with an improved biological activity [180]. The compounds here described possess all the necessary elements to exert anti-convulsant activity: a lipophilic aryl ring, a hydrogen-bonding domain and an electron-donor moiety [178,179,181,182]. All the compounds were initially screened (phase I) using two standard animal seizure models, subcutaneous pentylenetetrazole (scPTZ) and maximal electric shock (MES) seizure tests using ethosuximide as reference drug. An assessment of the potential neurotoxicity was also done by means of rotarod test. Phase II consisted in the determination of ED_50_ value for compounds that conferred 100% protection in one or both tests. In the end, GABA level measurements were carried out in whole mouse brain for the most active compounds, using gabapentin as a reference drug. The results of phase I tests showed that all the tested compounds had protective activity against scPTZ-induced absence epilepsy (variable results in the range of 17–100% protection). Among them, **148**, **149** and **150** (Figure 46) were the most active (100% protection) at 0.238, 0.239 and 0.283 mmol/kg, meaning that the compounds are 1.49, 1.48, 1.25 folds more potent than ethosuximide, respectively. In the MES-induced seizures though, all the compounds failed in completely protecting the animals. The best profile was exhibited by compound **151** (Figure 46) which was capable of a 50% protection at 2.1 mmol/kg. A quantitative determination of ED_50_ values was carried out for compounds **148**, **149** and **150**, which were able to fully protect animals in the scPTZ test. Compound **148** was found to be the most active with an ED_50_ of 54.86 mg/kg (0.131 mmol/kg). Consequently, it was chosen for further investigation to elucidate the mechanism of action, which was assessed through an evaluation of GABA levels in mice brain. As a result, the proposed mechanism for compound **148** is a GABA-mediated one, perhaps non-vesicular release of GABA, GABA_A_ receptor activation or GABA_B_ receptor inhibition; probably an enhanced synthesis or reduced metabolism of GABA are also involved.

**Figure 47:**
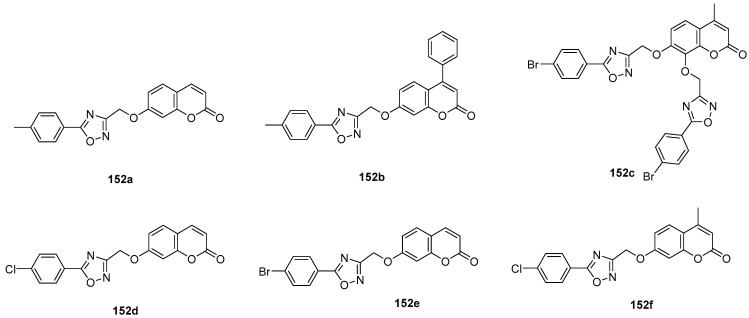
Chemical structure of compounds **152a**–**f**.

A similar bivalent drug approach was followed by Mohammadi-Khanaposhtani and collaborators, who synthesized a series of coumarin-1,2,4-oxadiazole derivatives in order to create a new chemical entity with better anticonvulsant profile than coumarin and oxadiazole alone [183]. In fact, different 5-member heterocyclic rings-containing compounds such as oxadiazoles, triazoles and thiadiazoles were reported to have good anticonvulsant activity [184,185,186] through benzodiazepine (BDZ) receptor [187]. The activity of the new derivatives was tested using PTZ- and MES- induced seizures in mice, keeping diazepam as a reference drug. Most of the new compounds did not show activity against PTZ-induced seizures, except for three of them, **152a**, **152b** and **152c** (Figure 47), being **152b** the most active (25% of protection using 10 mg/kg). Compounds **152d**, **152e** and **152f** (Figure 47) showed a 100% protection against MES-induced seizures at the doses of 7, 40 and 20 mg/kg, respectively (it should be considered that diazepam shows 100% protection at 2 mg/mL) [188]. Compound **152d**, which showed the best activity, was characterized by the absence of substituents on position 4 of the coumarin ring and by a 4-chloroaryl group connected to the 1, 2, 4-oxadiazole ring. The most active compounds **152d** and **152e** were used to investigate the mechanism of action; to do so, the effect of flumazenil (a BDZ receptor antagonist) on their activity was evaluated. Flumazenil antagonized both **152d** and **152e**, confirming that these compounds act as BZS receptor agonists. Finally, in vivo neurotoxicity of compounds **152d** and **152e** was assessed and the tested compounds gave less neurological deficits that the reference drug diazepam. 

**Figure 48:**
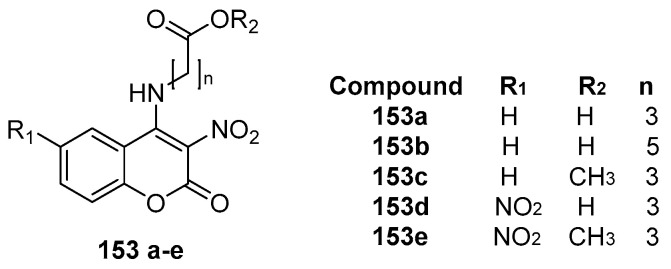
Chemical structure of compounds **153a**–**e**.

A series of 4-amino-3-nitrocoumarins (**153a–e**, Figure 48) was synthesized and biologically evaluated by Mokrov and co-workers [189]. The anticonvulsant activity was investigated using the MES mice model (grand mal seizures) and the corazole-antagonism test (modelling the so-called petit mal seizure). The only statistically significant result was given by compound **153a** at doses in the range of 60–80 mg/kg, as it could increase the animal survival in MES test up to 60% (in the control group survival was 10%). Corazole-induced seizures and animals’ death were not prevented by compounds **153a**, **153c** and **153d**. Compound **153e** exhibited good protection potential at 10–40 mg/kg and was able to prevent death in 50–63% of animals. The most active compound in the MES test was **153a**, containing a coumarin ring with a 3-nitro group and a γ-aminobutirric acid fragment. The corresponding methyl-ester (**153c**) did not show any activity, as well as compound **153d**, with an additional nitro group. Compound **153e**, bearing the methyl-ester of GABA pharmacophore and two nitro groups on the coumarin ring, showed the best profile in corazole-antagonism test. The corresponding free carboxylic acid **153d** was found to be inactive, as well as compound **153c**, having one less nitro group. Compound **153a**, bearing one nitro group and no substituent on GABA moiety, did not show any corazole-antagonist activity. Therefore, it can be speculated that compounds **153a** and **153e** possess different mechanisms responsible for their anticonvulsant activity and they may be a starting point for further studies on coumarin derivatives as anticonvulsant agents. 

### 2.10. Anticoagulant Activity

**Figure 49:**
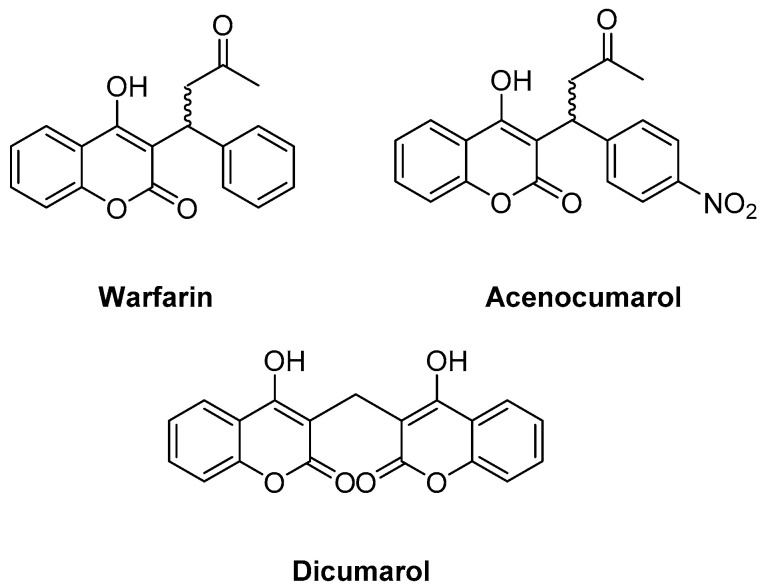
Chemical structure of the most common anticoagulant drugs.

The recent medical history of anticoagulant drugs has been largely dominated by a simple class of molecules that has been discovered thanks to a mysterious livestock mortality and, later, employed as a powerful rodenticide: coumarins. The anticoagulant activity of coumarins was identified when in 1920′s seemingly healthy cattle of Canada and North America died inexplicably for internal hemorrhages. The main cause of this decimation was attributed to a mold infestation of the damp hay, later known as the “sweet clover disease.” However, it was not before 1940 that the responsible molecule was identified by Karl Link and his student Harold Campbell: it was 3,3’-methylenebis(4-hydroxycoumarin), later known as dicoumarol. Further studies by Link’s team brought to the discovery of warfarin in 1948 (so named from WARF, Wisconsin Alumni Research Foundation, that financed the project), approved as a rodenticide in the USA in 1952 and for anticoagulation therapy in human in 1954, under the name of Coumadin. Currently, warfarin is one of the most widely used anticoagulation drug (in the UK it has been estimated that at least 1% of the population and 8% of people over 80 years are taking warfarin), together with other coumarin derivatives like dicumarol and acenocoumarol (Figure 49) [18,190,191,192]. 

Warfarin and other anticoagulant coumarins are vitamin-K antagonists (VKAs). Indeed, due to the structural similarity with vitamin-K, the compounds block the vitamin-K dependent pathways of coagulation, involving several factors (II, VII, IX and X). Despite the efficacy and the advantages of an oral therapy, warfarin is not devoid of side effects, mainly associated with bleeding and complications, like the narrow therapeutic range and interindividual genetic difference in pharmacokinetics, which requires a continuous monitoring of the patient [193,194]. 

**Figure 50:**
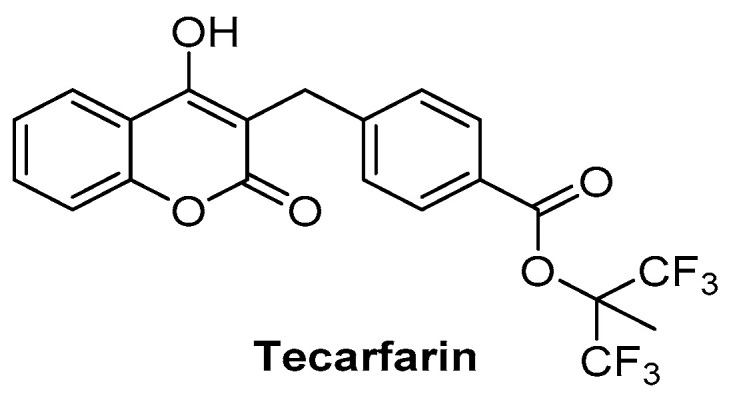
Structure of Tecarfarin.

For this reason, the research of new safer and efficient compounds lead to the discovery of a novel VKA, tecarfarin (ATI-5923, Figure 50), currently under development [195]. Tecarfarin is active after oral administration and acts as a vitamin-K epoxide reductase (VKOR) inhibitor; unlike warfarin, is not metabolized by the cytochrome P450 system but by human carboxylesterase-2 (*h*CE-2) in hepatic microsomes. Consequently, drug-drug or food-drug interactions are avoided, as well as genetic variability of CYP-450 system, providing a more stable anticoagulation effect compared to warfarin [196]. A detailed study on pharmacokinetics and pharmacodynamics of tecarfarin had been conducted on healthy patients by Albrecht et al., together with the recent phase I study on its tolerability among patients with severe kidney disease [197,198]. Tecarfarin has the potentiality to be a valid substitute of warfarin in the oral therapy of thromboembolic disease. 

**Figure 51:**
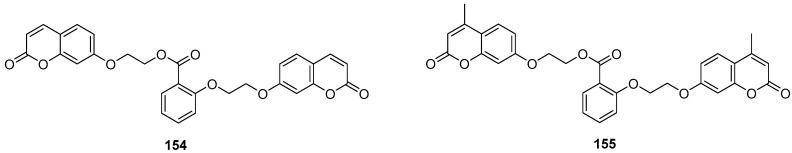
Chemical structure of coumarins conjugated with salicylic acid.

Another approach reported in literature is the chemical modification of the coumarin scaffold by conjugation of 7-hydroxylcoumarin and 7-hydroxy-4-methylcoumarin with some derivatives of salicylic acid. Among the compounds evaluated by Bang and co-workers in 2019, derivatives **154** and **155** (Figure 51) showed high anticoagulant activity, with an increased prothrombin time (PT) of 10.88 ± 0.56 s and 13.10 ± 3.56 s, respectively. Both compounds resulted 1.5 times more active than warfarin (PT 7.97 ± 1.93) [199]. 

**Figure 52:**
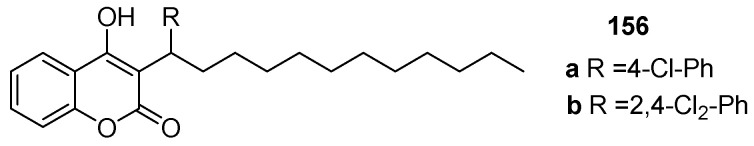
C-3 alkyl coumarin derivatives.

Structural modifications of the 4-hydroxycoumarin core was also the rational of Montagut-Romans et al., who in 2017 explored the potentiality given by modifications performed on C3 position by introducing a side chain (with at most one unsaturation) structurally related to vitamin-K cofactor [200]. The underlying premise was the SAR study performed by Gebaur in 2007, which pointed out that the activity of 4-hydroxycoumarin was enhanced only by structural modification for C3 position by isoprenyl motifs [201]. In this contest, 14 functionalized 4-hydroxycoumarins with alkyl chains of different length, both linear and branched, were synthesized and their activity was evaluated in vitro and ex vivo (phenprocoumon was included in the test as internal standard). The ability to inhibit VKORC1 in rat liver microsomes was evaluated in vitro and, except for two compounds, the C3-alkyl derivatives showed a sub-micromolar activity (from 20 nM to 200 nM) overcoming the benchmark compound phenprocoumon. Further ex vivo studies assessed the ability to increase in vivo the prothrombin time (PT) and compounds **156a** and **156b** (Figure 52) showed a promising anticoagulant activity after 24 h. It is possible that the presence of the halogen atom protects the drug from liver metabolism. Despite the interesting anticoagulant activity, follow-up studies on the liver metabolism are necessary to determine if these molecules are substrate of CYP2C9, to which is to attribute the variability in the dosage of oral vitamin-K antagonists, due to its polymorphism [202].

**Figure 53:**
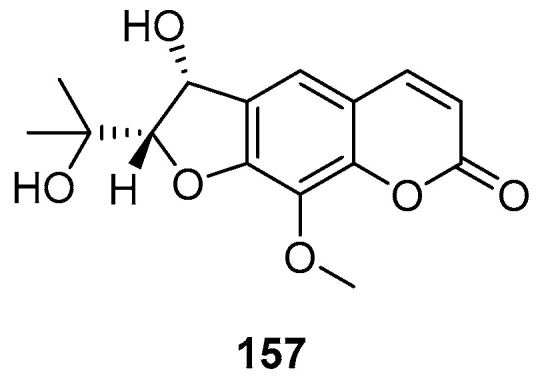
Coumarin derivative from *Ainsliaea fragrans*.

Concerning nature-derived coumarins, endowed with promising anticoagulant activity, in 2015 Lei et al. performed a phytochemical investigation on the Chinese herbal medicine *Ainsliaea fragrans* earning five new derivatives. The team evaluated the anticoagulant activity of all isolates via activated partial thromboplastin time (APTT), thrombin time (TT) and prothrombin time (PT) assays in vitro and in vivo. These studies concluded that one of the new compounds (**157**, Figure 53) presented a remarkable anticoagulant activity (PT = 41.2s and TT 128.5s) and no significant hepatic or renal toxicity when compared to warfarin (PT = 55.7s and TT 80.6s) [203]. Although further studies are necessary to understand the mode of action of compound **157**, it could be a promising anticoagulant agent for preclinical studies. 

### 2.11. Antidiabetic Activity

Diabetes is a chronic metabolic disease characterized by high blood sugar levels and is generally caused by an insufficient production of insulin by β-pancreatic cells or by the inability of human body to use this hormone. The consequences of diabetes might be very serious: blindness, kidney failure, stroke, heart attacks, lower limb amputation [204,205,206] 

**Figure 54:**
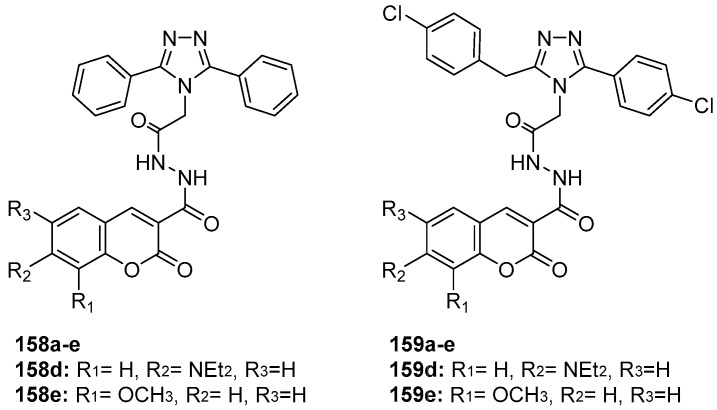
General structures of compounds **158a**–**e** and **159a**–**e**.

Menteşe et al. synthesized a novel series of *N*߰-(2-(3,5-disubstituted-4*H*-1,2,4-triazol-4-yl)acetyl)-6/7/8 substituted-2-oxo-2*H*-chromen-3-carbohydrazides (**158a–e**, **159a–e**, Figure 54) [207], merging the 1,2,4-triazole and the coumarin moieties, both characterized by a wide range of biological activities (including inhibition of α-glucosidases) and low toxicity profiles [208,209,210,211,212,213]. Then, their activity on α-glucosidases was studied, evaluating the enzyme inhibition in the presence of pNPG (*p*-nitrophenyl-α- D-glucopyranoside) as a substrate in the buffer (pH 6.8). Among the new compounds, four molecules showed high inhibition activity, compared to acarbose (IC_50_ = 8.85 ± 0.23 μg/mL): **158d** (IC_50_ = 4.28 ± 0.10 μg/mL), **158e** (IC_50_ = 0.96 ± 0.02 μg/mL), **159d** (IC_50_ = 6.75 ± 0.10 μg/mL) and **159e** (IC_50_ = 1.44 ± 0.06 μg/mL).

**Figure 55:**
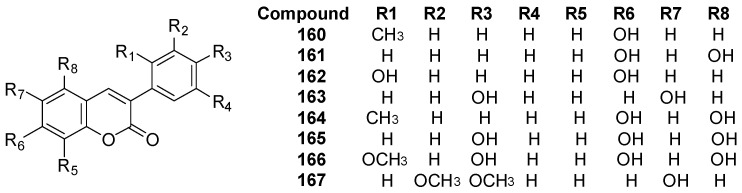
Chemical structure of compounds **160**–**167**.

Compounds **158e** and **159e** resulted the most active, probably because of the metoxy- group at position 8 of the coumarin ring. Derivatives without substituents on positions 3 and 5 of the phenyl ring linked to the triazole nucleus resulted more active than compounds bearing a chlorine atom or a phenyl moiety on such positions. According to kinetic studies, the tested compounds inhibit α-glucosidases in a competitive way. Other studies focused on coumarins-mediated inhibition of α-glucosidases were carried out by different groups. Hu and collaborators synthesized through microwave radiation heating a new series of more than forty 3-arylcoumarins which were screened for antioxidant activity, α-glucosidases inhibition and advanced glycation end-products (AGEs) formation inhibition [214]. Only eight of the synthesized compounds (**160–167**, Figure 55) exhibited moderate to high inhibitory activity on α-glucosidase. 

Compound **165** was the most promising (IC_50_ = 1.37 ± 0.67 μM), with a α-glucosidase inhibitory activity slightly weaker than acarbose (IC_50_ = 0.050 ± 0.003 μM). From an extensive SAR study, it emerged that the 7-hydroxy group is important for the inhibition of α-glucosidase. Compounds **160**, **163**, **164**, **165** and **166** were then tested in vivo: none of them showed toxicity on mice (LD_50_ > 5000 mg/kg). The mentioned compounds were also tested on streptozocin-induced diabetic mice. Compound **166** showed the best profile, exhibiting a strong reduction of glucose blood levels. Oral daily administration of **166** (30 mg/kg/day) restored glucose blood levels near normal values, showing an effect similar to that of the oral antidiabetic glibenclamide. 

**Figure 56:**
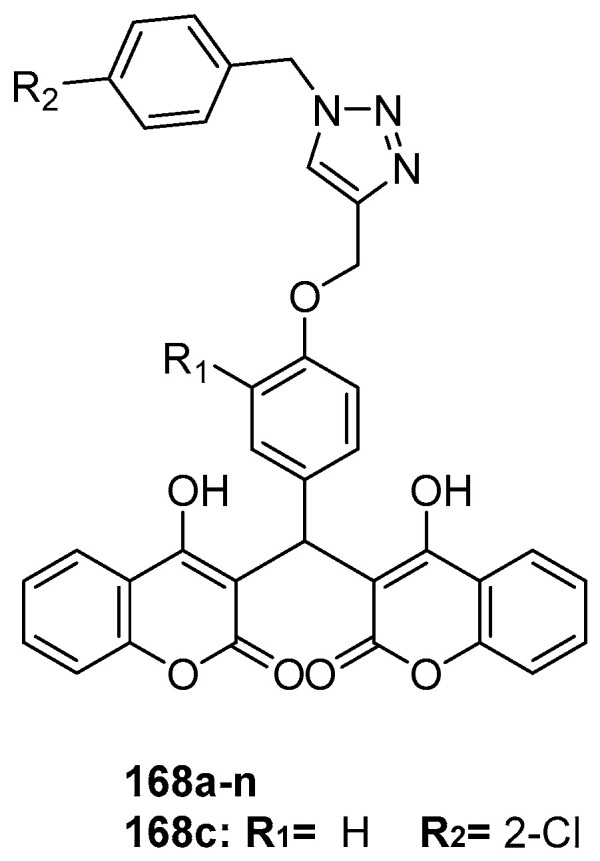
Chemical structure of biscoumarin-1,2,3-triazole hybrids. Substituents for compound **168c** are reported.

Asgari and co-workers synthesized a new series of biscoumarin-1,2,3-triazole derivatives (Figure 56) and evaluated their α-glucosidase inhibitory potential, using acarbose as a reference drug [215]. 

Here, again, two active moieties, both characterized by a wide range of biological activities, were merged together: the bis-coumarin and the 1,2,3-triazole moieties [216]. All the synthesized compounds showed excellent activities (IC_50_ between 13.0 ± 1.5 and 75.5 ± 7.0 μM) compared to acarbose (IC_50_ 750.0 ± 12.0). Compound **168c**, bearing 2-chloro phenyl moiety, resulted the most active. The substitution of the chlorine atom with a methyl group or its shift on the C4 position caused a decrease in activity. Moreover, the inhibitory activity seemed to depend importantly on the electron properties of the substituents. From further kinetic studies it emerged that compound **168c** inhibits α-glucosidases in a competitive mode (K_i_ = 11 μM).

**Figure 57:**
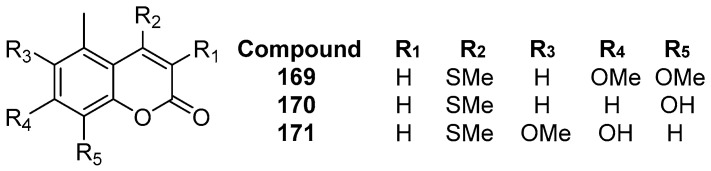
Chemical structure of compounds **169**, **170** and **171**.

A different therapeutic approach may be the stimulation of insulin secretion. In this perspective, Ahmed and collaborators extracted from the aerial parts of *Clutia lanceolata* (a medicinal plant native to sub-Saharan Africa and the Arabian Peninsula) twenty-one coumarins, including methyltio- and methylsulfinil-coumarins, thirteen of which were reported for the first time [217]. The structures of these natural compounds were elucidated from 2D-NMR and MS spectra, whereas their anti-diabetic activity was tested measuring the glucose-triggered insulin secretion of freshly isolated murine islets. Compounds **169**, **170** and **171** (Figure 57) resulted the most active in stimulating glucose-triggered insulin release, compared to glimepiride. Further studies are needed to understand structure-activity relationships in order to develop new active compounds.

## 3. Other Applications of Coumarin Scaffold

### 3.1. Coumarins Photoproperties

The applications and properties of coumarin scaffold have remarkably wide boundaries. Coumarin-based compounds have been exploited in numerous research and industrial sectors, as active pharmaceutical ingredients, pesticides, fragrances, dyes for several purposes from laser technology to organic photoredox catalysis, cell imaging, photocleavable protecting groups and fluorescent biological probes [6,218,219,220,221,222,223,224,225]. In the following paragraphs, the most recent applications associated with the photophysical properties of coumarins have been reviewed. 

#### 3.1.1. Coumarins as Photocleavable Protecting Groups (PPGs)

The development of a suitable formulation is a crucial step in order to achieve new functional therapeutics. The design of novel strategies aimed at selectively release the bioactive in a specific district at a determinate time to maximize efficacy and reduce off-target adverse effects represents an extremely active research frontline. So far, various stimuli-responsive systems have been considered in therapeutic approaches to regulate the release of the therapeutic cargo, including endogenous stimuli (e.g., pH, enzymes, redox reactions, etc.) and exogenous stimuli (e.g., light, magnetic field, ionizing radiations, etc.) [226,227]. Light-mediated therapies have shown excellent results in achieving on-demand therapeutics and optical tools for studying and controlling complex chemical and biological processes in localized areas, owing to their superior non-invasiveness and spatiotemporal precision upon applying a specific light-irradiation wavelength [227,228,229]. One method for the regulation of molecular processes with light is the use of photolabile “protecting” groups in key locations. Ideally, this modification completely blocks the activity of any molecule and restores it only with light [230].

Coumarins, particularly 4-hydroxymethyl derivatives, are known to undergo photolysis. Keeping this concept in mind, several biomolecules of interest have been linked to the coumarin nucleus, mostly as acyl derivatives. Then, under UV irradiation, the biomolecules can be released in biological systems. The photophysical parameters of the formed derivatives are determined by different factors as the mode of fusion, the chemical nature of additional rings and the presence of electron-donating and electron-withdrawing substituents [12].

**Figure 58:**
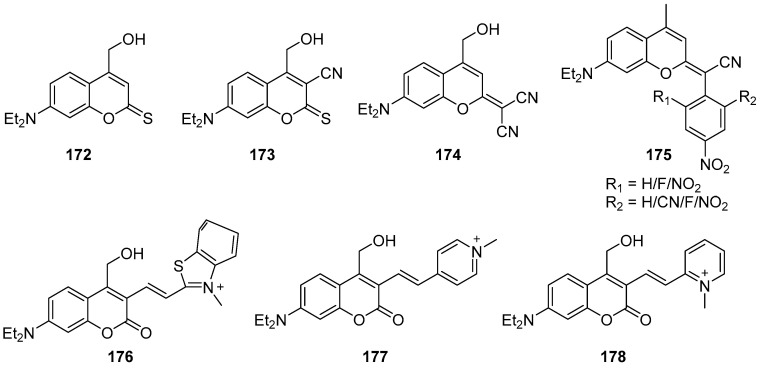
Structure of several coumarin photocleavable protecting groups.

Fournier and co-workers in 2013 proposed a series of methyl-coumarins with redshifted absorption. In particular, three compounds (**172**–**174**, Figure 58), were synthetically easily accessible and exhibited a significant action cross section for uncaging with blue-cyan light, whereas their uncaging ability in the UV spectral domain remained low in order to avoid their photoactivation when a properly tuned UV illumination is applied [231]. 

In the same year, Fournier and co-workers further proved that compound **172** was a good blue-absorbing caging group, owing to its strongly donating substituent conjugated to the thiocarbonyl group. Moreover, the research team demonstrated that this particular caging group could be used in zebrafish embryos in the context of development biology to perform chromatic orthogonal photoactivation of two biologically active species [232].

In 2017, Gandioso and colleagues reported the development of green/red-absorbing chromophores based on coumarin scaffolds that could be useful as photocleavable protecting groups [224]. A series of coumarin derivatives in which the carbonyl of the lactone was replaced by a cyano(4-nitrophenyl)methylene moiety, by condensation of a thiocoumarin precursor with the corresponding arylacetonitrile derivatives, was synthesized and subsequently refined with the insertion of electro-withdrawing groups at the phenyl ring, leading to absorption in the green to red region (**175**, Figure 58) [224]. The insertion of more than one electro-withdrawing group (such as -NO_2_ and -CN) decreased the fluorescence emission, whereas the mononitro-containing coumarin derivatives had a strong emission in the red region upon excitation with green light, as denoted by their significantly large Stokes shifts. In order to demonstrate the utility of these new compounds as PPGs, a small collection of coumarin-based photocages of benzoic acid was prepared. Thanks to photolysis studies with green light, it was demonstrated that the structure of the coumarin chromophore influenced the rate of the uncaging process. This observation gave the opportunity to exploit these new coumarin scaffolds as caging groups removable with visible light. On the other hand, Bojtar and colleagues proposed water soluble red-shifted coumarin caging groups (**176–178**, Figure 58), activated with green-light [233]. 

The optical properties of coumarins as photo-responsive unites could be also applied to polymers that after a photochemical activation rapidly degrade into small molecules. In 2018, Iturmendi and co-workers proposed that, through functionalization of polyphosphazenes with a coumarin-caged amino acid as a pendant group along the backbone, the sensitivity of the polymers to hydrolysis would be accelerated upon irradiation and effectively catalyze its own degradation [234].

#### 3.1.2. Coumarins as Fluorescent Probes

**Figure 59:**
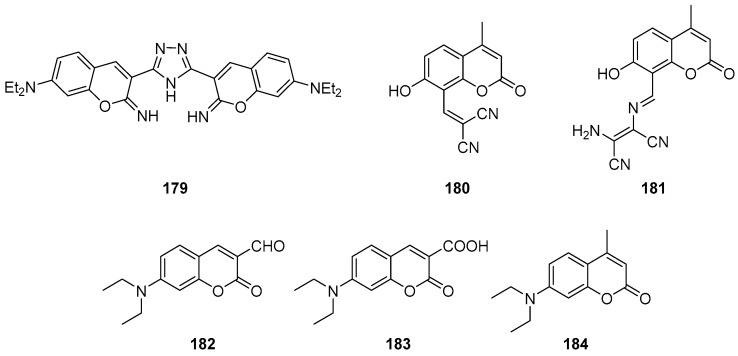
Coumarin florescent probes for hypochlorite ion detection.

Coumarins possess a large electron-rich π-π conjugated system with charge transfer properties, reason why coumarin-based fluorophores are widely used for monitoring a variety of biologically important species and biochemical process in living cells, for example as diagnostic agent for detection of biothiols, enzymes, mitochondrial pH values, glucose and ions [3,222,235]. In particular, several coumarin scaffolds have been proposed and evaluated for the detection of ions in different fields, from cellular imaging to environmental waters. Gong and co-workers based their work on an easily synthesized coumarin-based fluorescent probe (**179**, Figure 59) that already was effective in the detection of glutathione (GHS) in the presence of Cu^2+^ ions, expanding its potentiality to the detection of hypochlorite ions with high selectivity and sensitivity. The probe showed a remarkable fluorescent intensity change in response to hypochlorite ions; moreover, this probe could be applied to detect ClO^−^ in cells via intracellular fluorescent imaging [236,237]. Given the importance of hypochlorite ions both in living systems (being one of the biologically most important reactive oxygens species) and in the environment (owing to its use as disinfectant), in 2019, Shangguan and colleagues proposed another probe for this particular ion based on coumarin dye and malononitrile (**180**, Figure 59). The fluorescence response of probe **180** at 459 nm towards hypochlorite ions gradually enhanced with the increase of ClO^−^ concentrations, resulting in 45-fold fluorescence enhancement. Furthermore, probe **180** exhibited high accuracy for quantitative measurement of hypochlorite ions in real water samples and it can be used as a potential chemosensor for the detection of ClO^−^ in chemical environmental and biological systems [238]. In the same year, Tang and co-workers worked on a coumarin based fluorescent probe (**181**, Figure 59) able of rapidly discern hypochlorite and copper(II) ions in water sample and biological systems. Upon the reaction with ClO^−^, the fluorescence wavelength of **181** displayed a strong blue shift along with the naked-eye visible changes from yellow to colorless. Moreover, it exhibited an obvious fluorescence quenching behavior to copper(II) with colorimetric analysis from yellow to luminous yellow [239]. 

A noteworthy research on the mechanism of interaction between coumarin based ionic probes and hypochlorite ions had been conducted by Starzak and collaborators [240]. First, the research team confirmed the linear decrease in the fluorescence emissions together with the increase in ClO^−^ concentration of three different coumarin derivatives, **182–184** (Figure 59), which were selected because of the presence of the 7-diethylamino group and the 3-substituted lactone ring that are well-known structural pattern accountable for the fluorescence properties. It was observed a different reactivity profile depending on the pH levels, probably due to the different reactivity of hypochlorite ions ascribable to the variation of the dissociation of the salt at different pH values. Afterwards, a deeply investigation on the possible formation of chlorinated derivatives was conducted: HPLC-PDA-ESI-MS analyses highlighted the presence of chlorinated derivatives and proved that the chlorination reaction was responsible for the linear fluorescence decays. The results suggest the possibility to exploit these coumarin ionic probes for the detection and quantitative determination of hypochlorite species in vivo. 

**Figure 60:**
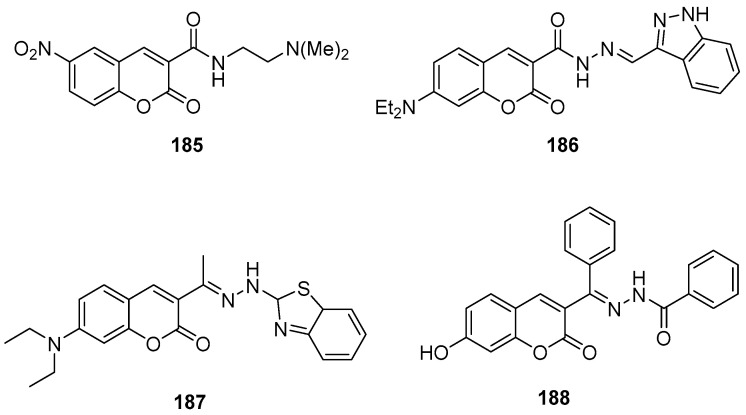
Coumarin fluorescent probes for copper (II) detection.

A coumarin fluorescent probe based on a nitro-3-carboxamide derivative for selective copper (II) ions detection was reported by Bekhradnia and colleagues. Compound **185** (Figure 60) showed the highest fluorescence intensity in presence of Cu^2+^ compared to a variety of other common heavy and toxic metal ions (for example Pb(II), Co(II), Hg(II)) and in aqueous solution at 320 nm [241]. Another approach was attempted for the selective detection of copper (II) ions by He et al. in 2018, which based the fluorescent probe on a coumarin-Schiff base derivative (**186**, Figure 60). This probe resulted to be particularly selective for Cu^2+^ even in the presence of several other ions [242]. Saravana Mani and colleagues designed in 2019 a coumarin hydrazine-based fluorescent probe for the detection of copper(II), called BENZEPYR (**187**, Figure 60), exploiting a reaction of condensation between 2-hydrazino benzothiazole and *N,N*′-diethylamino-3-acetyl coumarin [243]. This particular fluorescent chemosensor could selectively detect Cu^2+^ among other disturbing metal ions, resulting particularly specific and highly responsive, with a visible colorimetric change of the solution, which turned from yellow to wine red. Moreover, the limit of detection (LOD) had been estimated to be 40 nM. BENZEPYR **187** was also tested for the fluorescence bioimaging of Cu^2+^ ions in HeLa cells using fluorescence microscopic analysis, resulting suitable for the exploitation as an ion marker in living cells. 

Another remarkable case of coumarin-based chemosensor had been presented by Li and co-workers, who synthesized a multifunctional probe able to selectively detect not only copper (II) ions but also Al^3+^ ions and amino acids Lys and Arg [244]. In particular, the detection of Lys and Arg took place with a colorimetric (from yellow to colorless) and a fluorescent response (from a maximum absorption at 335 nm to 429 nm). At the same time the probe detected either way the presence of Cu^2+^ ions but could be used only for the fluorescent sensing of Al^3+^. A further interesting exploitation of probe **188** (Figure 60) was the fluorescent and colorimetric identification of Cys, Hcy and GSH when it was complexed with copper ions. 

**Figure 61:**
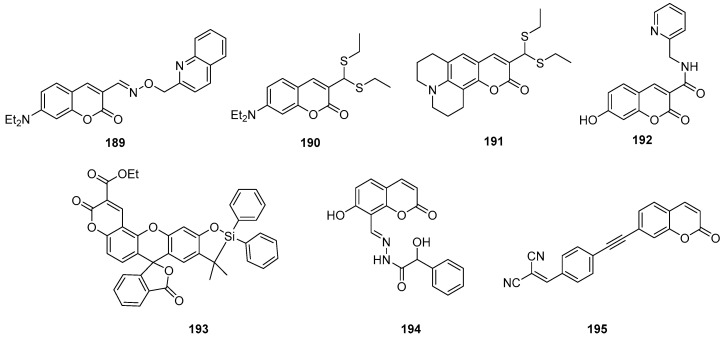
Chemical structure of different coumarin fluorescent probes for the detection of ions.

Despite the key role of chemical species like copper and hypochlorite, they are not the only ions valuable for detection. For this reason, a dual coumarin probe, fluorescent and colorimetric, was designed by Chen and colleagues for the detection of palladium (II) ions that can be used in living cells. This oxime-ether coumarin probe (**189**, Figure 61) exhibited a strong green fluorescence with an emission peak at 500 nm. When palladium(II) was added to the solution with compound **189**, the fluorescence intensity at 500 nm decreased consequently, until 2 equivalents of Pd^2+^ were reached; at that point the fluorescence was almost completely quenched, which could clearly be observed with the naked eye. A linear fit between fluorescence and palladium (II) concentration was observed in the range 0.0–8.0 μM, while the detection limit was measured to be 40 nM, which is far lower than the threshold for palladium content in drugs (5.0 ppm to 10.0 ppm - 47.0 mM to 94.0 mM) specified by the World Health Organization [245]. 

It is also noteworthy the development of thioacetalised coumarin based fluorescent probes for the detection of mercury (II), a hazardous ion both for human health and reproduction. Cheng and co-workers exploited the known Hg^2+^ promoted deprotection reaction of dithioacetals to design two novel reactive fluorescent probes (**190**, **191**, Figure 61) that showed a different behavior due to the different chemical structures: **190** displayed remarkable fluorescence quenching with the addition of Hg^2+^ ions while, in the presence of mercury ions, **191** displayed ratiometric fluorogenic and chromogenic response [246]. 

It should not be forgotten an on-off fluorescent probe for the tracking of iron (III) ions based on 7-hydroxy-2-oxo-*N*-(pyridin-2-ylmethyl)chromene-3-carboxamide. In their work, Warrier and Kharkar demonstrated that compound **192** (Figure 61) was selective towards Fe^3+^ ions and exhibited high fluorescence emission profile at 447 nm. The presence of other ions did not interfere with the detection of iron (III) ions and the limit of detection was found to be 0.76 μM. Moreover, cell imaging and MTT (3-(4,5-dimethylthiazol-2-yl)-2,5-diphenyltetrazolium bromide) assay proved the potential utility of probe **192** as cell-permeable chemosensor of Fe^3+^ in living cells [247]. 

The approach of Jiao and colleagues for the detection of fluoride ions was based on the linkage between the coumarin scaffold and fluorescein in order to obtain a highly selective and sensitive fluorescent probe. The mechanism of F^−^ ions detection by compound **193** (Figure 61) was explained and involved a desilylation reaction in the presence of fluoride ions. Moreover, a linear relationship between the ratio of emission intensities at 532 and 465 nm and F^−^ concentration over the range of 0–20 μΜ with a limit of detection of 0.025 μΜ was found [248]. Differently, Yao and co-workers exploited the capability of fluoride ions to form stable complex with Ca^2+^ to design a novel fluorescent sensor (**194**, Figure 61), synthesized from the combination of mandelic acid with 7-hydroxy-8-formylcoumarin through a hydrazine hydrate bridge, in order to selectively identify these two ionic species over other metal ions [249]. The fluorescence spectrum of compound **194** clearly increased when calcium ions were added to the solution with a limit of detection of 5.81 × 10^−7^ M, while, once the complex between the probe and Ca^2+^ ions was obtained, the addition of fluoride ions to the solution lead to the turn-off of the fluorescence response with a limit of detection 4.28 × 10^−7^ M. Moreover, bio-imaging studies were performed in order to assure the possibility to exploit this novel chemosensor for the identification of Ca^2+^ and F^−^ ions in vivo, with positive outcome. 

Finally, Reddy and Choi designed and synthesized three dicyanovinylcoumarin probes as turn-on fluorescent sensor for the detection of CN^−^ ions among other anions [250]. Within the different synthesized probes only compound **195** (Figure 61) showed a remarkable increase of the fluorescence in presence of fluoride and cyanide ions with an interesting sensibility towards CN^−^ ions: the limit of detection was up to 11.4 nM, lower than the maximum level in drinkable water according to WHO guidelines.

### 3.2. Food Systems

**Figure 62:**
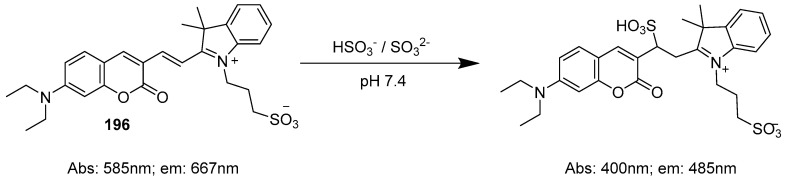
Chemical structure of probe **196**.

Coumarins have found important applications also in the agri-food sector. In fact, the antimicrobial activity characterizing these natural compounds could be exploited for food preservation or for the treatment of plant pathogens, infections in aquaculture or biofouling caused by eukaryotic organisms. In addition, because coumarin scaffold has been used in fluorescent probing, natural coumarins might be used in the detection of some substances in food samples. For instance, Zhang and collaborators developed a new near-infrared probe constituted by a conjugated coumarin-indolium system, for rapid, colorimetric and ratiometric fluorescent detection of bisulfite and sulfite anions [251]. (Bi)sulfite anions (HSO_3_^−^/SO_3_^2−^) are widely used as preservative for foods and beverages in order to prevent oxidation, browning and microbial reaction during products’ life cycle [252,253]. Unfortunately, high doses of (bi)sulfites can cause asthma or other allergic reactions. Some individuals are very sensitive even to low levels of these anions [254]. In addition, sulfur dioxide (SO_2_) is one of the most distributed pollutants and it has a relevant impact on human health [252], [253,255]. An efficient tool to detect such molecules is provided by fluorescent probes; to date, different probes for HSO_3_^−^/SO_3_^2−^ have been designed but many of them are intensity-based, which means that the signal output can be conditioned by different factors such as instrumental efficiency, probe concentration, environmental conditions. In addition, many of these probes show emissions only in the visible region and some of them need ultraviolet excitation, having limited biological applications. Thus, new, more efficient probes are required. In Zhang’s work, a conjugated coumarin-indolium system with intramolecular charge transfer (ICT) effect was developed, merging an electron-donating 7-diethylamino coumarin moiety and an electron-withdrawing positively charged indolium derivative, connected through an ethylene linker (Figure 62). The whole system resulted in a typical ‘push-pull’ large conjugation dye system with ICT properties. The so-designed probe showed NIR fluorescence (667 nm) and a rapid, highly selective and sensitive detection process for HSO_3_^−^/SO_3_^2−^ in aqueous solution under mild conditions; in addition, this probe exhibited significant colorimetric, NIR fluorescent and ratiometric signal responses upon one excitation wave and a detection lower limit of ∼27 nM. This probe can be applied to detect HSO_3_^−^/SO_3_^2−^ in real food samples, serum samples and living cells. When tested on real food samples (sugar, soft sugar, crystal sugar and wine in aqueous solution) this probe proved to be able to determine HSO_3_^−^ with good recovery. In addition, a paper test strip was developed, simply by wetting a strip of neutral filter paper with a solution of the probe in methanol (200 μM). The result was a deep blue test paper ready for use: when a HSO_3_^−^ solution is spotted on the test paper, rapid color changes can be observed, even if many other ions are present in the sample. Different colors correspond to different concentrations of HSO_3_^−^. Thus, probe **196** (Figure 62) can be used to develop a cheap, easy-to-prepare and easy-to-use paper test strip system for the detection of (bi)sulfites.

**Figure 63:**
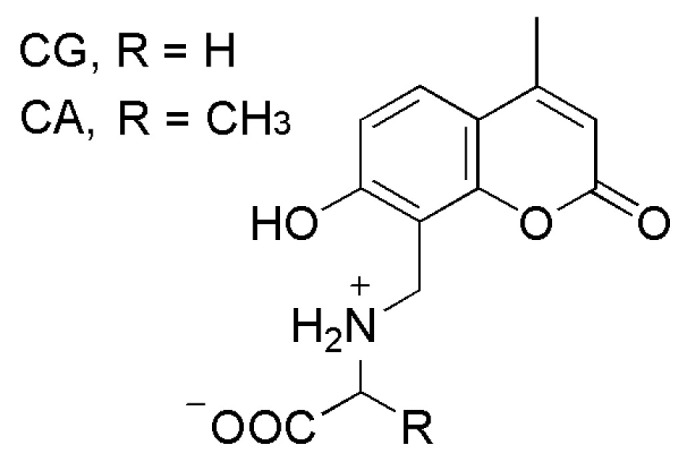
Chemical structure of CG and CA probes.

A more recent example is a probe developed by Nair and collaborators, able to selectively detect the amphiphilic bisulfate ion (HSO_4_^−^) in edible plant foods, dog urine and drugs [256]. Bisulfate consumption normally takes place through the ingestion of different edible plants, such as cabbage, broccoli, Brussels sprouts, horseradish or seeds (black and white mustard, for instance). These plants contain glucosinolates [257,258] that are hydrolyzed in our organism by the enzyme myrosinase, thus producing bisulfate ion [259]. In addition, bisulfate salts of many APIs are currently on market, constituting another source of bisulfate ions [260]. When trying to evaluate the actual concentration of HSO_4_^−^, it is important to take account of the deprotonation equilibrium between HSO_4_^−^ and SO_4_^2−^, using a highly selective probe able to discriminate between these two ions. Nair and co-workers developed two fully water-soluble probes, coumarin-integrated glycine (CG) and coumarin-integrated alanine (CA) zwitterions, for the selective detection of HSO_4_^−^ at picomolar level (from 50 to 325 pM) (Figure 63). 

Glycine and alanine can interact selectively with the target through H-bond, due to their zwitterionic nature at physiological pH, whereas the 7-hydroxycoumarin moiety constitutes a good fluorophore probe, thanks to its biocompatibility, non-toxicity and water solubility. The CG/CA probes proved to be able to penetrate and stain living cell. When different unknown concentrations of clopidogrel bisulfate were added to a water solution of CG/CA, it was possible to precisely measure such concentration by measuring the emission intensity of each sample. Confirmation of the experimentally observed values with the theoretically calculated ones supported the accuracy of the presented method. CG/CA probes were also tested on food samples: water extracts of cruciferous plant foods (cabbage, broccoli, mustard seeds, carrots) were added to aqueous solutions of the probes. Again, titration of bisulfate ions was performed by emission measurements—addition of increasing volumes of aqueous food saps caused a growing reduction of probe’s emission. Cucumber and fenugreek were used as controls, as they do not contain bisulfates. Eventually, bisulfate content was measured in pet urine samples (bisulfate is one of the common components of pet foods): urine samples were collected from two adult dogs and were treated with aqueous solution of CG/CA; a reduction of probe emission was noticed, revealing the presence of HSO_4_^−^ (quenching was proportional to bisulfate quantity). In conclusion, CG and CA probes demonstrated the ability to detect bisulfate ions in aqueous solution at pH = 7.4, wherein HSO_4_^−^ concentration was 10^5.4^ lower than that of SO_4_^2−^, at concentration as low as 50 pM, even in presence of other ions.

With regards to the antimicrobial activity of coumarin scaffold, some studies have been recently carried out, showing the potential of natural coumarins as food preservatives. Yang and co-workers have studied the antimicrobial activity of eighteen natural compounds against *R. Solanacearum* [261]*,* a bacterium responsible for the wilting of different plants such as tobacco, tomato, potato in (sub)tropical regions, causing significant economic losses [262,263]. 

**Figure 64:**
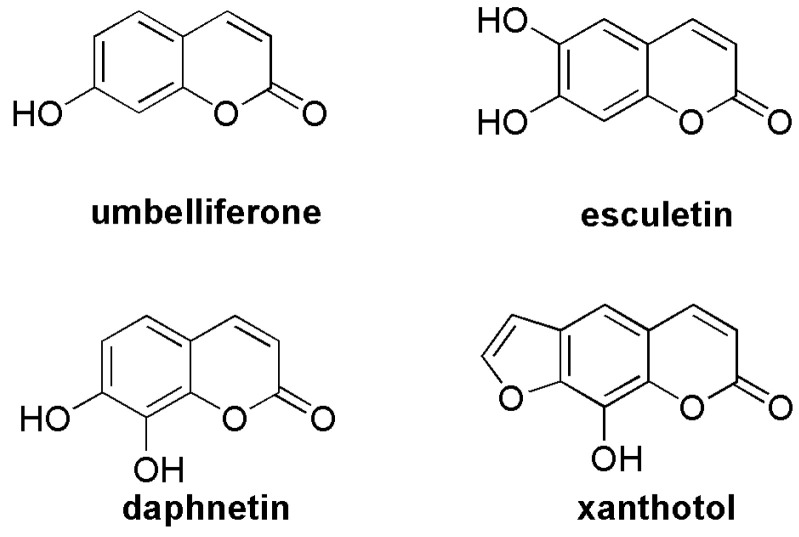
Chemical structure of coumarins umbelliferone, esculetin, daphnetin and xanthotol.

Among them, four coumarins (Figure 64) showed an antibacterial activity stronger than that of thiodiazole copper treatment (antibacterial rate (MBC/MIC) of 63.3%). Daphnetin showed the highest activity, followed by xanthotol and esculetin (antibacterial rate 97.43%, 80.12% and 71.44%, respectively). Antibacterial activity seemed to be enhanced by C6, C7 or C8 substitution, so hydroxycoumarins umbelliferone, esculetin and daphnetin were selected for further investigation of the mechanism of action. Hydroxycoumarins were tested from 10 to 100 mg/L concentrations and from results it was clear that the good activity of umbelliferone can be enhanced by the additional hydroxylation of C6 position (esculetin), whereas even better results can be achieved by the dihydroxylation of C7 and C8 positions (daphnetin). TEM images of *R. solanacearum* showed that daphnetin and esculetin caused irreversible damages to the cell membrane, whereas umbelliferone must follow a different path in inducing cell damage. It is worth noting that hydroxycoumarins showed very low cytotoxicity on human cells and have no effects on tobacco seeds’ germination. Furthermore, because *R. solanacearum* forms biofilm-like aggregations on host’s roots, facilitating bacterial infection [264], Yang and his group speculated that hydroxycoumarins may interfere with biofilm formation. In fact, daphnetin, umbelliferone and esculetin (100 mg/L) reduced biofilm formation by 99.22%, 85.20% and 93.90%, respectively, probably influencing the swimming motility of the bacterium. Thus, the expression of the regulating and structural flagellar genes *fliA*, *flhC* and *flhD* in presence of daphnetin, umbelliferone and esculetin was evaluated and it turned out that the expression of *fliA* and *flhC* was significantly repressed by the mentioned compounds. 

**Figure 65:**
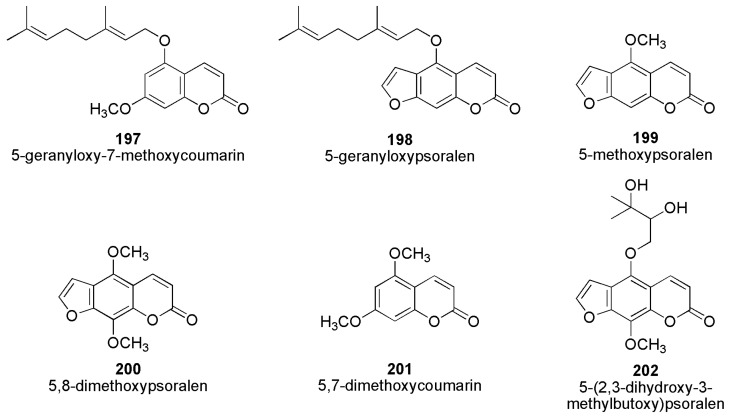
Structures of compounds isolated from *Citrus* peel.

Another interesting biological activity ascribed to the coumarin nucleus is its antifungal activity, which can be useful not only in the medicinal field but also in the agricultural one. As far as the latter is concerned, there has been an increasing interest in the exploitation of coumarin scaffold for the design and synthesis of novel fungicides useful in the treatment of many plant diseases. Many pathogenic fungi still cause massive death of crops, limiting production and causing significant financial losses. To date, farmers deal with this problem by using chemical fungicides but such solution comes with some drawbacks: the long and extensive use of these chemicals may cause environmental pollution, accumulation of pesticides residues in the plants and drug resistance by fungi. Thus, there is an urgent need for alternative (and green) solutions. In 2019, Ramìrez-Pelayo and his collaborators studied the coumarin-based compounds contained in the peel of citrus grown in Colombia [265]. Six coumarins were isolated (**197–202**, Figure 65) and their antifungal activity, in terms of mycelial growth and spore germination inhibition, was evaluated against the fungus causing anthracnose (*Colletotrichum* sp.). The production of oranges, mandarins, grapefruit, lemons, limes is limited by the action of microorganisms able to proliferate in such fruits because of peculiar condition such as high nutrient content, humidity and low pH values. Nevertheless, these plants can defend themselves from pathogens thanks to the production of antimicrobial compounds which can be constitutive (phytoanticipins) or induced (phytoalexins) [266,267]. 

**Figure 66:**
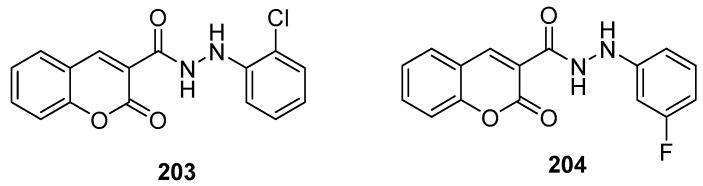
Chemical structure of compounds **203** and **204**.

As already mentioned, from the methanol extracts of *C. latifolia* and *C. aurantifolia* peels six compounds were isolated—5-geranyloxy-7-methoxycoumarin (**197**), bergamottin (**198**), bergapten (**199**), isopimpinellin (**200**), limettin (**201**) and oxypeucedanin hydrate (**202**) and their activity was compared to that of umbelliferone, scoparone and scopoletin. Compounds **197**–**201** were tested at 0.25, 0.50 and 1.00 mM, whereas **202** was tested at 1.00 mM concentration. Carbendazin and thymol were used as reference compounds (0.5 mM). The results showed that compounds **197**–**202** significantly inhibited mycelial growth of *Colletotrichum* sp., the activity being proportional to the dose used. The highest inhibition was exhibited by compounds **199** and **201** (32% and 25%, respectively); therefore, the fungistatic effect of different mixtures of **199** and **201** (0.25 mM (**192**):0.75 mM (**201**), 0.50 mM (**199**):0.50 mM (**201**) and 0.75 mM (**199**):0.25 mM (**201**)) was investigated, each of them showing enhanced activity respect to both the individual compounds. Furthermore, the activity of **199**, **201** and their mixtures was compared to that of the phytolaxins scoparone, scopoletin and umbelliferone; the results showed that phytolaxins were just slightly more active than the isolated compounds, although the highest fungistatic activity was shown by the mixture of **201** (0.75 mM) and **199** (0.25 mM). This mixture, along with compounds **197**, **199** and **201**, was selected for the evaluation of the inhibitory effect on spore germination in comparison with scopoletin, scoparone and umbelliferone. Compounds **201** and **199** exhibited very good activity, with 96.7% and 95.3% inhibition, respectively but these percentages rapidly decrease, being less than 5% after 24 h. Again, the **199**/**201** mixture showed the highest activity causing a complete, long-lasting inhibition of spore germination, thus suggesting that there may be an additive or synergistic effect between these compounds. Therefore, coumarins and furanocoumarins may be useful scaffolds in the design of new antifungal agents. In 2018, Yu and co-workers designed a series of coumarin-3-carboxamide derivatives and evaluated their antifungal activity against *Botrytis cinerea*, *Alternaria solani*, *Gibberella zeae*, *Rhizoctonia solani*, *Cucumber anthrax* and *Alternaria* leaf spot [268]. These phytopathogenic fungi are all relevant in agriculture, because they can cause significant losses in crops and investments. In this work, two different scaffolds were merged in order to design a new category of more effective fungicides. In fact, the use of carboxylic acid amides (CAA) in this context is well-established, because amide fungicides have been used for over 50 years [269]. More recently, new amide fungicides with high activity and broad spectrum of action were developed (e.g., Fluopyram, Bixafen, Sedaxane, Isopyrazam, Penthiopirad and Boscalid) [1,270]. In addition, hydrazide scaffolds have been found to have interesting biological and pharmacological activities [271,272]. Similarly, the antifungal activity of coumarin nucleus is well known (paragraph 0). Considering these elements, the authors decided to combine the coumarin and the carboxamide/hydrazide scaffolds with the aim of synthesizing high-performance fungicides. All the synthesized compounds were tested and their antifungal activities against the mentioned phytopathogenic fungi were evaluated. At 100 μg/mL concentration, most of the tested compounds showed poor antifungal activity. Only compounds **203** and **204** (Figure 66) showed EC_50_ values of 1.57 μg/mL and 1.65 μg/mL, respectively, against *Botrytis cinerea*, proving to be equivalent to the reference Boscalid (0.51 μg/mL), whereas **203** (EC_50_ = 1.80 μg/mL) resulted more active than Boscalid (EC_50_ = 2.98 μg/mL) against *Rhizoctonia solani.*

**Figure 67:**
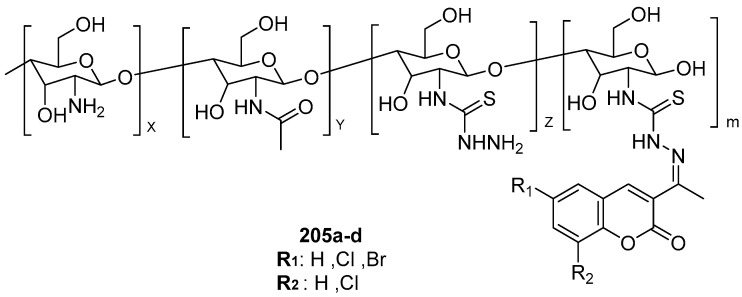
Chemical structure of coumarin-functionalized chitosan **205a**–**d**.

Starting from these data it was possible to suggest some structure-activity relationships: the replacement of amide bond with a hydrazide bond led to an improvement in antifungal activity spectrum. Furthermore, coumarins bearing amide group resulted to be remarkably more active against *Botrytis cinerea* and *Rhizoctonia solani,* whereas against *Alternaria solani*, *Gibberella zeae*, *Cucumber anthrax* and *Alternaria* leaf spot they showed less potency. It is noteworthy that compounds bearing an electron-withdrawing group did not show any activity, whereas compounds with an electro-donating group were active against *Alternaria* leaf spot. The addition to the coumarin nucleus of Cl/F- substituted phenylidrazine gave compounds active on *Cucumber anthrax.* A similar approach is the one followed by Yang and collaborators, who decided to exploit coumarin nucleus for the functionalization of chitosan, in order to create more effective chitosan-based fungicides [273]. In fact, chitosan is becoming more and more appreciated due to its antimicrobial activity, low toxicity, biodegradability, biocompatibility and film forming activity. However, its application as fungicide is limited by its low solubility and weaker activity respect to the on-market fungicides. In this work, four coumarin-functionalized chitosan derivatives (**205a–d**, Figure 67), were synthesized and tested against the phytopathogens *Alternaria solani sorauer, Fusarium oxysporum* f.sp*. vasinfectum* and *Fusarium moniliforme* by evaluating the mycelial growth rate in vitro. At 1.0 mg/mL, compounds **205a**–**d** showed inhibitory activity against *A. solani*, following the sequence **205d** > **205b** > **205c** > **205a** (38.25%, 51.23%, 44.01% and 52, 78%, respectively). 

These results suggested that the introduction of halogens caused an increase in activity, depending on the number and the type of halogens. For instance, the 3,5-dichloro derivatives were more active against *A. solani* than the correspondent 5-Cl or 5-Br derivatives, probably because of an increase of hydrophobicity. The four coumarin-chitosan derivatives showed higher activity than chitosan alone also against *F. oxysporum* and *F. moniliforme*. Compound **205d** showed an inhibitory index of 57.09% against *A. solani*, 77.24% against *F. oxysporum* and 66.12% against *F. moniliforme*. 

### 3.3. Coumarin-Metal Complexes

The great biological potential of coumarins might be even increased by considering the possibility to complex coumarin scaffold with other substances as, for example, some metals. In fact, it has been proved that it is possible to enhance the activity of a certain drug simply binding it to a metallic-element [274]; moreover, by combining known active moieties with metals, it would be possible to improve the parent compound with a certain selectivity or even with a new mechanism of action. Therefore, many groups have already started to explore this strategy, aiming to produce more active compounds, exploiting metals as copper, platinum, zinc or silver. In some cases, due to its intrinsic potential, coumarin scaffold was exploited in this approach. Some examples are discussed below.

**Figure 68:**
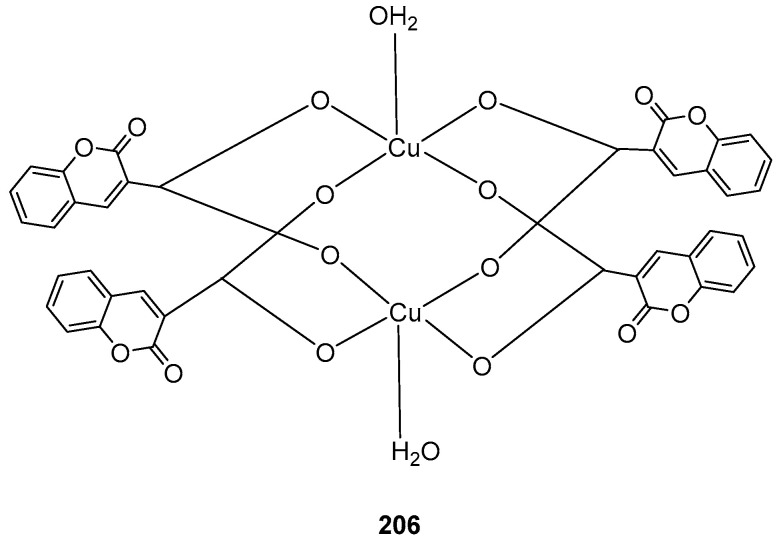
Chemical structure of coumarin-3-carboxilate-copper (II) complex (**206**).

The nature of coumarin-copper complexes had already been evaluated in 2001 by Karaliota and co-workers, who synthesized and characterized a binuclear coumarin-3-carboxilate-copper (II) complex (**206**) [275]. In this work, thanks to multiple instrumental characterization by means of IR, Raman and NMR spectroscopy, the authors were able to identify the structure of the complex (Figure 68): a binuclear molecule [Cu_2_(Cca)_4_(H_2_O)_2_], where copper is coordinated by four carboxylic oxygens, one from each Cca molecule and two water molecules.

**Figure 69:**
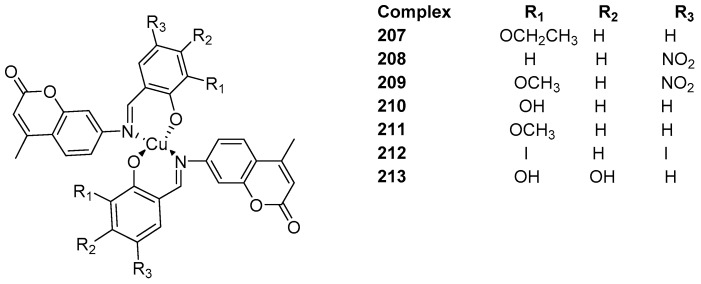
Chemical structure of complexes **207**–**213**.

A more recent work by MacLean et al. reports a series of copper (II) complexes with seven imine-derived ligands (**207**–**213**, Figure 69) which were tested on MCF-7 human breast cancer cells to evaluate their cytotoxicity [276]. In fact, the increase of ROS levels might promote oxidative stress-induced cancer cell death, thus representing an alternative strategy for the treatment of some forms of tumors [277,278,279]. Interestingly, it was found that the activity of such compounds depended also on their speciation; for example, **207**, monomeric at neutral pH, showed valuable activity, whereas **212** - a dinuclear specie- resulted inactive. Moreover, their cytotoxicity was not related to their ability to induce ROS generation. Such activity was showed only by complex **207**, whereas **210** and **212** exhibited limited ROS induction. **211** and **212** proved to be able to bind DNA but none of the tested compounds showed significant nuclease activity. This means that imine-copper(II) complexes showing cytotoxicity towards MCF-7 cells do not act by means of ROS generation or nuclease activity, being these two the mechanisms of action previously ascribed to copper (II) complexes [280]. In fact, complexes **208**–**210** acted as superoxide dismutase (SOD) and catalase mimics. This is noteworthy, if we consider that in some cancer cells hypoxia induces an adaptive mechanism providing protection against damage and oxidative stress. In this context, copper (II)-imine complexes acting as SOD mimics may constitute a valid tool for the treatment of hypoxic tumors. 

**Figure 70:**
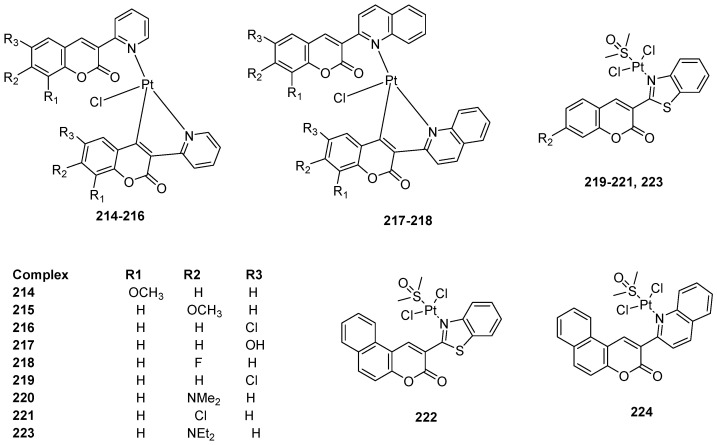
Chemical structure of derivatives **214**–**224**.

In the same year, Qin and collaborators investigated the potential of platinum (II) complexes against cisplatin resistant human lung adenocarcinoma (A549/DDP) and HeLa cells [281]. Cisplatin was used as reference. In this work, eleven complexes between platinum (II) and quinoline-coumarin derivatives (**214**–**224**, Figure 70) were designed, synthesized and tested. All the new complexes showed an improved antineoplastic activity on A549/DDP and HeLa cells when compared to *cis*-Pt(DMSO)_2_Cl_2_ and the parent quinoline-coumarin derivatives, with IC_50_ values ranging between 100 nm and 10.33 μM (75.02 ± 1.18 μM for A549/DDP or 12.09 ± 0.24 μM for HeLa). In addition, compounds **214**–**224** displayed selectivity towards the mentioned cancer cells over other tumoral cell lines and HL-7002 non-tumoral cell line. 

**Figure 71:**
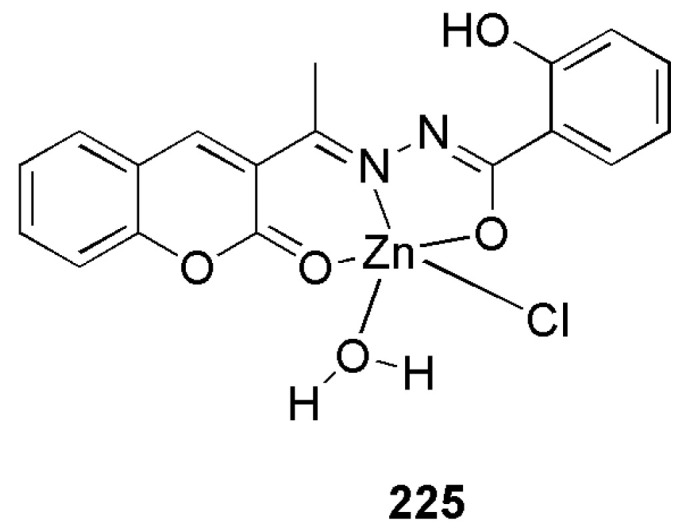
Chemical structure of Zn(II)-coumarin-hydrazone complex **225**.

However, metal-coumarin complexes constitute an interesting tool not only in the pharmacological field but also in the chemical one. As an example of such application, we report here a work from Aslkhademi and collaborators, who designed, synthesized ad characterized a new Zn(II) complex with a coumarin-hydrazone ligand (**225**, Figure 71) [282]. In this case, the complex was evaluated as a catalyst for the synthesis of tetrazoles by means of 3 + 2 cycloaddition between a nitrile and an azide. To this purpose, many experiments were carried out using different nitriles and reaction conditions. In the end, the Zn(II)-coumarin-hydrazone complex resulted to be a suitable catalyst for the synthesis of tetrazoles; the most effective amount of catalyst to employ was 0.05 mmol and substrates bearing electron-donating groups reacted better than the ones with electron-withdrawing substituents. 

Finally, it is worth mentioning the application of metal-coumarin complexes as constituents of sol-gel coatings. Lately, sol-gels are gaining attention due to their chemical stability, homogeneity and technological applications (e.g., reservoir for the prolonged release of antimicrobial substances). In 2013, Jaiswal and co-workers proposed the synthesis of silver-coumarin complexes useful as antibacterial agents in sol-gel coatings [283]. These complexes were based on coumarin-3-carboxylatosilver and its hydroxylated derivatives in 6, 7 and 8. Antimicrobial activity was displayed even at concentration of 0.3% (*w*/*w*). The most active compound showed significative antibiofilm activity at 0.5% and 0.7% (*w*/*w*). These results are encouraging in the perspective of using Ag-coumarin complexes as biomedical coatings.

Table 2 reports some of the coumarins mentioned above, summarizing the biological activity, the molecular target and the origin.

## 4. Synthesis of Coumarin Scaffold

Knoevenagel condensation, Wittig reaction, Claisen rearrangement, Heck reaction, Pechmann condensation and Perkin reaction are widely used methods for the obtainment of coumarin derivatives and have been widely reviewed over the years [284,285]. The increasing interest in coumarins as potential biologically active compounds led to the development of innovative procedures and approaches aimed at the production of coumarins in high yield and in a sustainable manner. In the next paragraphs, we will focus on the most recent advances in coumarin synthesis.

### 4.1. New Approaches in Coumarins Synthesis

#### 4.1.1. Flow Chemistry and Immobilized Reagents

**Scheme 1:**
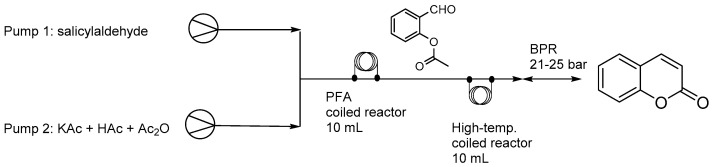
Set-up for the synthesis of coumarins via *O*-acetylation of salicylaldehyde in a continuous flow reactor.

In the past two decades, flow chemistry emerged has a promising synthetic technology, due to the many advantages it offers compared to the traditional batch method. In fact, by using continuous flow reactors it is possible to control reaction parameters, such as temperature, stoichiometry, reaction time and others, very precisely, thus having the possibility to achieve better and more reproducible reactions. Furthermore, the great surface area/volume ratio in continuous flow reactors often leads to better reaction yields. This new technology gives also the possibility to work with hazardous reagents and to perform superheated or pressurized reactions in safer conditions [286,287,288]. The applications of this technology are numerous and the improvement of chemical synthesis of natural compounds is one of them. In 2015, Li and collaborators developed a two-stage synthesis of coumarins via *O*-acetylation of salicylaldehyde [289]. The reaction occurred via *O*-acetylation and intramolecular aldol-type condensation, followed by dehydration and it was performed using two heated coiled reactors (Scheme 1). Using this system, it was possible to reach a 120 g scale production. 

**Scheme 2:**
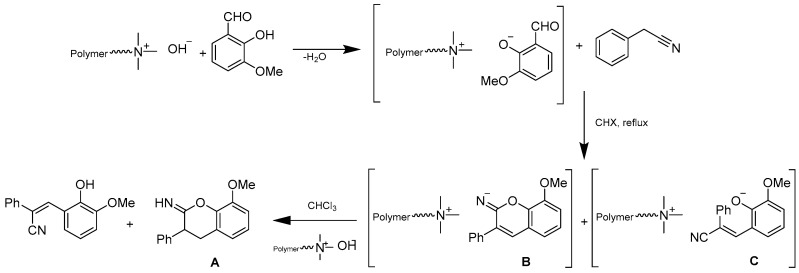
Coumarin synthesis with polymer-supported reagent.

The use of immobilized reagents in organic synthesis is gaining ever more attention, due to the advantages offered by such type of reagents (for example, the simplification of product isolation and purification). This synthetic strategy has seen some applications in the production of natural scaffolds too. In 2018, Mhiri and co-workers proposed a synthetic pathway for coumarin derivatives involving polymer supported reagents [290]. In the mentioned study, coumarin derivative **A** was prepared from 3-metoxy salicylaldheyde using reagents supported in a macroporous ion exchange resin, through the hydrolysis of the two intermediates iminocoumarin **B** and unsaturated nitrile **C** (Scheme 2). 

The first step provided the preparation of polymer supported phenate ions that then reacted with phenylacetonitrile, leading to the formation of iminocoumarin and unsaturated nitrile, which were finally deblocked from the resin with chloroform. The acid hydrolysis of the obtained mixture of compounds **B** and **C** gave coumarin derivative **A** in very good yield (95%). 

#### 4.1.2. Photocatalysis

**Scheme 3:**
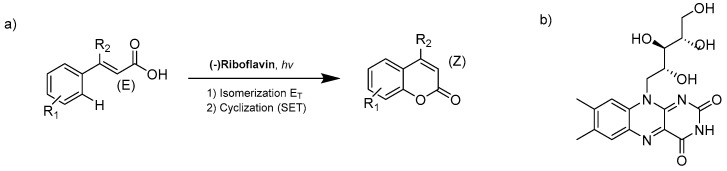
(**a**) Photocatalyzed synthesis of coumarins; (**b**) Chemical structure of (−) Riboflavin.

It is worth speaking about another emerging strategy in organic synthesis: photocatalysis. In 2015, Metternich and Gilmour utilized this approach to emulate coumarins biosynthesis, using (−)-riboflavin as a photocatalyst [291]. In this work, two discrete activation modes of (−)-riboflavin were sequentially exploited to induce the isomerization and cyclisation of *E*-cinnamic acids used as starting material (Scheme 3), thus overcoming the use of phenol-derived and pre-functionalized aryl rings as starting materials. 

**Scheme 4:**
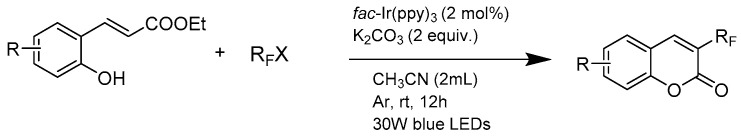
Photoredox-catalyzed synthesis of 3-fluoroalkylated coumarins.

Similarly, in 2019, Song and co-workers reported a one-pot photoredox-catalyzed protocol to achieve a series of 3-fluoroalkylated coumarins starting from *ortho*-hydroxycinnamic esters (Scheme 4) [292]. *ortho*-Hydroxycinnamic esters and BrCF_2_COR’ (or other commercially available perfluoroalkylated radical resources) were exposed to 30W blue LEDs irradiation under argon atmosphere for 12 h.; *fac*-Ir(ppy)_3_ was found to be the best among the tested catalysts, whereas CH_3_CN, DMF, DMSO and THF were all suitable solvents for this reaction. K_2_CO_3_, Et_3_N and DIPEA were all able to neutralize HBr produced during the process. This procedure gave 3-fluoroalkylated coumarins in good yields on milligram and gram scales.

**Scheme 5:**
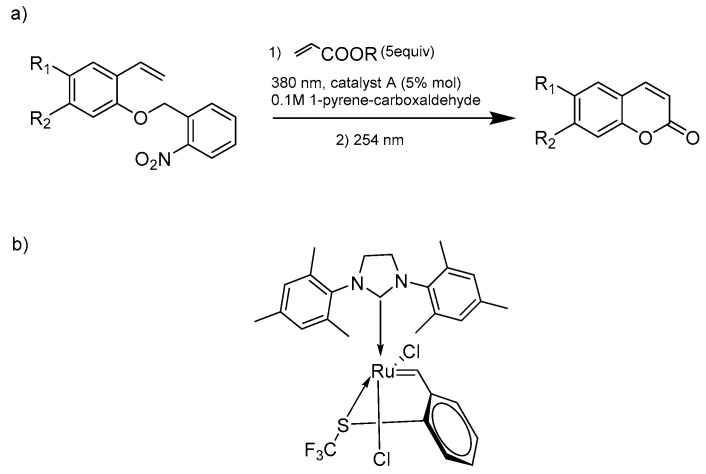
(**a**) Bichromatic synthesis of coumarins; (**b**) chemical structure of catalyst A.

Another example is the bichromatic synthesis of coumarins proposed by Eivgi and collaborators in 2017 [293]. In this work, 2-nitrobenzyl-protected 2-hydroxystyrenes and acrylates underwent a UV-A (380 nm) photoinduced cross metathesis (CM) reaction, in the presence of ruthenium as a catalyst. In this step a 1-pyrenecarboxaldheyde solution was used as a UV filter, without whom the catalyst would be permanently inactivated due to phenol deprotection and phenolate chelation to the ruthenium. After CM, the UV filter was removed and irradiation with UV-C light (254 nm) led to more complex structures, such as coumarins. In fact, irradiation with UV-C light started a three-reactions chain—photodeprotection of the intermediate, E/Z isomerization and cyclization to form the desired coumarins (Scheme 5). 

#### 4.1.3. Solvent-Free Reactions

Chemists usually carry out reactions in solution, following the Aristotelian principle “*Corpora non agunt nisi fluida seu soluta*” or in other and more contemporary words “Compounds do not react unless fluid or if dissolved” [294]. However, this is not always completely accurate, because many solventless reactions proceed efficiently. An enhancement in kinetics, owed to the different concentrations of reactants given the lack of solvents, bring actually to a higher reactivity and, where necessary, milder experimental conditions. At the same time, the absence of solvents provides the chance to heat over the boiling point of the most common solvents and to exploit the benefits of microwave assisted irradiation. The occurrence of efficient solid-state reactions shows that the reacting molecules are able to move freely in the solid state, whereas monitoring of the reaction is possible thanks IR and UV spectra in the solid state [295,296]. The most remarkable advantage of solvent-free reaction, in all probability, is the noteworthy cut in the waste production associated with solvents handling, resulting in a more effective and ecological chemical process. There are three solvent-free techniques: (1) reactions conducted on mineral supports, (2) reaction without any solvent, support or catalyst and (3) solid-liquid phase transfer catalysis [297].

**Scheme 6:**
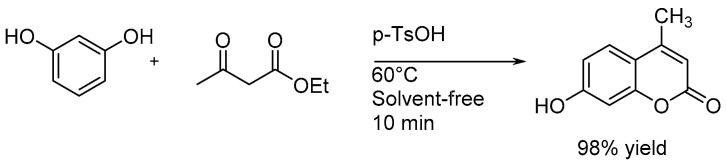
Solvent-free synthesis of 7-hydroxy-4-methylcoumarin.

The experimental conditions required for the classical synthesis of coumarin derivatives are, in some cases, drastic, as in the production of hymecromone via Pechmann condensation where the final compound is obtain by stirring a mixture of resorcinol and ethyl acetoacetate in concentrated H2SO4 for 12–24 h [295]. Solvent-free reactions seemed to be a more ecological option. Consequently, in 2001, Sugino and Tanaka proposed the synthesis of several coumarin derivatives both via Pechmann and Knoevenagel condensation reactions [298]. For example, *p*-toluensolfonic acid was employed as a catalyst and the reaction mixture was heated at 60 °C for 10 min. without solvent, giving after the work-up 7-hydroxy-4-methylcoumarin in 98% yield (Scheme 6).

Knoevenagel condensation was also studied under solventless conditions and efficiently brought to the production of both coumarins and benzocoumarin derivatives [298].

**Scheme 7:**
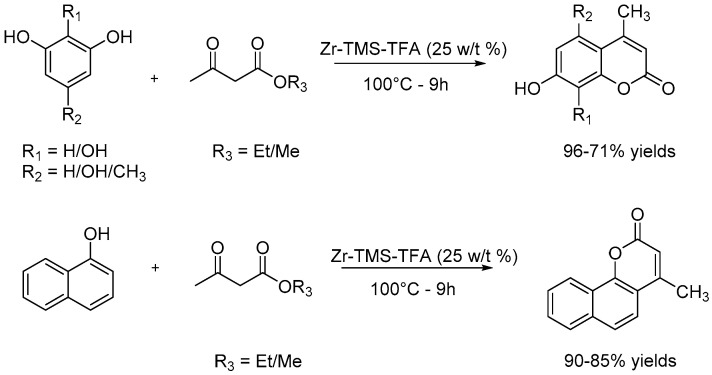
Solvent-free Pechmann condensation with Zr-TMS-TFA as catalyst.

Another methodology was proposed in 2012 by Kalita and Kumar, that exploited the Pechmann reaction using trifluoromethanesulfonic acid functionalized Zr-TMS (Zr-TMS, zirconia-based transition metal oxide mesoporous molecular sieves) catalysts, under solvent-free heterogeneous catalytic conditions. The product selectivity was always found to be about 100% (Scheme 7) [296]. 

**Scheme 8:**
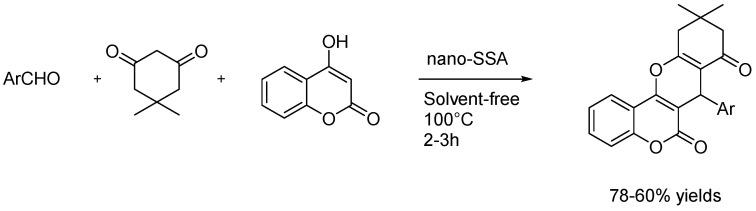
Solvent-free synthesis of hetero-annulated coumarins.

More recently, a novel series of hetero-annulated coumarins was obtained by Ebrahimi and colleagues through a one-pot reaction under solvent-free conditions and evaluated as AChE/BuChE inhibitors. As acidic catalyst nano-silica sulfuric acid was employed and the versatility of the system was assayed on appropriated benzaldehydes, dimedone and 4-hydroxycoumarin (Scheme 8) [299]. Also noteworthy is the application, once again, of a reusable heterogeneous catalyst, which contributed to the general environmental sustainability of the process thanks to the possible recovery and reuse [300]. 

**Scheme 9:**
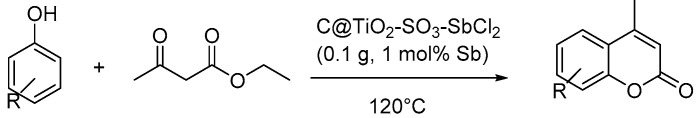
Lewis acid grafted sulfonated carbon@titania composite as catalyst for the Pechmann condensation.

Another solventless procedure worth noting is the one presented by Kour and Paul, which exploited Lewis acid grafted sulfonated carbon@titania composite as catalyst for the Pechmann condensation. This particular kind of catalyst and its efficacy had been already studied by the team for the synthesis of 4*H*-pyrimido[2,1-*b*]benzothiazoles and benzoxanthenones under solvent-free conditions, so the one-pot synthesis of coumarin derivatives was the natural next step (Scheme 9) [301,302]. Because the catalyst reusability contributes to reduce the cost of the practical application processes, the reusability of the catalyst was investigated in the Pechmann condensation. The catalyst, recovered by filtration, after five catalytic runs had a mild decrease in the catalytic activity and exhibited almost the same TG curve as the fresh one, depicting that the present heterogeneous system showed high thermal stability after successive reaction cycles [302].

**Scheme 10:**
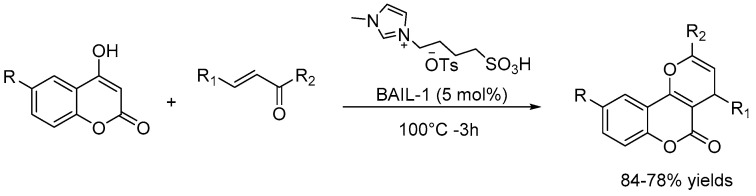
Ionic liquids as an acidic catalyst in the regioselective synthesis of pyrano[3,2-c] coumarins under solvent-free conditions.

In order to obtain a different and ecological procedure for the synthesis of 3-substituted coumarins via Knoevenagel condensation, Ghomi and Akbarzadeh developed a procedure starting from various salicylaldehydes and 1,3-dicarbonyl compounds. They used MgFe_2_O_4_ nanoparticles as an efficient catalyst under solvent-free condition and ultrasound irradiation (Scheme 10). 

Basically, ultrasound irradiation increased the dimension of the single bubbles together with the number of active cavitation bubbles. The resulting effect was expected to be a higher maximum collapse temperature and a faster synthesis of coumarin compounds by Knoevenagel reaction. Comparing ultrasound irradiation to conventional heating the research team demonstrated that the first method allowed to obtain higher yields in shorter times, probably because of the shock wave and microjet generated by the cavitation. Furthermore, with this method, MgFe2O4 nanoparticles were dispersed in the reaction and provided more sites for the construction of cavity over their surface. The catalyst was recovered via magnetic separation and reused up to 6 cycles without relevant loss of activity [303].

The convenience given by the removal of the catalyst by means of an external magnetic field is a significant improvement in the work-up of a reaction mixture. Consequently, another magnetic nano structured catalyst was employed for the synthesis of coumarin nucleus, this time through Pechmann condensation. In 2019, Pakdel and co-workers proposed the synthesis of coumarin derivatives by leveraging Fe_3_O_4_@Boehmite-NH_2_-CoII NPs as a catalyst, under solvent-free conditions that resulted the best choice comparing the results obtained with classic solvents. The catalyst could be recovered easily and reused up to six times. However, no desired coumarin derivatives were observed in the case of phenols bearing electron-withdrawing substituents (such as -Cl and –NO2), behavior attributed to the proposed mechanism of the reaction [304].

In the field of solvent-free reactions, the application of ionic liquids as catalyst for the synthesis of coumarin scaffolds deserves a stand-alone status. Ionic liquids (ILs) have become increasingly popular in the last decades as a safer alternative to classic solvent systems, in a perspective of more sustainable chemical processes. Most ILs possess a number of properties that made them appealing, proving to be non-flammable, to possess a negligible vapor-pressure, that means that solvent evaporation is eliminated and to dissolve a wide range of organic and inorganic compounds [305]. Furthermore, since ionic liquids are low-melting salts, they are made at least from two components which can be varied (the anion and the cation), thus the solvents could be designed with a particular end use in mind or possessing a particular set of properties [306]. However, their use in large quantities is restricted by some limitations like biodegradability, toxicity and high costs [307,308,309]. Mahato and colleagues in 2017 used ionic liquids as an acidic catalyst in the regioselective synthesis of pyrano[3,2-*c*] coumarins under solvent-free conditions. In particular, given the fact that Brønsted acidic ion liquids (BAILs) acquired recognition in the field of catalysis, the research team reported the catalytic effect of 1-butane sulfonic acid-3-methylimidazolium tosylate, [BSMIM]OTs (BAIL-1), on the tandem reaction between 4-hydroxycoumarin and α,β-unsaturated carbonyl compounds for the formation of pyrano[3,2-c]coumarins (Scheme 10) [310,311]. In addition to the high yields and the regioselectivity, this procedure allowed to avoid column chromatography for further purification. Moreover, given the mild experimental conditions, there were no by-products attributable to decomposition or polymerization.

Noroozizadeh and co-workers focused their efforts on the synthesis of bis-coumarins thanks to a different catalyst, acetic acid functionalized poly(4-vinylpyridinum) bromide (APVPB), in continuous of a previous work of the research team on the preparation of Brønsted acidic ionic liquids and solid salts [312,313]. The aim was the development of a novel and efficient procedure for the synthesis of bis-coumarins which lacked the classical drawbacks as the use of solvents, high costs and low yields [314]. 

#### 4.1.4. Microwave Assisted Reactions

**Scheme 11:**
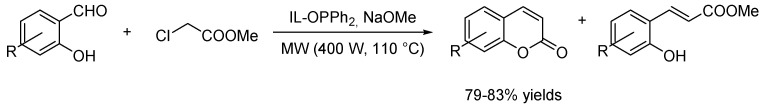
Imidazolium-based phosphinite ionic liquid (IL-OPPh_2_) for the microwave assisted synthesis of coumarins.

Temperature is a key parameter in almost the totality of chemical reactions involving both inorganic and organic compounds. Generally, most organic reactions are performed heating by traditional heat transfer equipment like oil baths, sand baths and heating jackets. The drawbacks associated with these heating techniques are the slowness, the possible development of temperature gradient within the sample and consequent local overheating that could lead to the formation of by-products. Microwave technology started to emerge in inorganic chemistry since the late 1970s and almost a decade after attracted the attention of organic chemists [315]. Compared to the traditional heating, microwave dielectric heating has several advantages: (1) higher heating rates, (2) no direct contact between the source of energy and the reacting chemicals, (3) possible selective heating, because reacting chemicals interact differently with the commonly used microwave radiations and (4) rapid increase of the temperature also above boiling point in pressurized systems [316]. Microwave irradiation is often associated with other technologies, such as ionic liquids and neat reactions (already mentioned in the previous paragraph) [295,317]. In the late 1990s progresses in the classic coumarins synthesis via microwave irradiation were reported by Singh and co-workers, who confirmed a reduction in reaction time of the Pechmann condensation and by Saidi and colleagues, who demonstrated the improvement in the synthesis of pyranocoumarins and furanocoumarins through Claisen rearrangement [317,318]. Ten years later, Valizadeh and Shockravi pushed further the opportunities given by microwave heating combining it with a task-specific imidazolium-based phosphinite ionic liquid (IL-OPPh_2_) for the synthesis of *E*-cinnamates and coumarin derivatives via the one-pot Horner–Wadsworth–Emmons–type reaction (Scheme 11). 

The same reactions were in parallel conducted with traditional heating and microwave irradiation: the former technique showed considerably longer reaction times (13–16 h vs. 10–12 min) and lower yields (65–60% vs. 79–83%). Furthermore, IL-OPPh_2_ worked at the same time as reaction medium and reagent as the research team already demonstrated in a previous study focused on the synthesis of coumarin nucleus via Knoevenagel and Witting reactions in ionic liquids [319,320]. 

In 2016, Fiorito and colleagues merged the advantages of solvent-free reactions with microwave heating for the synthesis of several coumarin derivatives. They started from variously substituted phenols and propiolic acids in the presence of ytterbium triflate hydrate 10% mol as catalyst [321]. During the last thirty years, triflates of metals belonging to the lanthanide series have been regarded as effective water tolerant recyclable Lewis acids. These metals have been discovered to catalyze different carbon–carbon and carbon–heteroatom bond formation reactions, providing the desired products in excellent yields by means of a green chemical approach [322,323]. Furthermore, Wang and colleagues already employed this particular catalyst for the synthesis of 4-methylcoumarins via Pechmann condensation [324]. Fiorito and co-workers developed for the first time a synthetic procedure to obtain different substituted coumarins in 2 min. under microwave irradiation in 91-98% yields [321]. Moreover, the research team recovered the ytterbium triflate catalyst and demonstrated that no loss of activity occurred during several cycles. 

**Scheme 12:**
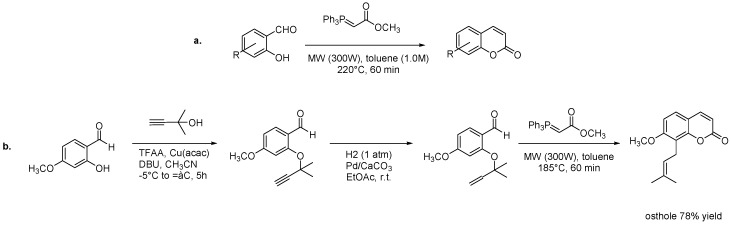
Microwave promoted Witting reaction and total synthesis of osthole. (**a**) general synthetic methodology; (**b**) osthole synthesis.

In the same year, Bouasla et al. compared the efficacy of different heterogeneous acidic catalysts in the Pechmann synthesis of 4-methylcoumarin and 7-hydroxy-4-methylcoumarin when coupled by microwave heating. The efficacy of different Amberylst-type catalyst in the Pechmann synthesis of 7-hydroxy-4-methylcoumarin was already pointed out by Sabou and colleagues in 2005 [325]. Therefore, the catalytic performance of Amberlyst-15 was compared with zeolite H-β and sulfonic acid functionalized hybrid material TS-OS-SO_3_H, performing the reaction under solvent-free conditions, at 130 °C for 20 min in a microwave reaction tube. Amberlyst-15 showed a higher catalytic activity (97% yield against 21–44% obtained with two others catalysts) due to its high density of acid centers [326]. It is also worth mentioning the work of Konrádová et al., who in 2017 developed an interesting protecting-group-free method starting from commercially available aromatic aldehydes and using a microwave promoted Wittig reaction of a stabilized ylide for the synthesis of different natural products, including coumarins. After the optimization of the experimental parameters and the development of a general synthetic methodology for the synthesis of different substitute coumarin derivatives (Scheme 12a), the method was further developed to incorporate a Claisen rearrangement step. In order to demonstrate the synthetic utility and versatility of the developed method, the research team performed the total synthesis of two coumarins, one of which being osthole, a natural coumarin derivative already mentioned in the anti-inflammatory paragraph (Scheme 12b) [327]. 

A broad range of indolo[2,3-*c*] coumarins have been obtained in good yields (46–88%) by Gu and co-workers in 2019 under microwave-assisted base-free intramolecular cross dehydrogenative coupling using palladium catalyst. The advantages of this methodologies compared to classic synthesis of coumarin lactone ring were both atom economy and step efficiency, combined with C-H/C-H coupling without pre-functionalization [328].

## 5. Conclusions

The coumarin nucleus has been gaining increasing attention in the last years because of the variety of its applications both in medicinal chemistry and in agri-food sectors. In this review, we presented a collection of recent works that highlight the wide range of pharmacological activities ascribable to coumarins (antioxidant, antibacterial, antifungal, antiviral, anti-proliferative, anti-inflammatory, antidiabetics, anticoagulant and anti-neurodegenerative) and the possibility to easily modify and decorate this natural scaffold to perform structure activity relationships studies. The coumarin nucleus possesses some fundamental properties that ensure it an advisable role in the design of new biologically active derivatives, mainly due to its stability, low molecular weight and to the easiness to decorate it for increasing pharmacodynamic and pharmacokinetic properties.

Coumarin scaffold has been a valuable source of inspiration in the design of novel biologically active compounds and its role as a starting point is very attractive. This natural core is present in a number of currently available drugs used in the treatment of various diseases. For instance, the use of warfarin, acenocumarol and phenprocoumon in the treatment and prevention of thromboembolic diseases is now well established. Other examples include the use of hymecromone as choleretic agent and that of the antihelminthic haloxon in veterinary medicine. Even though the coumarin nucleus presents an impressive number of biological activities, its presence among marketed drugs is not widespread yet. Further efforts are needed in order to achieve coumarin-based compounds with appreciable pharmacokinetic properties, along with high efficacy and a low toxicity profile.

Moreover, in this review we discussed coumarin photoproperties and their applications as photocleavable protecting groups and fluorescent probes. Finally, we analyzed the latest updates in coumarin synthesis from both chemical and technological points of view. One of the most interesting approaches that emerged from this study is the design and use of coumarin-containing hybrid compounds.

It is our hope that this review will contribute to further development in the investigation and exploitation of coumarins’ potential. 

## Figures and Tables

**Table 1:** Classification of natural coumarins.

Class	General Structure	Examples
**Simple coumarins**	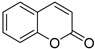	Osthole (neuroprotective, osteogenic, immunomodulatory, anticancer, hepatoprotective, cardiovascular protective and antimicrobial) [13]
**Furanocoumarins**	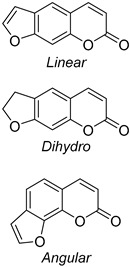	Psoralen (antifungal) [14] Antoghenol (antibacterial) [15] Columbianedin (anti-inflammatory) [15]
**Pyranocoumarins**	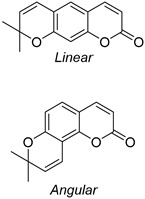	Grandivittin (antibacterial) [16] Inophyllum A, B, C, E, P, G_1_, andG_2_ (antiviral) [17]
**Biscoumarins**	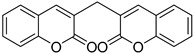	Dicoumarol (anticoagulant) [18]
**Isocoumarins**	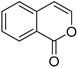	Thunberginols (antidiabetic) [19]
**Phenylcoumarins**	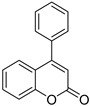	Isodispar B (anti-inflammatory) [20]

**Table 2:** Summary table.

Structure	Biological Activity	Molecular Target	Name/Number	Ref.	Origin
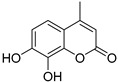	Antioxidant	Free radicals	**3**	[30]	Synthetic
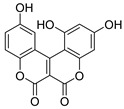	Antioxidant	Free radicals	**9**	[35]	Synthetic
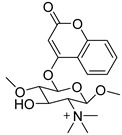	Antioxidant	Free radicals	**10d**	[36]	Synthetic
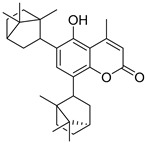	Antioxidant	Free radicals	**15**	[38]	Synthetic
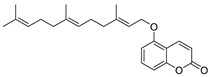	Antiprolifera-tive	DNA, chromatin	**18**	[45]	Natural
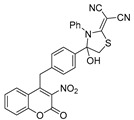	Antiprolifera-tive	Topoisomerase 1	**20**	[47]	Synthetic
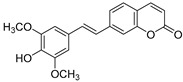	Antiprolifera-tive	Tumor-suppressor protein p53	**21**	[49]	Synthetic
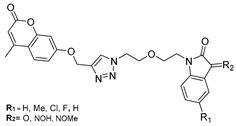	Anticancer		**22a-l**	[50]	Synthetic
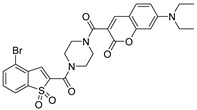	Anticancer	STAT3	**23**	[55]	Synthetic
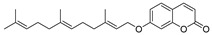	Anticancer	Carbonic Anhydrase IX and XII	**24**	[70]	Natural
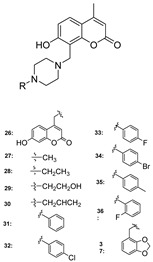	Anticancer	Carbonic Anhydrase IX and XII	**26**–**37**	[71]	Synthetic
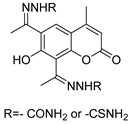	Antibacterial/antifungal	*S. aureus*, *E. coli*, *P. aeruginosa*, *A. niger*, *A. flavus*, *C. albicans*	**38**,**39**	[79]	Synthetic
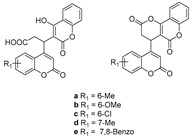	Antibacterial	*S. aureusus*, *E.faecalis*, *E. coli*	**40**,**41a–e**	[80]	Synthetic
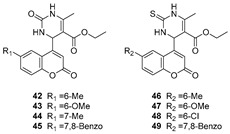	Antibacterial	*S. aureus* DNA gyrase	**42**–**49**	[82]	Synthetic
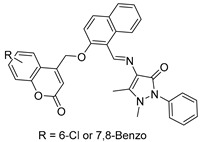	Antibacterial	*S. aureus*	**50**,**51**	[83]	Synthetic
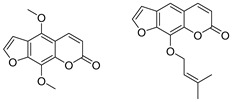	Modulator of antibiotics	n.g.	isopimpinellin imperatorin	[85]	Natural
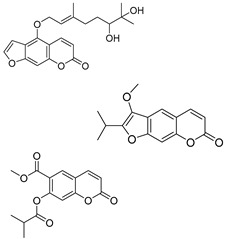	Antibacterial	*S. aureus*, *S. epidermidis*, *P. aeruginosa*, *E. cloacae*, *K. Pneumoniae*, *E. coli*	6′,7′-dihydroxybergamottin, peucedanin, officinali isobutyrate	[86]	Natural
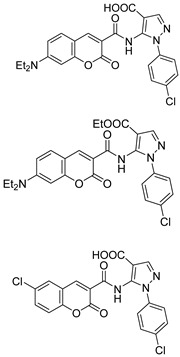	Antibacterial	*S. aureus*, *L. monocytogenes*, *E. coli*, *Salmonella*—inhibitors of Topo II and Topo IV	**52**–**54**	[87]	Synthetic
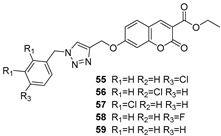	Antifungal	*C. albicans -*P450 cytochrome lanosterol 14α-demethylase	**55**–**59**	[94]	Synthetic
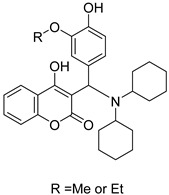	Antifungal	*C. albicans*—inhibition ergosterol biosynthesis by binding lanosterol 14a-demethylase	**60**,**61**	[95]	Synthetic
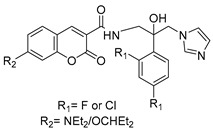	Antifungal	Several *Candida* strains—mode of action involving CYP51 and additional unidentified mechanism	**62**	[96]	Synthetic
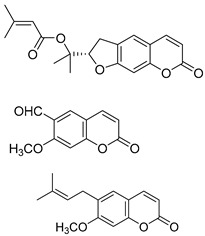	Antifungal	*C. albicans*	marmesin senesioate, suberosin, crenulatin	[97]	Natural
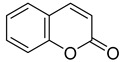	Antifungal	*C. albicans—antibiofilm action*	coumarin	[100]	Natural
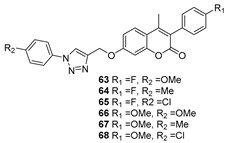	Antifungal	*F. oxysporum*, *A. flavus*, *A. niger*	**63**–**68**	[102]	Synthetic
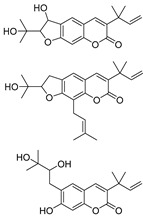	Antiviral	Anti-HIV reverse transcriptase	**69**–**71**	[113]	Natural
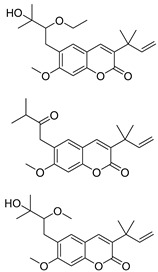	Antiviral	HIV reverse transcriptase	**72**–**74**	[116]	Natural
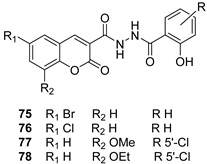	Antiviral	HIV1-IN	**75**–**78**	[117]	Synthetic
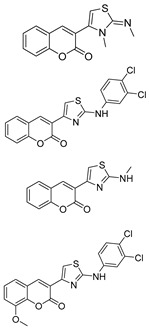	Antiviral	H1N1 virus	**79**–**82**	[125]	Synthetic
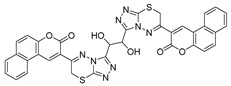	Antiviral	Influenza A virus	**83**	[128]	Synthetic
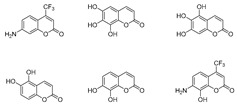	Antiviral	Influenza A/PR8/H1N1 viruses—probably could affect intracellular redox-sensitive pathways	**84**–**89**	[129]	Synthetic
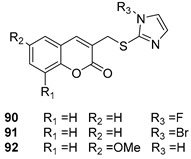	Antiviral	Hepatitis C virus	**90**–**92**	[130]	Synthetic
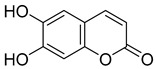	Antiviral	Hepatitis B virus	esculetin	[131]	Natural
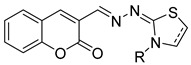	Antiinflamma-tory	COX2	**93**–**100**	[143]	Synthetic
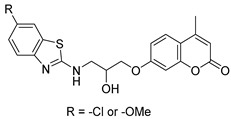	Antiinflamma-tory	5-LOX	**101**,**102**	[146]	Synthetic
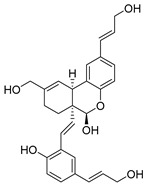	Antiinflamma-tory	NO production	**103**	[147]	Natural
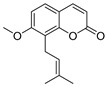	Antiinflamma-tory	NF-kB and p38/MAPK signaling pathways	osthole	[150]	Natural
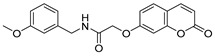	Antiinflamma-tory	NF-kB signaling pathways	**104**	[151]	Synthetic
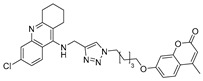	Anti-Alzhemer’s disease	AChE, BuChE, BACE1	**105**	[162]	Synthetic
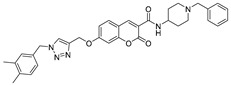	Anti-Alzhemer’s disease	AChE, BuChE, BACE1, metal chelation	**107**	[163]	Synthetic
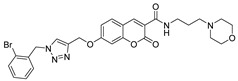	Anti-Alzhemer’s disease	AChE, BuChE, BACE1	**108**	[163]	Synthetic
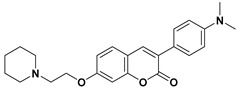	Anti-Alzhemer’s disease	AChE, BuChE	**111a**	[166]	Synthetic
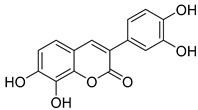	Anti-Alzhemer’s disease	AChE, BuChE, MAO-B	**117**	[167]	Synthetic
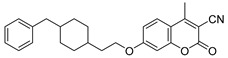	Anti-Alzhemer’s disease	AChE, BuChE, MAO-B, β-amyloid aggregation	**136**	[169]	Synthetic
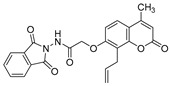	Anticonvulsant	GABAergic system	**148**	[180]	Synthetic
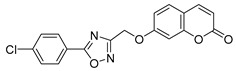	Anticonvulsant	BDZ receptor	**152d**	[183]	Synthetic
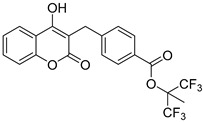	Anticoagulant	VKOR inhibitor	Tecarfarin(ATI-5923)	[195,196,197,198]	Synthetic
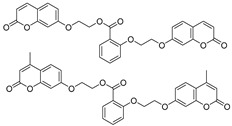	Anticoagulant	n.g.	**154**,**155**	[199]	Synthetic
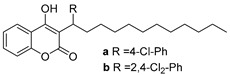	Anticoagulant	murine VKORC1 inhibitor	**156**	[200]	Synthetic
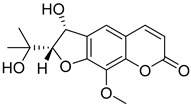	Anticoagulant	n.g.	**157**	[203]	Natural
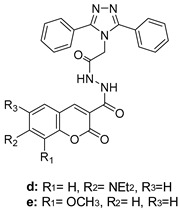	Antidiabetic	α-glucosidases	**158d**,**e**	[207]	Synthetic
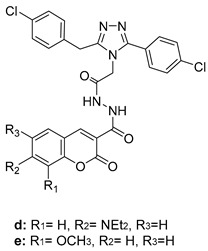	Antidiabetic	α-glucosidases	**159d**,**e**	[207]	Synthetic
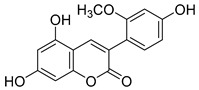	Antidiabetic	α-glucosidases	**165**	[214]	Synthetic
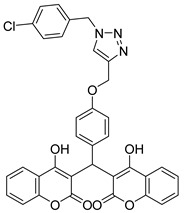	Antidiabetic	α-glucosidases	**168c**	[215]	Synthetic
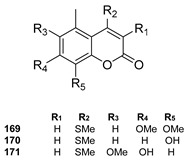	Antidiabetic	Insulin release	**169**–**171**	[217]	Synthetic
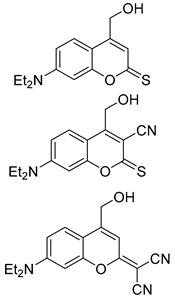	Photocleavable protecting groups	-	**172**–**174**	[231,232]	Synthetic
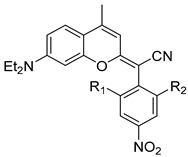	Photocleavable protecting group	-	**175**	[224]	Synthetic
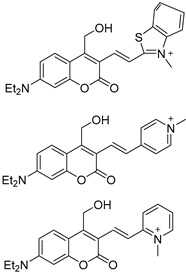	Photocleavable protecting groups	-	**176**–**178**	[233]	Synthetic
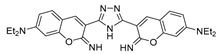	Ionic probe	GHS (in the presence of Cu^2+^) and ClO^−^	**179**	[236,237]	Syntehtic
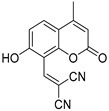	Ionic probe	ClO^−^	**180**	[238]	Synthetic
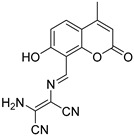	Ionic probe	ClO^−^, Cu^2+^	**181**	[239]	Synthetic
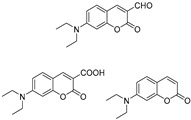	Ionic probe	ClO^−^	**182**–**184**	[240]	Synthetic
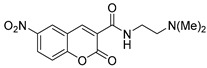	Ionic probe	Cu^2+^	**185**	[241]	Synthetic
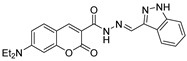	Ionic probe	Cu^2+^	**186**	[242]	Synthetic
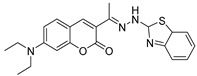	Ionic probe	Cu^2+^	**187**	[243]	Synthetic
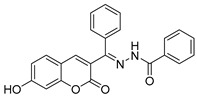	Ionic probe	Cu^2+^, Al^3+^, Arg, Lys, Cys, Hcy, GSH	**188**	[244]	Synthetic
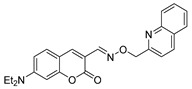	Ionic probe	Pd^2+^	**189**	[245]	Synthetic
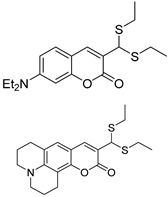	Ionic probe	Hg^2+^	**190**,**191**	[246]	Synthetic
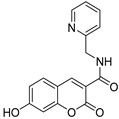	Ionic probe	Fe^3+^	**192**	[247]	Synthetic
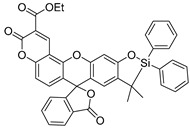	Ionic probe	F^−^	**193**	[248]	Synthetic
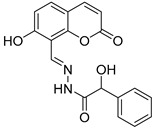	Ionic probe	F^−^, Ca^2+^	**194**	[249]	Synthetic
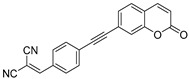	Ionic probe	F^−^, CN^−^	**195**	[250]	Synthetic
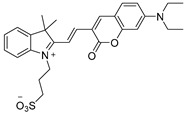	Probe	HSO_3_^−^, SO_3_^2−^ ion	**196**	[251]	Synthetic
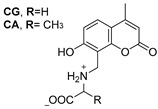	Probe	HSO_4_^−^ and SO_4_^2−^	CG, CA	[256]	Synthetic
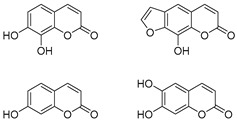	Antibacterial	*R. Solanacearum*, genes *fliA*, *flhC*, biofilm formation	umbelliferone, esculetin, daphnetin, xanthotol	[261]	Natural
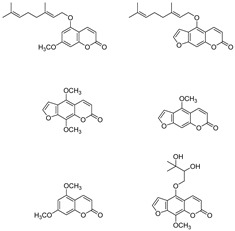	Fungicide	*Colletotrichum* sp.	**197**–**202**	[265]	Natural
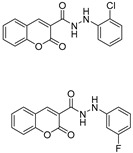	Fungicide	*Botrytis cinerea*, *Alternaria solani*, *Gibberella zeae*, *Rhizoctorzia solani*, *Cucumber anthrax* and *Alternaria*	**203**,**204**	[268]	Synthetic
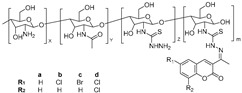	Fungicide:	*Alternaria solani sorauer*, *Fusarium oxysporum* f.sp. *vasinfectum* and *Fusarium moniliforme*	**205a**–**d**	[273]	Synthetic
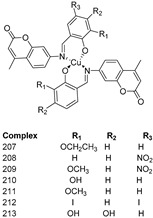	Anticancer	MCF-7 human breast cancer cells	**207**–**213**	[276]	Synthetic
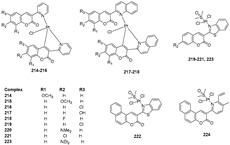	Anticancer	Human lung adenocarcinoma (A549/DDP) and HeLa cells	**214**–**224**	[281]	Synthetic
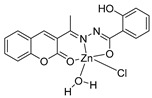	Catalyst		**225**	[282]	Synthetic
